# Mechanisms of Vascular Smooth Muscle Contraction and the Basis for Pharmacologic Treatment of Smooth Muscle Disorders

**DOI:** 10.1124/pr.115.010652

**Published:** 2016-04

**Authors:** F.V. Brozovich, C.J. Nicholson, C.V. Degen, Yuan Z. Gao, M. Aggarwal, K.G. Morgan

**Affiliations:** Department of Health Sciences, Boston University, Boston, Massachusetts (C.J.N., Y.Z.G., M.A., K.G.M.); Department of Medicine, Mayo Clinic, Rochester, Minnesota (F.V.B.); and Paracelsus Medical University Salzburg, Salzburg, Austria (C.V.D.)

## Abstract

The smooth muscle cell directly drives the contraction of the vascular wall and hence regulates the size of the blood vessel lumen. We review here the current understanding of the molecular mechanisms by which agonists, therapeutics, and diseases regulate contractility of the vascular smooth muscle cell and we place this within the context of whole body function. We also discuss the implications for personalized medicine and highlight specific potential target molecules that may provide opportunities for the future development of new therapeutics to regulate vascular function.

## I. Introduction

### A. Scope and Limitations

The smooth muscle cells of blood vessels display considerable plasticity in their phenotype. In healthy, young blood vessels, the phenotype is largely contractile and blood pressure is well autoregulated. However, during the life span of an individual, vascular cells can switch to a synthetic, largely noncontractile phenotype in response to proinflammatory stimuli, diet or other factors that result in the development of atherosclerosis or vessel remodeling. We will not focus on these processes here but refer the reader to several recent reviews on this topic (Heusch et al., 2014; Brown and Griendling, 2015; Tabas et al., 2015).

Here we will focus on the contractile phenotype, which also can display plasticity of function through a range of more subtle adaptations to aging, biomechanical stress, and vasoactive physiologic and pathophysiologic molecules. The current review will focus on these responses and especially focus, as a prototype disease of contractile vascular smooth muscle, on the complex role of this cell type in hypertension and where many opportunities exist for the exploration of untapped potential therapeutic targets.

### B. Overview of Regulation of Blood Pressure/Vascular Tone

#### 1. Guyton View of Regulation Blood Pressure, Kidney Role, Volume Regulation.

In humans, the diagnosis of hypertension is widespread, but typically asymptomatic; 20–50% of the world’s population has hypertension and in the United States ∼30% of the population is hypertensive (Hajjar et al., 2006). Furthermore, hypertension is a major risk factor for cardiovascular disease, stroke, and end-stage renal disease, and thus, there is significant morbidity and mortality associated with this disease. Because blood pressure (BP) is related to the cardiac output (CO) and systemic vascular resistance (SVR) by the equation BP = CO × SVR, increases in either CO or SVR should produce hypertension. Thus, although the molecular mechanism(s) that produce hypertension would be expected to be relatively straightforward, over 50 years of investigation have not defined the molecular mechanism(s) that underlies this medical condition.

**Fig. 1.**
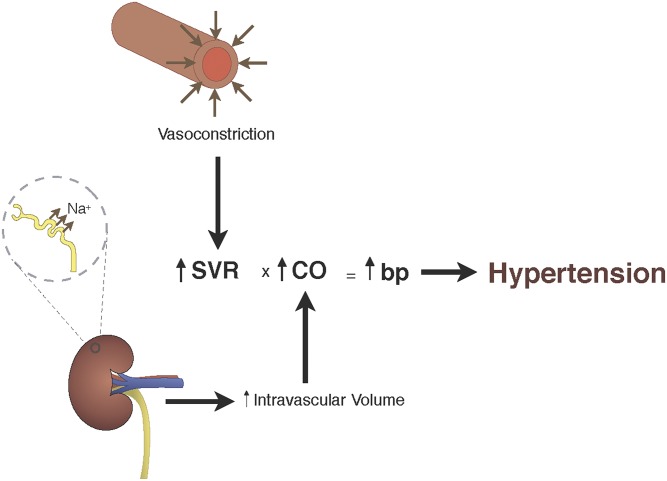
SVR versus kidney: Blood pressure is the product of systemic vascular resistance and cardiac output (BP = SVR × CO). Changes in Na^+^ reabsorption will increase or decrease intravascular volume and result in an increase or decrease cardiac output, which will alter blood pressure. Similarly, alterations in vascular tone can either increase or decrease SVR, which leads to an increase or decrease in blood pressure (see text for details).

The control of blood pressure is an integrated response that includes regulation by neural receptors, hormones, and renal fluid balance (Guyton, 1991). However, the handling of sodium within the kidney is well accepted to be the major factor that regulates blood pressure **(**Fig. 1), and hence, in the pathogenesis of hypertension renal Na^+^ excretion, which regulates intravascular volume, is the primary determinant of cardiac output (CO) and therefore blood pressure (Guyton, 1991). The role of control of intravascular volume by the kidney for the pathogenesis of hypertension is supported by the results of an elegant series of studies by Lifton’s group (reviewed in Lifton et al., 2001). These investigators demonstrated that in humans, rare genetic causes of hypertension all arise from a defect in the handling of Na^+^ in the kidney; mutations that increase Na^+^ reabsorption (volume expansion) result in severe hypertension, whereas mutations that decrease Na^+^ resorption (volume contraction) produce hypotension. We will not discuss the well-accepted role of renal fluid balance in regulation blood pressure, because this topic has been the subject of a number of reviews (Lifton et al., 2001; Oparil et al., 2003; Coffman and Crowley, 2008; Johnson et al., 2008).

#### 2. Recent Direct Confirmation of Changes in Vascular Tone/Resistance Related to Changes in Systemic Vascular Resistance and Blood Pressure and the Importance of Vascular Smooth Muscle Contraction in both Normal Physiology and Pathophysiology—Hypertension.

More than 90% of patients are diagnosed with essential hypertension, or hypertension of unknown etiology (Oparil et al., 2003). Fortunately, despite the lack of a clear mechanism, there are a number of classes of antihypertensive agents that effectively lower blood pressure. Intuitively, one would expect that changes in vascular tone would result in changes in systemic vascular resistance (SVR) and result in either hyper- and/or hypotension. And, although a number of the classes of antihypertensive agents target the vascular smooth muscle [*α*-blockers, angiotensin converting enzyme (ACE) inhibitors, angiotensin receptor blockers (ARBs), calcium channel blockers (CCBs)], until recently, there was little experimental evidence consistent with the regulation of vascular tone being an important factor for the molecular mechanism that produces hypertension (Fig. 1). However, a number of studies have demonstrated the importance of changes in vascular reactivity or the regulation of vascular smooth muscle contraction and/or vascular tone for the control of blood pressure. For these experiments, investigators have genetically modified a mouse to produce abnormalities in the regulation of vascular tone and/or vascular dysfunction; these mouse models include the BKCa^2+^ channel *β*1 subunit knockout (KO) (Brenner et al., 2000), estrogen receptor *β* KO (Zhu et al., 2002), vascular smooth muscle cell Sur2 K(ATP) channel KO (Chutkow et al., 2002), endothelial nitric oxide synthase (eNOS) KO (Huang et al., 1995), RGS2 KO (Tang et al., 2003), PKGI KO (Tang et al., 2003), PKGI*α* leucine zipper mutant (Michael et al., 2008), and the MYPT1 KO (Qiao et al., 2014). All of these mice have both vascular dysfunction and hypertension, and these data suggest that vascular dysfunction produces hypertension. However, in these transgenic models, vascular dysfunction within the kidney could alter fluid balance and a resulting increase in intravascular volume and the resulting increase in CO could be responsible for producing hypertension. The most compelling argument that isolated vascular dysfunction results in hypertension are the results of Crowley et al. (2005). These investigators demonstrated that mice with a KO of the angiotensin type 1 (AT1) receptor were hypotensive. Furthermore, these investigators produced mice with the KO of the AT1 receptors in the kidney with normal AT1 expression in the peripheral vasculature, as well as the KO of AT1 receptors in the peripheral vascular smooth muscle with normal AT1 expression in the kidney. The blood pressure in these two strains was equal and intermediate between the AT1 KO and wild-type (WT) mice. These results demonstrate that in isolation, an abnormality in the regulation of vascular smooth muscle contraction produces a change in blood pressure, and therefore, an isolated increase in vascular smooth muscle tone will produce hypertension. Thus the regulation of vascular smooth muscle contraction is important in both health and disease.

#### 3. Racial Differences/Personalized Medicine.

Further complicating investigation of the mechanism underlying the pathogenesis of hypertension are racial differences in the effectiveness of the various classes of antihypertensives (Cushman et al., 2000; Johnson, 2008; Gupta, 2010), including the response to *β*-blockers, ACE inhibitors, and ARBs. White compared with black patients with hypertension are more likely to respond to *β*-blockers, ACE inhibitors, and ARBs, whereas for black patients, treatment with a diuretic or calcium channel blocker (CCB) is more likely to be effective (Johnson et al., 2008). Additionally, there also appears to be regional differences in the response to antihypertensive agents; there is a 10 state region in the Southeastern U.S., referred to as the Stroke Belt, in which the mortality from cerebral vascular accidents is 10% greater than the rest of the country. In this region, compared with the rest of the U.S., treatment of hypertension with diuretics, *β*-blockers, ACE inhibitors, and clonidine is less effective, whereas there is no difference in the effectiveness of CCBs and prazosin (Cushman et al., 2000). After controlling for race, the differences in the therapeutic success of diuretics and clonidine is still present. Furthermore, for black patients with hypertension in this region, similar to the rest of the U.S., CCBs are more likely to control blood pressure and the effectiveness of *β*-blockers and prazosin therapy is poor.

These racial differences in response to therapy are also present for the treatment of heart failure. Analysis of the results of the V-HeFT (Vasodilator-Heart Failure) trial demonstrated that treatment of black patients with heart failure with the combination of hydralazine and isosorbide dinitrate reduced mortality, whereas this regimen did not change mortality compared with placebo for white patients (Carson et al., 1999). In contrast to these results, treatment of heart failure with enalapril reduced mortality in white, but not black, patients, and in white patients, enalapril produced a larger reduction in blood pressure and regression of cardiac size than hydralazine and isosorbide dinitrate (Carson et al., 1999).

These racial and regional differences in the response to antihypertensive regimens could be due to polymorphisms. A number of genome wide-association studies (GWAS) have investigated this question (reviewed in Cushman et al., 2000; Johnson et al., 2008), and these studies as well as their implications will be discussed later in this review. However, changes in the vascular smooth muscle phenotype could be responsible for diversity in the effectiveness of the different classes of antihypertensive agents. Defining the role of the vascular smooth muscle phenotype in the pathogenesis of hypertension could identify novel therapeutic targets, which could be exploited in rational drug design. Furthermore, comparing the vascular smooth muscle phenotype between races and regions could potentially define the mechanism that governs the heterogeneity in the response to antihypertensive therapy and form the basis for an individualized approach for selecting an effective antihypertensive regimen.

## II. Regulation of Ca^2+^

### A. Ca^2+^ Determines Vascular Smooth Muscle Cell Contractility and Phenotype

Vascular smooth muscle cells (VSMC), like all other muscle cells, depend on Ca^2+^ influx to initiate contraction. However, the VSMC intracellular Ca^2+^ concentration does not only determine the contractile state, but also affects the activity of several Ca^2+^ dependent transcription factors and thereby determines VSMC phenotype. To govern the various Ca^2+^-dependent functions and in reaction to different stimuli, VSMCs use a variety of plasmalemmal and sarcoplasmic reticulum (SR) Ca^2+^ channels to produce a large repertoire of Ca^2+^ signals, which differ in their spatial and temporal distribution (reviewed by Amberg and Navedo, 2013). These signals range from cell-wide changes in [Ca^2+^] to highly localized Ca^2+^ entry or release events. Ca^2+^ can enter the cell from the extracellular space or be released from the largest intracellular Ca^2+^ store, the sarcoplasmic reticulum (SR). Extracellular Ca^2+^ influx is mainly mediated by the opening of voltage dependent L-type Ca^2+^ channels (LTCC), but there are a number of other channels that modulate intracellular Ca^2+^, including transient receptor potential (TRP) cation channels. Because of their high single-channel conductance and expression in VSMCs, LTCCs have the largest influence on global [Ca^2+^]_i_, and their activity largely determines the VSMC’s contractile state and ultimately vessel diameter (Knot and Nelson, 1998). In response to agonist activation of SR-bound inositol trisphosphate (IP3) or ryanodine receptors (RyR), Ca^2+^ is released into the cytoplasm from the SR. Local Ca^2+^ signals from the plasmalemma or the junctional SR can augment or oppose increases in global Ca^2+^ through the activation of Ca^2+^-dependent ion channels and their regulatory signaling molecules that ultimately affect plasma membrane potential and therefore LTCC activity.

### B. Compartmentalization of Ca^2+^ Signaling

**Fig. 2.**
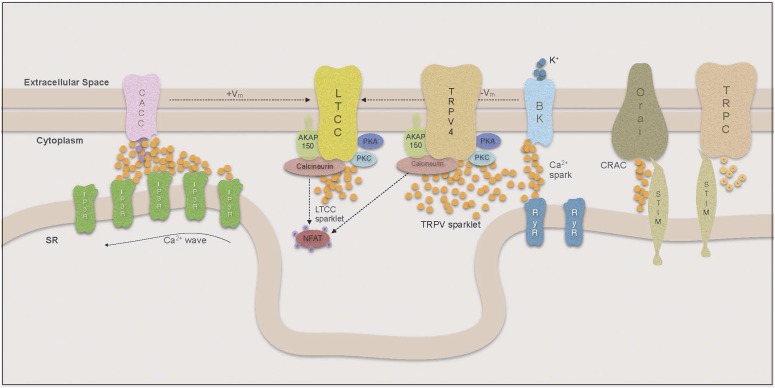
Compartmentalization of Ca signaling.

The concept of Ca^2+^ compartmentalization was introduced when it was demonstrated that local increases in Ca^2+^ could activate the contractile apparatus without influencing other Ca^2+^-dependent signaling pathways (Karaki, 1989). Ca^2+^ is slow to diffuse across the cytoplasm (Berridge, 2006) and a large flux of Ca^2+^ is required to achieve the high Ca^2+^ concentration necessary for activation of Ca^2+^-dependent processes. Therefore, to compartmentalize and regulate Ca^2+^ signals, VSMCs arrange their organelles in a fashion that limits the space for diffusion and thereby increases the effect of local changes in [Ca^2+^] (Kargacin, 1994; Poburko et al., 2004) (Fig. 2). The effects of Ca^2+^ entry hence depend on the way that organelles, Ca^2+^ pumps, channels, and Ca^2+^-dependent signaling molecules are organized in signaling microdomains around the source of the Ca^2+^ signal, as well as its duration and amplitude. More on the organization of such microdomains in VSMCs and how they affect VSMC contractility and phenotype can be found in the review on regulation of cellular communication by signaling microdomains by Billaud et al. (2014).

#### 1. Ca^2+^ Sparklets.

Local increases in cytoplasmic Ca^2+^ resulting from influx through single or small clusters of LTCCs are called Ca^2+^ sparklets (reviewed by Navedo and Amberg, 2013). Because of the steep voltage sensitivity of LTCCs, the sparklet frequency and persistence are closely linked to membrane potential. Thus local changes in membrane potential will result in alterations of local sparklet activity, whereas cell-wide depolarization leads to extensive opening of LTCCs and global influx of Ca^2+^ (Navedo et al., 2005; Amberg et al., 2007). Increases in [Ca^2+^]_i_ and Ca^2+^ sensitivity of the contractile apparatus in VSMCs are considered hallmarks of essential hypertension, and it has been widely assumed that the increase in intracellular Ca^2+^ is mediated by increased influx through LTCCs. Consistent with this are results in the rat where banding was used to produce a sudden high intravascular pressure in the right renal artery. After only 2 days, VSMCs from the right renal artery showed increased expression of *α*_1C_ subunits of the LTCC and increased Ca^2+^ currents compared with VSMCs from the left renal artery (Pesic et al., 2004). However, the ratio of right renal artery/left renal artery *α*_1C_ subunit expression decreased over time, which may indicate a dynamic adjustment to this sudden pressure overload occurring within the VSMCs.

Surprisingly, overall LTCC expression and cell-wide Ca^2+^ influx was recently found to be decreased in a mouse model of essential hypertension (Tajada et al., 2013). However, although there was a decrease in the number of LTCCs present on the plasma membrane, the LTCCs showed increased local sparklet activity. These investigators demonstrated that fewer, but highly active, LTCCs were able to increase [Ca^2+^]_i_ locally as well as cell-wide. The activity of Ca^2+^ sparklets has been shown to depend on whether the LTCC is part of a pentad complex bound to the plasma membrane by the scaffolding protein AKAP150 (Navedo et al., 2008). LTCCs that are not coupled in such complexes have a higher probability of producing stochastic sparklets with low flux and short duration, whereas AKAP-associated channels can produce high-activity persistent sparklets.

The dynamics of these persistent sparklets are regulated by kinases and phosphatases that are targeted to a subpopulation of LTCCs by the plasmalemmal anchor AKAP150. Under physiologic conditions in these signaling microdomains, the formation of persistent sparklets mainly relies on protein kinase C (PKC) activity and is counteracted by the serine phosphatase calcineurin. In pathologic conditions such as diabetes, however, protein kinase A (PKA) becomes a mediator of enhanced sparklet activity (Navedo et al., 2010). In a study by Navedo et al. (2008) it was shown that the inhibition of cytoplasmic calcineurin with cyclosporine A in AKAP^−/−^ mice had no effect on LTCC sparklet activity, whereas the inhibition of AKAP150-anchored calcineurin in wild-type mice yielded an increase in persistent sparklets. This confirmed the hypothesis that there was a negative relationship between calcineurin and LTCC sparklet activity but highlighted the importance of calcineurin being targeted to the plasmalemma by AKAP150. The relevance of PKC interaction with LTCCs in the development of ATII-induced hypertension has been demonstrated in a number of experiments, in which not only the KO of PKC but also the ablation of AKAP150 lead to an inability of ATII infusion to produce hypertension (Navedo et al., 2008). In this model, the level of cellular PKC was unchanged in AKAP150^−/−^ VSMCs. These data suggest that recruitment of PKC to the LTCC by AKAP150 is crucial for the development of this form of hypertension. AKAP150 is also thought to play a role in the functional coupling of LTCCs to each other, which amplifies Ca^2+^ influx and is, similar to persistent sparklet activity, increased in hypertension (Nieves-Cintron et al., 2008). Although the mechanism of coupled gating is still under investigation, a model has been proposed by which coupled gating is mediated by calmodulin (CaM)-dependent interactions between the carboxy-terminals of AKAP150-coupled LTCCs and is increased with PKC activation and calcineurin inhibition (Navedo et al., 2010; Cheng et al., 2011).

It should be noted, that in some studies, there was significant PKC activation in agonist-mediated vasoconstriction, but not in pressure-mediated vasoconstriction (Jarajapu and Knot, 2005; Ito et al., 2007), suggesting that PKC has a negligible role in myogenic tone. However, other groups reported PKC involvement in the modulation of the arteriolar myogenic response to increased intravascular pressure (Hill et al., 1990). This study demonstrated that inhibition of PKC led to inhibition of the myogenic response, whereas a stimulator of PKC activity increased myogenic responsiveness.

In a recent study (Mercado et al., 2014), investigators demonstrated that AKAP150-recruited PKC also regulates the activity of Ca^2+^-permeable, nonselective TRPV4 channels. These channels can produce Ca^2+^ sparklets with 100-fold higher Ca^2+^ flux compared with LTCCs, yet they have been linked to VSMC relaxation (Earley et al., 2009). This association results from the high Ca^2+^ flux that enables TRPV4 to stimulate SR-membrane bound RyRs in relative proximity to the plasmalemma as a form of Ca^2+^-induced Ca^2+^ release (CICR) found in VSMCs.

In contrast to TRPV4 sparklets, LTCC sparklet flux is much lower and therefore not sufficient to trigger Ca^2+^ release from the SR, but overall LTCC sparklet activity is higher and hence LTCCs have a much greater effect on global Ca^2+^. Through the effect on global Ca^2+^, LTCC sparklet activity determines the rate at which the SR can refill its Ca^2+^ stores. However, neither the SR Ca^2+^ content nor the number and amplitude of SR Ca^2+^ release events appear to be directly linked to LTCC sparklet activity (Collier et al., 2000; Essin et al., 2007).

Other important members of the TRP channel family include TRPC1, TRPC3, TRPC6, and TRPM4. They have been found to have a role in regulating myogenic tone as well as the myogenic response and are known to be involved in the mechanism of action of vasoconstrictors (refer to reviews by Beech, 2005, 2013) and will be discussed in the section II.B.4. However, for details on the role of TRP channels in vascular function and how the dysregulation of vascular as well as endothelial TRP channels is related to vascular-related pathologies, please see the recent review by Earley and Brayden (2015).

#### 2. Ca^2+^ Sparks.

Highly restricted and large Ca^2+^ release events through SR RyRs are called Ca^2+^ sparks, and Ca^2+^ sparks have an important regulatory role in VSMCs. Similar to sparklets, their spatial reach is small, so they have no direct effect on contractility; however, the proximity of RyRs to the plasma membrane allows them to affect global [Ca^2+^]_i_ indirectly (reviewed by Amberg and Navedo, 2013). The nature of the VSMC’s response to Ca^2+^ sparks depends on the Ca^2+^-activated plasmalemmal ion channels that are spatially coupled to the RyRs. In many VSMC, tissues sparks are targeted to large conductance Ca^2+^-dependent K^+^ channels (BK) that oppose vasoconstriction by allowing hyperpolarizing outward K^+^ currents (Nelson et al., 1995). On the other hand, Ca^2+^ gated Cl^−^ channels (CaCCs) depolarize the plasmalemma and thereby enhance Ca^2+^ influx through LTCCs (Kitamura and Yamazaki, 2001; Leblanc et al., 2005).

##### a. Ca^2+^-dependent K^+^ channel-coupled sparks.

A single Ca^2+^ spark increases the open probability of about 30 BK channels in its proximity by 100-fold (Jaggar et al., 2000; Perez et al., 2001). Sparks can occur spontaneously or be triggered by TRPV4 sparklets in the form of a CICR mechanism. Structurally, plasmalemmal BK channels in VSMCs are formed by four pore forming alpha subunits encoded by the slo gene and regulatory *β*1 subunits that are not necessary for the formation of a functional channel (Toro et al., 1998). However, the *β*1 subunits play a significant role in modulating the Ca^2+^ sensitivity and hence functional coupling to RyRs (Brenner et al., 2000). It has been demonstrated by several groups that ablation of the *β*1 subunit in mice leads to desensitization to Ca^2+^ and functional uncoupling of BK channels from Ca^2+^ sparks, causing membrane depolarization, increases in arterial tone, and eventually hypertension (Brenner et al., 2000; Pluger et al., 2000). Furthermore for ATII-induced hypertension, it has been reported that the *β*1, but not the pore-forming alpha subunit, is downregulated, which mediates a decrease in the sensitivity of BK channels and thereby contributes to vascular dysfunction (Amberg et al., 2003; Nieves-Cintron et al., 2007). Consistent with these results, associations between gain of function mutations of the *β*1 subunit and a lower prevalence of diastolic hypertension have been described (Fernandez-Fernandez et al., 2004; Nelson and Bonev, 2004; Senti et al., 2005). Additionally, it has been demonstrated that *β*1 subunit downregulation in ATII-induced hypertension is mediated by enhanced activity of the transcription factor NFATc3 (Amberg et al., 2004; Nieves-Cintron et al., 2007). However in hypertensive animals, there have also been studies that have found higher expression of the *α* subunit in VSMCs, suggesting that the BK channel is primarily involved in a compensatory response to increased VSMC tone from enhanced LTCC or decreased K_v_ activity (reviewed in Cox and Rusch, 2002). Hence BK channels appear to be involved in the pathogenesis in some as well as compensation and protection in other forms of hypertension.

Using different strategies to modulate plasmalemmal K^+^ channel activity to inhibit *β*1 downregulation in developing hypertension or to increase *β*1 expression in VSMCs would appear to be a promising approach for the treatment of hypertension. In addition, a number of BK channel openers are currently in development (Webb et al., 2015); however, the use of BK channel openers for the treatment of hypertension is limited by concerns for off-site effects in other smooth muscle tissues. As the *β*1 subunit of the BK channel seems to be expressed predominantly in VSMCs (Tanaka et al., 1997), targeting *β*1 expression through gene therapy or modulation of the NFATc3 pathway represents a possible alternative (reviewed by Joseph et al., 2013).

##### b. Ca^2+^ gated Cl^−^ channel-coupled sparks.

In some VSMCs, Ca^2+^ sparks are coupled to CaCCs, and their activation is followed by “spontaneous transient inward currents” or STICs. The two families of CaCCs that have only recently been identified are called bestrophins and TMEM16A. They are also expressed in renal tubular epithelium as well as the heart and are hence thought to have a multidimensional role in blood pressure regulation (reviewed by Matchkov et al., 2015). Because the activation of these channels results in plasma membrane depolarizing currents, they are thought to have an amplifying effect on vascular contractile stimuli by indirectly causing the opening of LTCCs (Leblanc et al., 2005; Matchkov et al., 2013; Bulley and Jaggar, 2014). Indeed, downregulation or inhibition of TMEM16A led to decreased arterial constriction in a variety of studies (Jensen and Skott, 1996; Bulley et al., 2012; Davis et al., 2013; Dam et al., 2014), and a smooth muscle KO of TMEM16A in mice lead to a decrease in the ability of ATII infusion to produce hypertension. As CaCCs are also permeable to other anions such as HCO_3_^−^, it is also possible that some effect may be due to changes in intracellular pH that would affect pH-sensitive enzymes including Rho kinase (Boedtkjer et al., 2011). Although there are a number of substances that can inhibit CaCC activity in vitro, the unknown molecular identity of TMEM16A as well as its expression in various tissues would suggest that there is poor pharmacological specificity in vivo and there would be many off target effects (Greenwood and Leblanc, 2007; Boedtkjer et al., 2008).

#### 3. Ca^2+^ Waves.

Activation of CaCCs is also often mediated by Ca^2+^ waves, a Ca^2+^ signal in which subsequent openings of IP_3_Rs and in some tissues RyRs on the SR cause a wave of Ca^2+^ release events across the entire length of the VSMC, usually close to the plasma membrane (Iino et al., 1994; Hill-Eubanks et al., 2011; Amberg and Navedo, 2013). Westcott and colleagues described the contrasting roles of RyRs and IP_3_Rs for the effects of Ca^2+^ waves in arterioles and upstream feed arteries. Although arteriolar Ca^2+^ waves are solely IP_3_R mediated and therefore not inhibited by ryanodine, RyR inhibitors decreased Ca^2+^ waves in feed arteries. In both tissues, Ca^2+^ waves were inhibited with phospholipase C (PLC) inhibitors and IP_3_R blockers, which led to a decrease in [Ca^2+^_i_] and vasodilation. Therefore, IP_3_Rs contribute to Ca^2+^ waves in both tissues as part of a positive feedback loop for myogenic tone. In contrast, despite the inhibition of Ca^2+^ sparks and waves in feed arteries, inhibition of RyRs caused an increase in [Ca^2+^_i_] and led to vasoconstriction. This was abolished in the presence of BK-channel blocker paxilline, which supports the hypothesis that RyRs, which are involved in Ca^2+^ waves, are also coupled to BK channels and part of a negative feedback regulation of myogenic tone (Lamont et al., 2003; Westcott and Jackson, 2011; Stewart et al., 2012; Westcott et al., 2012).

In arterioles, Ca^2+^ waves are initiated by IP_3_-dependent opening of an IP_3_R creating a Ca^2+^ “blip,” a single IP_3_R opening (Bootman and Berridge, 1996), or a “puff,” short Ca^2+^ release from a small cluster of IP_3_Rs that is biophysically different from a RyR-mediated spark (Bootman and Berridge, 1996; Thomas et al., 1998). Subsequently, clusters of IP_3_Rs open in response to the Ca^2+^ released by adjacent IP_3_Rs (CICR) and are inactivated as the [Ca^2+^] rises further. The IP_3_R’s Ca^2+^-dependent activation/inactivation properties are reflected in the wave-like pattern of IP_3_R-mediated Ca^2+^ release (Foskett et al., 2007). Ca^2+^ wave initiation depends on IP_3_ synthesis by PLC, which occurs after activation of G-protein-coupled receptors by their respective agonists, including norepinephrine and endothelin-1 (Lamont et al., 2003; Westcott and Jackson, 2011; Stewart et al., 2012; Westcott et al., 2012). However, Ca^2+^ waves are also seen in the absence of agonists and depend on the spontaneous basal production of IP_3_ by PLC, which varies in different vascular beds, and thus will affect the frequency of Ca^2+^ release through RyRs via CICR (Gordienko and Bolton, 2002). In arterioles, the wave leads to VSMC contraction by directly increasing [Ca^2+^]_I_, and this effect is amplified by the Ca^2+^-dependent opening of CaCCs in the plasma membrane that leads to membrane depolarization and increased Ca^2+^ influx through LTCCs.

#### 4. Store-Operated Calcium Entry.

When SR Ca^2+^ stores are depleted after release through IP_3_Rs, the SR Ca^2+^ sensor STIM (stromal interaction molecule) relocates to the SR-plasmalemmal junction and physically interacts with and activates the selective Ca^2+^ channel Orai [CRAC (calcium release activated calcium channel); reviewed by Trebak, 2012]. For VSMCs in the normal physiologic contractile phenotype, the expression of STIM/Orai is relatively low, but its expression is upregulated when the VSMC changes its phenotype to the proliferative state (Potier et al., 2009). In a rodent STIM/Orai knockdown model, nuclear factor activated T-cells (NFAT) translocation to the nucleus was decreased and VSMC proliferation in response to vascular injury was impaired (Aubart et al., 2009; Guo et al., 2009; Zhang et al., 2011). In spontaneously hypertensive rats, STIM/Orai is upregulated and depletion of SR stores lead to greater SOCE, which may represent an independent mechanism leading to increased VSMC [Ca^2+^]_i_ in hypertension (Giachini et al., 2009b). Furthermore, these investigators found evidence suggesting that increased STIM/Orai activity may underlie sex differences in the development of hypertension. They determined that inactivation of the STIM/Orai mechanism with CRAC inhibitors as well as antibodies to STIM or Orai during and after store depletion abolished sex differences in spontaneous contractions of VSMCs (Giachini et al., 2009a). Thus CRACs represent a novel target in the treatment of hypertension.

However, there are a number of studies also suggesting a role of TRPC channels in SOCE (reviewed by Beech, 2012). Both TRPCs and Orai channels can be activated by STIM after store depletion (Zeng et al., 2008; Park et al., 2009); however, their individual contribution to SOCE is variable. Studies have demonstrated a partial suppression of SOCE by Orai and TRPC siRNA, respectively (Li et al., 2008). The nature of TRPC-Orai interaction, or if there is in fact one, is currently unresolved (refer to Earley and Brayden, 2015), but both channels can also be activated independently from store depletion or Ca^2+^ release and their downstream effects on activation differ. TRPs exhibit multiplicity of gating and hence have been suggested to integrate various cellular signals including store depletion (Albert and Large, 2002). TRPC1 mediates Ca^2+^ influx after store depletion with thapsigargin (Xu and Beech, 2001; Sweeney et al., 2002; Lin et al., 2004) and is thought to be involved in contractile modulation and regulation of cell proliferation; however, more data are needed to determine its exact function. TRPC6 is a channel that mediates cation movement in a variety of experimental settings. In some tissues, inhibition of TRPC6 leads to a decrease in SOCE, but it also appears to be involved in SR-independent signaling. Studies demonstrated that TRPC6 is store and receptor operated, as well as stretch and osmotically activated in VSMCs. It can associate and form heteromultimers with TRPC3, which leads to tonic channel activation (Dietrich et al., 2003). TRPC3 and TRPC6 are upregulated in idiopathic pulmonary hypertension and an siRNA-induced decrease of TRPC6 expression decreases proliferation of cultured pulmonary artery VSMC isolated from patients with pulmonary hypertension. Furthermore, chronic hypoxia increases TRPC6 expression, whereas the ET-1 antagonist bosentan, a common treatment of PAH, lowers TRPC6 expression in pulmonary VSMCs (Kunichika et al., 2004; Lin et al., 2004). These are merely examples of the various roles TRPC channels occupy in VSMC signaling and a complete discussion of TRPC channels in health and disease has been recently presented in a number of reviews (Beech, 2005, 2012; Earley and Brayden, 2015).

### C. Excitation-Transcription Coupling

An important mode in which Ca^2+^ can regulate VSMC contractility is by regulating the composition of the contractile apparatus, ion channels, and cellular signaling molecules by influencing VSMC gene transcription (reviewed by Kudryavtseva et al., 2013). In certain cytoplasmic locations, high [Ca^2+^] activates specific kinases or phosphatases that in turn lead to activation and translocation of transcription factors to the nucleus. In the nucleus, Ca^2+^ can bind helix-loop-helix-loop structural domain or motif (EF hand) containing transcription factors directly (Carrion et al., 1999) or modulate transcription factor binding via Ca^2+^/CaM-S100 complexes (Hermann et al., 1998). Although in many disease states VSMCs can completely lose their contractile function due to a phenotype switch toward a proliferative ECM-producing phenotype, more subtle changes within the contractile phenotype are also thought to play a role in the increased VSMC contractility observed in hypertension.

NFAT is a major target of calcineurin, and it translocates to the nucleus upon calcineurin-mediated dephosphorylation. Calcineurin activation is enhanced by the activity of the AKAP150-bound LTCC signaling pentad (Oliveria et al., 2007; Nieves-Cintron et al., 2008). Hence, NFAT activity is regulated by the level of persistent sparklet activity, but is also dependent on simultaneous inhibition of its nuclear export (Gomez et al., 2003). Interestingly, although membrane-depolarizing signals such as IP_3_R-mediated Ca^2+^ waves are thought to cause an increase in NFATc3 activation via enhanced LTCC activity, RyR-mediated Ca^2+^ release from the SR decreases NFAT activity by an LTCC independent mechanism (Gomez et al., 2002). SOCE has also been implicated in NFAT activation, and its disruption led to reduced hypoxia-induced NFAT nuclear translocation in pulmonary VSMCs (Bierer et al., 2011; Hou et al., 2013). Although a variety of Ca^2+^ signals lead to NFAT activation, persistently raised levels of intracellular Ca^2+^ fail to induce NFAT (Stevenson et al., 2001; Gonzalez Bosc et al., 2004). Therefore it is thought that oscillating Ca^2+^ signals (Matchkov et al., 2012) and concomitant inhibition of nuclear export (Gomez et al., 2003) leads to nuclear NFAT accumulation. It is well documented that inhibition of the calcineurin/NFAT pathway reduces VSMC proliferation, neointima formation, and vascular remodeling in response to injury (Liu et al., 2005; Nilsson et al., 2008; Esteban et al., 2011; Hou et al., 2013). However there are also studies indicating a role for NFAT within the contractile phenotype, altering the expression of plasmalemmal ion channels including BK (Nieves-Cintron et al., 2007) and K_v_ channels (Amberg et al., 2004) and thereby increasing VSMC contractility and ultimately arterial tone.

In contrast to NFAT, CREB is regulated by the Ca^2+^ dependent kinases CaMKII and CaMKIV (Cartin et al., 2000). Ca^2+^ influx through LTCCs is important for activated phospho-CREB to accumulate in the nucleus (Stevenson et al., 2001). Signals that increase LTCC activity including IP_3_R-mediated Ca^2+^ waves (Barlow et al., 2006) and SOCE (Pulver et al., 2004; Takahashi et al., 2007) lead to increased CREB-induced transcription, whereas Ca^2+^ sparks counteract CREB activity by hyperpolarizing the plasmalemma and reducing LTCC flux (Cartin et al., 2000; Wellman and Nelson, 2003). Because CREB activates genes involved in the contractile, as well as the proliferative phenotype, the ultimate effect of CREB activation on VSMC phenotype has not yet been determined. However in contrast to NFAT, CREB is induced by any signal that causes a sustained increase in Ca^2+^ entry through LTCCs.

In addition to controlling transcription indirectly through CREB, LTCCs have also been found to directly influence gene expression in VSMCs. In a study by Bannister et al. (2013) it was determined that when the C-terminal end of the LTCC (CCt) is cleaved, it either reassociates with LTCCs and reduces LTCC sparklet activity or it relocates to the nucleus and inhibits the transcription of LTCCs. The CCt thus acts as a bimodal vasodilator by decreasing LTCC transcription and reducing voltage-dependent LTCC opening. However, the enzyme responsible for CCt cleavage and the mechanism(s) for regulation have yet to be determined. Potentiating the effects of CCt through increased cleavage or possibly stimulation of CCt promoter sequences may offer another novel approach to controlling vascular contractility.

### D. Conclusion

There are a variety of Ca^2+^-mediated mechanisms that increase VSMC contractility and are possible targets in antihypertensive therapy, some of which are well understood and can be specifically inhibited in vitro. However the development of novel treatments is often limited by the expression of the targets in nonvascular smooth muscle tissues and thus the many off site effects. A possible solution to this issue could be targeting specific therapies to VSMCs using viral vectors. There are a number of successful proof of concept studies using this technique that were recently reviewed by Joseph et al. (2013). Another issue that limits progress in the effort to find novel pharmacologic therapies in Ca^2+^ signaling is that the composition of Ca^2+^ signaling microdomains differs in various vascular beds, and hence results cannot always be generalized for VSMCs. This problem again highlights the importance of genetic KO and knockdown studies as a tool to explore targets for gene therapy for the treatment of hypertension.

## III. Vascular Smooth Muscle Signal Transduction

### A. Signaling Pathways—Overview

Many potential therapeutic strategies are designed to activate or inhibit specific signaling pathways in the vascular smooth muscle cell. It is clear that multiple vascular signaling pathways coexist as spatially separate signaling compartments in individual differentiated vascular smooth muscle (dVSM) cells and coordinated by a multitude of scaffolding proteins. However, these pathways are often overlapping, multilayered, and tissue specific. The tissue-specific nature of these pathways, even between different vessels or sizes of vessels, has led to much controversy on the relative importance of one pathway versus another. Ultimately, however, the possibility of multiple pathways that could be activated or inhibited in various disease states or as functional compensation to physiologic stress gives the system considerable functional plasticity.

**Fig. 3.**
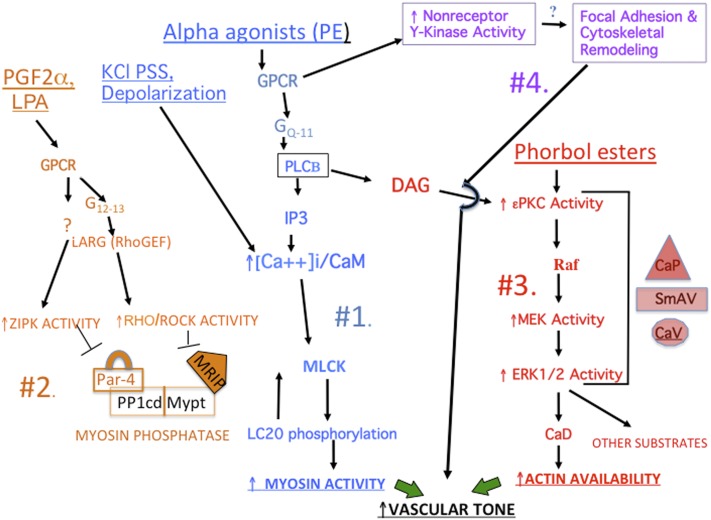
Overview of pathways regulating vascular tone. See text for details. For additional detailed pathways, see subsequent figures.

At the simplest level, it is well established that vascular tone can be increased either by increasing activation of myosin (**Pathways #1 & #2**, Fig. 3) or, in a manner analogous to that in striated muscle, by removal of inhibition of actin (**Pathway #3**, Fig. 3). Either mechanism will lead to an increase in actomyosin activation and crossbridge cycling. Recently, several laboratories have reported more controversial mechanisms by which agonists or biomechanical forces can regulate both vascular and airway smooth muscle contractility by remodeling cytoskeletal attachments (Walsh and Cole, 2013; Zhang et al., 2015) (**Pathway #4**, Fig. 3). These four pathways are discussed in more detail below.

#### 1. Major Pathways Leading to Changes in the Activity of Smooth Muscle Myosin.

This has been a very active area of investigation by vascular smooth muscle biologists and, as discussed below, has already identified many potential pharmacologic target molecules and in some cases led to possible drug candidates.

Smooth muscle myosin differs from skeletal and cardiac myosins in that it lacks intrinsic myosin ATPase activity in the pure state. Smooth muscle myosin requires a posttranslational modification, phosphorylation of Ser 19 of the 20-kDa regulatory light chain to display enzymatic activity. This phosphorylation is caused by a dedicated Ser/Thr kinase, myosin light chain kinase (MLCK). (Ito and Hartshorne, 1990)

MLCK is a Ca/CaM-dependent kinase and is most simply activated by increases in cytoplasmic ionized Ca ([Ca^2+^_i_]) levels (**Pathway #1**, Fig. 3) such as occurs with a large number of G-protein coupled receptor-mediated agonists, such as alpha agonists or by depolarization of the cell membrane by channel activity or experimentally by equimolar replacement of NaCl with KCl in physiologic saline solution. It has also been reported that increases in the free CaM level (Hulvershorn et al., 2001) or Ca-independent changes in the kinase activity of MLCK can also occur (Kim et al., 2000) by phosphorylation-mediated events.

Dephosphorylation of myosin by myosin phosphatase (MP) decreases its activity, and conversely, inhibition of MP will increase its activity. A large number of pathways, such as those activated by PGF2a and lysophosphatidic acid (**Pathway #2**, Fig. 3), have been reported to inhibit MP through either Rho-associated protein kinase (ROCK)-dependent mechanisms or those involving Zipper-interacting protein kinase. These pathways are discussed in detail in section IV.

CaMKinase II is another Ca/CaM-dependent kinase with the interesting property, when activated, of autophosphorylating itself on T287, which leads to a sustained activity after Ca is removed, giving it a chemical “memory” of having been activated (Hudmon and Schulman, 2002; Lisman et al., 2002). Conversely, when S26 in the catalytic domain is autophosphorylated, it can terminate sustained kinase activity, making it “forget” prior activation (Yilmaz et al., 2013).

There are four main isoforms of CaMKII, the alpha, beta, gamma, and delta isoforms. The gamma (especially the G-2 variant) (Kim et al., 2000; Marganski et al., 2005) and delta (especially the d2 variant) (Ginnan et al., 2012) isoforms have been shown to play important roles in smooth muscle, with the gamma isoforms primarily regulating contractility and the delta isoforms regulating proliferation. To a large degree the gamma/delta ratio represents the degree of a phenotype switch between the contractile/proliferative phenotypes displayed by smooth muscle in different settings. Vascular injury reduces gamma isoform expression and upregulates delta expression (Singer, 2012). Conversely, siRNA-mediated knock down of the delta isoform attenuates VSM proliferation and neointimal formation. The conditional smooth muscle knockout of CaMKIIdelta significantly delays the progression of airway smooth muscle hyperresponsiveness to an ovalbumin challenge and this isoform is upregulated in the wild-type mouse in response to the same challenge. Thus the delta isoform may play a role in smooth muscle inflammatory responses (Spinelli et al., 2015).

With respect to the gamma isoform and its regulation of smooth muscle contractility, six smooth variants of the gamma isoform of CaMKII have been described with varying kinetics of Ca/CaM-dependent activation/deactivation (Gangopadhyay et al., 2003). One variant, the G-2 variant, which has unique sequence in the association domain of the kinase (Gangopadhyay et al., 2003), has been shown to have scaffolding properties with respect to ERK (extracellular regulated kinase). Antisense specific for the G-2 variant (Marganski et al., 2005) or generic against the gamma isoform or small molecule inhibitor studies (Kim et al., 2000; Rokolya and Singer, 2000) have all demonstrated roles for gamma CaMKII in the regulation of contractility. The CaMKII gamma G-2 variant is reported to be associated with vimentin intermediate filaments and dense bodies in unstimulated vascular smooth muscle cells and on activation by depolarization-mediated increases in cytosolic Ca^2+^ levels the G-2 variant translocates to the cortical focal adhesions (Marganski et al., 2005; Gangopadhyay et al., 2008). This variant also has been shown to be specifically dephosphorylated by an SCP3 phosphatase. SCP3 is a PP2C typed protein phosphatase, primarily expressed in vascular tissues and specifically binds to the unique association domain sequence in CaMKII gamma G-2. G-2 is bound to this phosphatase in unstimulated vascular smooth muscle tissue but is released upon depolarization-mediated Ca^2+^ influx. This phosphatase does not appear to regulate kinase activity but rather is thought to result in the exposure of a SH3 domain targeting of the kinase, which leads to targeting to focal adhesions (Gangopadhyay et al., 2008).

Although CaMKIIgamma is known to be activated by stimuli that increase the free Ca^2+^ level in dVSM, and antisense or inhibitors to CaMKII decrease the amplitude of the contraction to KCl PSS, the exact pathways that regulate contractility are still being confirmed. It has been shown that knock down of the gamma isoform or the G-2 variant specifically, as well as small molecule inhibitors of the kinase, lead to an inhibition of ERK activation and an inhibition of MLCK (Kim et al., 2000; Rokolya and Singer, 2000; Marganski et al., 2005). ERK has been shown to be capable of activating MLCK in other systems (Morrison et al., 1996; Nguyen et al., 1999), but whether this is the link in smooth muscle has not been definitively shown. Additionally in cultured vascular cells, CaMKII is rapidly activated after upon adherence of the cells upon plating onto ECM or poly-lysine. Adherence led to CaMKII-dependent tyrosine phosphorylation of paxillin and ERK activation (Lu et al., 2005). The CaMKII delta 2 variant has also been shown to regulate vascular smooth muscle cell motility in culture through a Src-family tyrosine kinase, Fyn (Ginnan et al., 2013). Because focal adhesions are known to serve as ERK scaffolds in contractile smooth muscle, this is an appealing possible link. Thus, at the present time, although CaMKII is clearly an important regulator of Ca^2+^-dependent vascular contractility, the complete molecular details of the CaMKII pathway used by contractile vascular smooth muscle to regulate contractility are not yet resolved. It is clear, however, that these details represent considerable untapped potential as future therapeutic targets for the modulation of Ca^2+^-dependent vascular contractility and hence blood pressure. Additionally, the wealth of information on isoform specific effects of CaMKII, especially the gamma G-2 variant, offers the potential of considerable tissue and smooth muscle phenotype specificity of such therapeutics.

#### 2. Pathways Leading to Changes in Actin Availability for Interaction with Myosin.

In contrast to the pathways described above, ex vivo studies (Walsh et al., 1994; Horowitz et al., 1996a; Dessy et al., 1998) have demonstrated that phorbol esters, or alpha agonists, by activating PKC can trigger increases in contractile force that in some tissues are either Ca^2+^ independent or cause leftward shifts in the [Ca^2+^_i_]-force relationship. The Ca^2+^ dependence of phorbol ester contractions varies in different smooth muscle tissues, dependent on the isoforms of PKC present in those tissues. The alpha, beta, and gamma isoforms are calcium dependent, but delta and epsilon are calcium independent. Thus, phorbol ester and alpha agonist-induced contractions have been described as being Ca^2+^ independent in experiments using the aorta of the ferret, which contains significant amounts of the epsilon isoform of PKC, but tissues containing more PKC alpha, such as the portal vein of the ferret, show an *increased Ca^2+^ sensitivity* but are still Ca^2+^ sensitive (Lee et al., 1999) and lead to the activation of **Pathway #3** in Fig. 3.

**Pathway 3** can be activated by diacylglycerol (DAG) release, generated by the activation of GPCRs in vascular smooth muscle, or by myometrial stretch, RU486, and labor in the rat and human myometrium (Li et al., 2003, 2004; Morgan, 2014). Interestingly, in the presence of extracellular Ca^2+^ (Ca_e_), phenylephrine (PE) will activate pathways 1, 3, and 4 but in the absence of Ca_e_ and in the absence of changes in 20kda light chain phosphorylation, a contraction persists in aorta of the ferret (Dessy et al., 1998). Phorbol esters give a maximal tonic contraction in the absence of changes in 20kda light chain phosphorylation, even in the presence of Ca_e_ in this system (Jiang and Morgan, 1989).

When activated, **Pathway #3** leads, indirectly, to the PKC-dependent activation of MEK, a dual activity kinase that phosphorylates ERK on a tyrosine and threonine, resulting in activation of ERK. ERK activation can have multiple downstream effects, largely controlled by output-specific scaffolding proteins (see below). In contractile smooth muscle, these downstream effects include phosphorylation of the actin binding protein caldesmon. Caldesmon has been described as being functionally analogous to the troponin complex in striated muscle in that it blocks the access of myosin to actin and hence impairs crossbridge cycling. The C-terminal end of caldesmon is responsible for the direct inhibition of myosin ATPase activity (Sobue et al., 1982; Bryan et al., 1990; Wang et al., 1991). Investigators have demonstrated that the interaction of the actin-binding domain of caldesmon with actin is responsible for the inhibition the actomyosin ATPase (AMATPase) (Velaz et al., 1990) by decreasing the rate of Pi release by 80% (Alahyan et al., 2006).

Caldesmon has an NH_2_-terminal myosin-binding domain, in addition to the COOH-terminal actin-binding domain, and thus, in theory, could crosslink actin and myosin (Goncharova et al., 2001). However, Lee et al. (2000b) also observed a tethering effect of the N-terminal region of caldesmon to myosin that has the proposed agonist-dependent functional effect of positioning caldesmon so that its C-terminal end no longer inhibits myosin activity. The binding of caldesmon to myosin is regulated by Ca^2+^-calmodulin, whereas the interaction with actin is regulated by ERK phosphorylation at Ser789 on caldesmon (Hemric et al., 1993; Patchell et al., 2002).

In general, in most systems it appears that phosphorylation of caldesmon on Ser789 by ERK, PAK, or other serine kinases can reverse caldesmon-mediated inhibition of myosin ATPase activity (Childs et al., 1992; Foster et al., 2000; Kim et al., 2008a) (**Pathway #3**, Fig. 3). However, results from mechanical experiments examining caldesmon function are variable. In smooth muscle from caldesmon KO mice, compared with WT controls, both the rate of force activation and the steady-state force in response to depolarization, phorbol esters, and carbachol were similar, but the rate of force relaxation was reduced (Guo et al., 2013). In contrast to these results, an siRNA-induced decrease in caldesmon expression lowered both isometric force and muscle shortening velocity (Smolock et al., 2009).

In cultured smooth muscle cells, p42/44 MAPK has been clearly demonstrated to phosphorylate caldesmon at Ser789 (Hedges et al., 2000), but for agonist activation of intact smooth muscle, the kinase responsible for caldesmon phosphorylation remains a matter of controversy or may involve different kinases in different settings (Wang, 2008). In skinned smooth muscle strips, ERK-induced phosphorylation of caldesmon did not alter the force-Ca^2+^ relationship (Nixon et al., 1995). Porcine carotid artery preparations did not display detectable phosphorylation of caldesmon at the ERK sites during phorbol ester stimulation, (D'Angelo et al., 1999), but PAK phosphorylation at Thr627, Ser631, Ser635, and Ser642 was demonstrated to reduce caldesmon’s inhibition of the AMATPase (Hamden et al., 2010). On the other hand, ERK-mediated phosphorylation of caldesmon at 789 has been clearly shown in ferret aorta preparations as well as mouse aorta and rat myometrium. Furthermore, although an increase in caldesmon phosphorylation was observed by Katoch and Moreland (1995) in porcine carotid artery during both depolarization and histamine stimulation, experiments using inhibitors suggested that a second kinase in addition to ERK also phosphorylates caldesmon (Gorenne et al., 2004).

In contrast to the myosin regulatory pathways, this is a relatively untapped area of investigation for the discovery of new target molecules with therapeutic potential. The relative importance of these pathways are definitely tissue and species specific. Interestingly, the strongest evidence of the importance of these pathways appears to have come from myometrial smooth muscle in the setting of preterm labor (Li et al., 2003, 2004, 2007, 2009). Thus, the potential is there for novel and possibly quite specific therapeutic targets within these pathways.

#### 3. Tyrosine Phosphorylation of Smooth Muscle Proteins.

The vast majority of known protein phosphorylation events in the contractile, differentiated smooth muscle cell are serine/threonine events. Where phosphotyrosine screening with immunoblots of contractile vascular as well as myometrial (Li et al., 2007, 2009; Min et al., 2012) smooth muscle tissue has been performed, the reactive bands have been almost exclusively focal adhesion-associated proteins. These tyrosine phosphorylations are largely sensitive to Src inhibitors, pointing to the presence of focal adhesion remodeling in nonproliferating, nonmigrating smooth muscle (Poythress et al., 2013; Ohanian et al., 2014; Zhang et al., 2015). These mechanisms have been especially studied in vascular and airway smooth muscles, resulting in pathways extending from **Pathway #4 (**Fig. 3). These mechanisms will be discussed in further detail in section V below.

#### 4. Calcium Sensitization of the Contractile Apparatus.

When our group first (Bradley and Morgan, 1982, 1985) measured intracellular Ca levels ([Ca^2+^]_i_) in dVSM with the photoprotein aequorin, we noticed that agonists often cause tonic contractions with only transient increases in [Ca^2+^]_i_ or differing magnitudes of [Ca^2+^]_i_, reflecting apparent changes in “Ca^2+^ sensitivity” of the contractile apparatus (Bradley and Morgan, 1985). This dissociation between [Ca^2+^]_i_ and force has been confirmed with many agonists and many different Ca^2+^ indicators in contractile smooth muscle tissues and with permeabilized smooth muscle preparations where leftward shifts in the Ca^2+^-force relationship in response to agonists and various agents are seen (Ruegg and Pfitzer, 1985; Somlyo et al., 1999). Mechanistically, we now have molecular explanations for this phenomenology. Changes in the apparent Ca^2+^ sensitivity of the contractile apparatus have been partially explained by the ability of agonists to regulate the activity of myosin phosphatase (MP) (Somlyo and Somlyo, 2003) (**Pathway #2**, Fig. 3), partially by the ability of ERK to regulate the action of caldesmon (CaD) to inhibit acto-myosin interactions (Kordowska et al., 2006) **(Pathway #3**, Fig. 3) and clearly also by yet to be defined pathways.

### B. Subcellular Spatial Organization of Signaling Pathways

The complexity of signaling pathways in the smooth muscle cell raises the issue of how kinases connect with their complex specific upstream activators and downstream substrates in an agonist-specific manner within the three-dimensional space of the interior of a cell. Scaffold proteins are now recognized to play important roles in coordinating mammalian signal transduction (Morrison and Davis, 2003; Kolch, 2005). Protein scaffolds are defined as *docking platforms* that colocalize components of kinase cascades and facilitate activation of the kinases (McKay and Morrison, 2007). The scaffolds themselves generally lack enzymatic activity but promote specific outcomes of the pathway. Protein scaffolds can be thought of as “traffic cops” in what would otherwise be the chaos of multiple competing intracellular signaling pathways. Because scaffold proteins add specificity to the cellular pathways, they also present very attractive targets for drug discovery programs. Two major types of scaffolds relevant for the smooth muscle cell are ERK scaffolds and scaffolds for regulators of myosin phosphatase.

#### 1. Extracellular Regulated Kinase Scaffolds (Calponin, SmAV, Paxillin, Caveolin, FAK, IQGAP).

ERK is known to often be targeted to the intranuclear space in proliferative cells and to regulate nuclear signaling, especially to transcription factors (Dhanasekaran et al., 2007). In the smooth muscle cell these pathways can lead to a proliferative phenotype for the smooth muscle cell. These pathways will not be discussed here, but rather we will focus on those most relevant for the fully differentiated contractile cell. Even so, much of this work has been performed using cell culture models and no doubt needs further work in specific contractile smooth muscle tissue systems.

Calponin is a bit of an enigma and its function in smooth muscle is still debated. It has been reported to serve both cytoskeletal and signaling functions (Winder and Walsh, 1990; Birukov et al., 1991; Menice et al., 1997; Leinweber et al., 1999a,b, 2000; Appel et al., 2010). Both PKC and CAM kinase II phosphorylate calponin at Ser175 (Winder and Walsh, 1990), and after phosphorylation, calponin loses its ability both to bind actin and inhibit the AMATPase (Winder et al., 1993). Calponin has been reported directly to regulate contractility (el-Mezgueldi and Marston, 1996; Obara et al., 1996; Winder et al., 1998; Takahashi et al., 2000; Je et al., 2001; Szymanski et al., 2003) but others have reported negative results (Matthew et al., 2000). Calponin phosphorylation increases during carbachol stimulation of smooth muscle (Winder et al., 1993). Consistent with a physiologic role for calponin in the regulation of contractility are results in skinned smooth muscle; the addition of exogenous calponin reduces both Ca^2+^ activated force (Horowitz et al., 1996b; Obara et al., 1996) and maximal shortening velocity (Jaworowski et al., 1995). In the smooth muscle isolated from calponin KO mice, compared with WT controls, muscle-shortening velocity is significantly higher, but there is no difference in the force produced by Ca^2+^, carbachol, or phorbol esters (Matthew et al., 2000). However, the addition of exogenous calponin reduces force in skinned single smooth muscle cells, and the Ser175Ala calponin mutant has no effect on force (Horowitz et al., 1996a). In intact smooth muscle during agonist-induced activation, calponin redistributes from the contractile filaments to the cell surface, which is attenuated with the inhibition of PKC (Parker et al., 1994, 1998; Gallant et al., 2011). Thus, these results are consistent with a role for calponin in the regulation of smooth muscle contraction; agonist stimulation leads to the activation of PKC, which phosphorylates calponin at Ser-175 to decrease calponin’s interaction with actin to relieve calponin’s inhibition of the AMATPase.

Three isoforms exist for calponin. h1CaP/CNN1/basic calponin is one of the most specific and rigorous markers for the differentiated smooth muscle phenotype. h2CaP/CNN2/neutral calponin and h3/acidic CaP/CNN3/aCaP are more widely distributed but appear to also be expressed in some smooth muscles (Takahashi et al., 1988; Strasser et al., 1993; Applegate et al., 1994). Work in our group has led us to propose that calponin is an adaptor protein for ERK (Leinweber et al., 1999a,b; Appel et al., 2010). Antisense knock down of calponin (Je et al., 2001) led to decreased ERK activity and contractile force after alpha agonist activation but not after a depolarizing stimulus. Also, protein chemistry studies and cellular immunoprecipitation studies demonstrated that CaP directly binds both PKC and ERK and in intact vascular smooth muscle cells (Leinweber et al., 2000) and is bound to the thin filaments but translocates to the cortex of the cell in response to alpha agonists (Parker et al., 1998). A detailed model has been suggested where agonists activate PKC, which phosphorylates CaP, releasing it from the thin filaments (Kim et al., 2008a). Colocalization of ERK and CaP is seen in unstimulated vascular smooth muscle cells and agonist-activation leads to the translocation of a PKC/CaP/ERK complex to the cell cortex, likely meeting up with SmaV (see below), Raf, and MEK, which leads to the activation of ERK, at which point it is seen to return to the contractile filaments and CaD phosphorylation of the ERK sites is observed (Khalil and Morgan, 1993).

SmAV is the smooth muscle isoform of a major scaffolding protein supervillin (Pestonjamasp et al., 1997). SmAV was initially cloned and identified SmAV as a CaP binding partner in a two-hybrid assay with CaP as bait (Gangopadhyay et al., 2004), and it was found that SmAV acts as an ERK scaffold, leading to the regulation of CaD phosphorylation (Gangopadhyay et al., 2009). Data have been published indicating that CaP (Menice et al., 1997), SmAV (Gangopadhyay et al., 2004) and CaV (Je et al., 2001) function as scaffolds coordinating **Pathway #3**.

Paxillin, better known as a focal adhesion protein, is also known to bind the classic ERK “signaling module” of Raf, MEK, and ERK (McKay and Morrison, 2007). In quiescent cultured cells, paxillin is constitutively associated with MEK, but Ishibe et al. (2003) showed that when cells are stimulated with HGF, Src-mediated phosphorylation of paxillin at Y118 leads to the recruitment of ERK, followed by Raf, which leads to ERK phosphorylation and activation. Shortly thereafter focal adhesion kinase (FAK) is recruited to the complex, leading to FA remodeling in both cultured cell systems and airway smooth muscle (Zhang et al., 2015). Thus, paxillin provides a signaling hub in the vicinity of focal adhesions that can have specific cytoskeletal outcomes.

Caveolin is an extensively studied protein but there are still many mysteries regarding its function. A caveolin-associated protein has also discovered and named cavin (Liu and Pilch, 2008; Ding et al., 2014; Kovtun et al., 2015). The exact way in which caveolin and cavin interact and the role of cavin specifically in smooth muscle is not yet clear; however, a cavin knockout mouse has been produced (Sward et al., 2014). In this knockout animal not only were arterial expression of cavin-1, cavin-2, and cavin-3 reduced but also all isoforms of caveolin were reduced. As a result, caveolae were absent from both smooth muscle and endothelial cells. An enhanced contractile response to an alpha 1 adrenergic agent was seen, but was likely to be due to the increased thickness of the vascular wall. In contrast, myogenic tone was essentially absent. Surprisingly, blood pressure of the knockout mouse was well maintained, presumably due to opposing influences from smooth muscle and endothelial effects.

Inhibition of caveolin function by a caveolin decoy peptide or by methyl-beta-cyclodextrin has been shown to disrupt ERK activation in vascular smooth muscle (Je et al., 2004). Work using cultured vascular smooth muscle cell models has suggested that caveolin-mediated scaffolding of ERK leads to different functional outputs than actin/calponin-mediated scaffolding (Vetterkind et al., 2013). This concept has not yet been pursued in contractile smooth muscle but illustrates the idea of scaffold proteins regulating the output of kinase cascades toward separate purposes and serving as traffic cops for complex cellular signaling pathways.

IQGAP (IQ motif containing GTPase activating protein) is an ERK-binding and actin-binding protein that has been extensively studied in nonmuscle systems but little studied in smooth muscle systems. In cultured vascular smooth muscle cells, knockdown of IQGAP prevents the phosphorylation and activation of an actin-associated pool of ERK in response to PKC activation. Proximity ligation assays demonstrated direct tethering of ERK1/2 to actin by IQGAP. Interestingly caveolin is also required for activation of this pathway unless ERK is already associated with actin. Caveolin appears to be required specifically for upstream C-raf activation (Vetterkind et al., 2013).

#### 2. Myosin Phosphatase Scaffolds.

Myosin regulation is discussed in detail in section IV below, but multiple pathways have been suggested to coordinate signaling associated with myosin phosphatase (MP), and hence, myosin activity (**Pathway #2**, Fig. 1), and it seems likely that scaffolds play a role to regulate/facilitate these pathways. One MP putative scaffold, M-RIP, also called p116^RIP^, is thought to link active Rho/ROCK to the inhibition of MP (Surks and Mendelsohn, 2003; Mulder et al., 2004; Koga and Ikebe, 2005). Vetterkind and Morgan (2009) reported that another scaffold/adaptor protein, Par-4, also regulates myosin phosphatase activity in contractile smooth muscle. We have described a “padlock” model to explain the actions of Par-4, whereby binding of Par-4 to MYPT1 activates MP. This is postulated to occur by the physical blockade by Par-4 of the MYPT1 inhibitory phosphorylation sites. Conversely, this model indicates that inhibitory phosphorylation of MYPT1 by Zipper-interacting protein kinase requires “unlocking” of the blockade by phosphorylation and displacement of Par-4 (Vetterkind et al., 2010). Whether M-RIP and Par-4 facilitate or antagonize each other’s actions is not known.

The complexity of this system is impressive, but it is expected that the multiple scaffolding proteins and signaling molecules involved in regulating myosin phosphorylation will lead to the development of rational and *selective* therapeutic approaches to cardiovascular disease.

### C. Link to Hypertension

We describe here a number of pathways by which vascular smooth muscle contraction and stiffness are directly regulated and hence will affect blood pressure. It should be mentioned that many other indirect pathways are also involved, with a major mechanism being the development of inflammation and subsequent reduction-oxidation reaction (REDOX) signaling pathways (Sorescu et al., 2001; Loirand and Pacaud, 2014). These pathways are triggered by angiotensin-induced signaling, and as a result, inhibitors of the effects of and production of angiotensin are major ways of regulating blood pressure, including blood vessel contraction. For further details, we refer you to Mehta and Griendling (2007) for a review of this topic.

### D. Potential Novel Therapeutic Targets/Approaches/Critical Analysis of Pathway-Specific Inhibitors

#### 1. Rho Kinase Inhibitors.

Y27632, the first ROCK inhibitor described, decreases blood pressure in 11-Deoxycorticosterone acetate (DOCA)-salt rat model of hypertension. A similar effect was obtained with the newer ROCK inhibitors fasudil, SAR07899, in other animal models of hypertension, including the spontaneously hypertensive rat (SHR), angiotensin II-induced hypertension in several animals, and L-NG-Nitroarginine Methyl Ester (L-NAME)-induced hypertension (Uehata et al., 1997; Mukai et al., 2001; Kumai et al., 2007; Lohn et al., 2009). Of note, this class of inhibitors also has a major part of their effect on hypertension through inhibition of inflammatory pathways and cardiovascular remodeling. For more details we refer you to a recent review by Loirand and Pacaud (2014).

#### 2. Endothelin Inhibitors.

The endothelin pathway, linked to PLC and ERK signaling, has been identified as an effective antihypertensive target (Sandoval et al., 2014).

#### 3. Beta Adrenergic Receptor Mediated Inhibition.

Of interest is the fact that beta receptor mediated relaxation of vascular smooth muscle has been reported to decline with age in both the human and animal models. In aortas from Fischer 344 rats, an increase in the level of G-protein receptor kinase-2, which desensitizes the beta adrenergic receptor by phosphorylation of the receptor has been reported to increase with age (Schutzer et al., 2005), and thus inhibitors of G-protein receptor kinase-2 may promote beneficial restoration of beta receptor mediated vasodilation.

## IV. Regulation of Smooth Muscle Myosin

### A. Overview of Regulation of the Smooth Muscle Actomyosin ATPase and 20kda light chain Phosphorylation/Smooth Muscle Activation

**Fig. 4.**
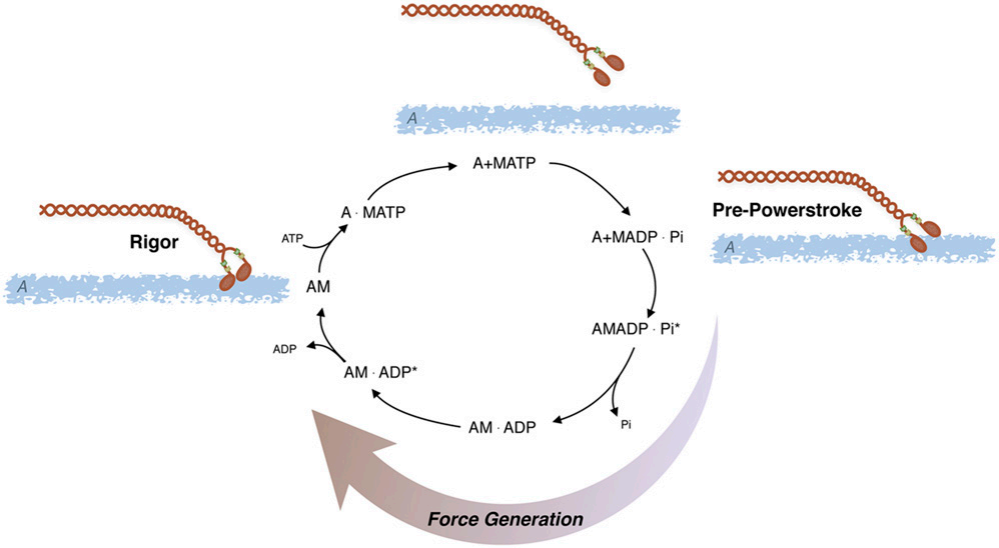
AMATPase: Actomyosin ATPase cycle; ATP is hydrolyzed by myosin (M) and the subsequent interaction of myosin with actin (A) produces force and/or displacement (see text for details).

The crossbridge cycle describes the development of force through a series of complexes between actin (A), myosin (M), ATP, and its hydrolysis products, ADP and Pi (Sweeney and Houdusse, 2010) (Fig. 4 and the termination of **Pathway #1**; Fig. 3). Beginning in the rigor state (AM), ATP binding to AM results in rapid dissociation of AM, forming an A+M-ATP state, and then ATP is hydrolyzed by myosin. After hydrolysis, the crossbridge enters a weakly attached, pre-powerstroke AM-ADP-Pi state, and then transitions to a strongly bound, force producing AM-ADP-Pi state. After Pi release from the AM-ADP-Pi state, the crossbridge enters a AM-ADP state, which then isomerizes to a high force generating state (AM-ADP) followed by ADP release and returning to the rigor state (AM). MgATP subsequently binds to the AM state, causing rapid crossbridge detachment, and then another crossbridge cycle commences. The duty cycle is defined as the proportion of time crossbridges spend in strongly attached states divided by the time for the total crossbridge cycle (De La Cruz and Ostap, 2004); high duty cycle motors are capable of processive movement (i.e., dynein, myosin V), whereas skeletal muscle myosin has a low duty cycle that prevents the development of an internal load from strongly bound crossbridges, which would decrease shortening velocity. Although the crossbridge cycle for all types of myosin is frequently described in this generic manner, differences exist between the kinetics of skeletal, cardiac, and smooth muscle and even within different smooth muscle tissues, requiring changes in the crossbridge cycle to explain the differences in AMATPase rates (Rosenfeld et al., 2000).

The smooth muscle AMATPase is similar to that of striated muscle, albeit the kinetics are slower. The kinetics and individual rate constants of the steps in the actomyosin ATPase have been defined in a number of studies (Rosenfeld et al., 2000; Baker et al., 2003; Haldeman et al., 2014), and similar to other myosin IIs, the ATPase is limited by phosphate release or the transition from weak to strong binding states (Haldeman et al., 2014). Both cardiac and skeletal muscle myosin is functionally on, i.e., myosin will hydrolyze ATP in the presence of actin. Smooth muscle (SM) myosin will hydrolyze ATP in the presence of actin, albeit very slowly; however, after phosphorylation of the 20-kDa regulatory myosin light chain (RLC), the rate of hydrolysis is increased (Chacko et al., 1977; Ikebe and Hartshorne, 1985; Ikebe and Morita, 1991; Ellison et al., 2000) due to an ∼1000-fold increase in the rate of product release (Sellers and Adelstein, 1985). Thus, changes in RLC phosphorylation regulate smooth muscle activation and relaxation.

In smooth muscle, in addition to RLC phosphorylation regulating the AMATPase, it also controls the structure of SM myosin and filament formation (Ikebe and Hartshorne, 1985). In the absence of RLC phosphorylation, myosin is in the 10S conformation [high sedimentation velocity and low ATPase (Ikebe and Hartshorne, 1985), with the tail of myosin bending back over the head neck junction interacting with the regulatory light chain (Jung et al., 2011; Salzameda et al., 2006)]. After RLC phosphorylation, the interaction of the myosin tail with the RLC is perturbed (Jung et al., 2011), and myosin exists in an extended conformation [6S, low sedimentation velocity, high ATPase (Ikebe and Hartshorne, 1985)] and also forms filaments (Applegate and Pardee, 1992). Other investigators have suggested that in the absence of RLC phosphorylation, the interaction of the NH_2_-terminal region of caldesmon (see Fig. 3 and section III.A.1) with the myosin crossbridge disrupts the interaction of the myosin head with its neck-tail region to promote a transition from the 10S to the active, 6S conformation (Wang, 2008). Nonetheless, in human smooth muscle, there is significant pool of 10S myosin that can be converted by changes in cellular conditions to 6S myosin that can then assemble into side polar thick filaments (Milton et al., 2011). These data could suggest that in addition to the regulation of the AMATPase, changes in RLC phosphorylation could regulate the formation of myosin filaments within the smooth muscle during activation and relaxation (Pratusevich et al., 1995; Ali et al., 2007; Liu et al., 2013; Seow, 2013; Lan et al., 2015); however in vivo, the ability of RLC phosphorylation to regulate filament formation is controversial (Seow, 2015; Somlyo, 2015).

The level of RLC phosphorylation is defined by the relative activities of myosin light chain kinase (MLCK) and MLC phosphatase (Gong et al., 1992), i.e., RLC phosphorylation is related to MLCK/(MLCK+MLC phosphatase). Thus, changes in the activity of either MLCK or MLC phosphatase will change RLC phosphorylation and force or vascular tone. MLCK is regulated by Ca^2+^-calmodulin (Ikebe and Hartshorne, 1985), whereas MLC phosphatase activity is regulated by a number of signaling pathways (Hartshorne et al., 1998). At a constant [Ca^2+^], a decrease in MLC phosphatase activity increases SM RLC phosphorylation and force to produce Ca^2+^ sensitization (Somlyo and Somlyo, 2003), whereas an increase in MLC phosphatase activity decreases SM RLC phosphorylation and force to produce Ca^2+^ desensitization (Somlyo and Somlyo, 2003).

MLC phosphatase is a holoenzyme (Hartshorne et al., 1998) consisting of a catalytic subunit, a 20-kDa subunit of unknown function, and a myosin targeting subunit (MYPT1). Alternative splicing of a 123-bp central exon results in MYPT1 isoforms that differ by a 41-aa central insert (CI), referred to as M130 and M133 (Hartshorne et al., 1998). Additionally, alternative splicing of the 3′ exon (Khatri et al., 2001) is responsible for generating MYPT1 isoforms that differ by the presence or absence of a carboxy-terminal leucine zipper (LZ). Thus in humans and other species, alternative splicing generates four MYPT1 isoforms that differ by the presence or absence of a CI and LZ: CI+LZ+, CI-LZ+, CI+LZ−, CI-LZ− (Hartshorne et al., 1998).

### B. Guanine Nucleotide Exchange Factor Signaling, Rac/Rho, and Analysis of Inhibitors

**Fig. 5.**
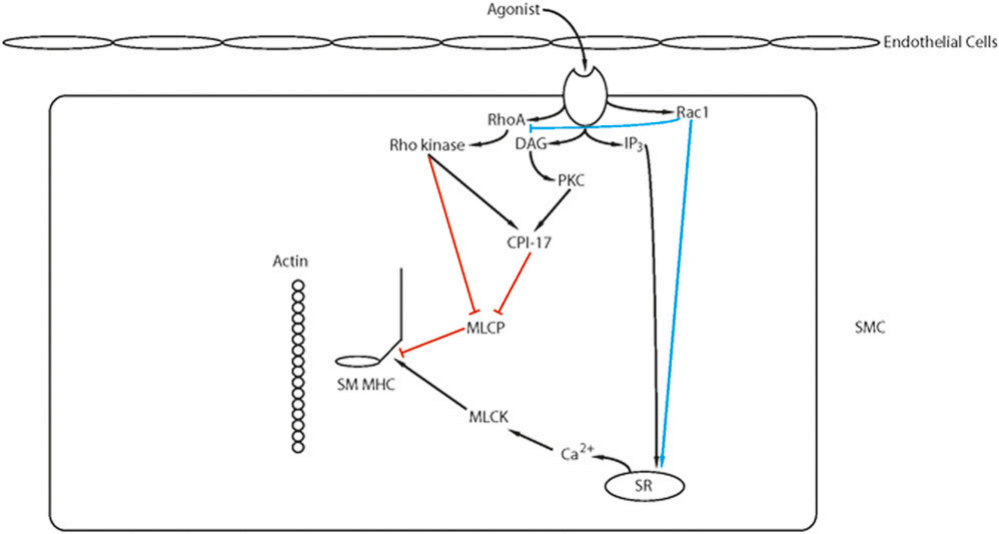
Ca^2+^ sensitization: Agonist activation of G-protein coupled receptors activates several signaling pathways (IP_3_, RhoA/Rho kinase, PKC, RAC1) that modulate Ca^2+^ release from the SR and/or lead to Ca^2+^ sensitization of the contractile filaments (see text for details).

The Rho GTPases are within the RAS superfamily of small G proteins (Jaffe and Hall, 2005), which exist as either active GTP-bound and inactive GDP-bound forms. The conversion between active and inactive forms is controlled by guanine nucleotide exchange factors (GEFs), GTPase activating proteins (GAPs), and guanine dissociation inhibitors. The role of RhoA for the regulation of smooth muscle tone has been well described (Fig. 5; Ca2+ sensitization and **Pathway #2**, Fig. 3). RhoA/Rho kinase signaling is activated by G protein-coupled receptors, and the role of this pathway for the inhibition of MLC phosphatase and Ca^2+^ sensitization has been the subject of a number of reviews (Arner and Pfitzer, 1999; Somlyo and Somlyo, 2003; Puetz et al., 2009). The activation of Rho kinase has been demonstrated to phosphorylate CPI-17 at Thr38 (Eto et al., 1995; Kitazawa et al., 2000), PHI-1 at Thr57 (El-Touhky et al., 2005, 2006), and MYPT1 (Trinkle-Mulcahy et al., 1995) at both T696 and T850 (Muranyi et al., 2005). MYPT1 phosphorylation at T696 has been demonstrated to decrease MLC phosphatase activity to increase force at a constant Ca^2+^ (Kitazawa et al., 1991a), whereas the phosphorylation of MYPT1 at T850 dissociates the holoenzyme, which results in a decrease in MLC phosphatase activity (Velasco et al., 2002), which produces Ca^2+^ sensitization. Similarly, agonist activation of G-protein-coupled receptors has been demonstrated to lead to the activation of PKC, which phosphorylates both CPI-17 at Thr38 (Eto et al., 1995; Kitizawa et al., 1991b) PHI-1 at Thr57 (Eto et al., 1999). When phosphorylated, these proteins will bind to the catalytic core of the catalytic subunit of MLC phosphatase to decrease phosphatase activity (El-Toukhy et al., 2006; Eto et al., 2007). In addition to Rho kinase, Zip kinase will phosphorylate MYPT1 (MacDonald et al., 2001a) as well as CPI-17 (MacDonald et al., 2001b), and integrin-linked kinase will phosphorylate MYPT1 (Kiss et al., 2002; Muranyi et al., 2002) as well as CPI-17 and PHI-1 (Deng et al., 2002).

There are a number of studies demonstrating Rho kinase signaling mediates both MYPT1 phosphorylation and Ca^2+^ sensitization (Somlyo and Somlyo, 2003; Puetz et al., 2009). However, in smooth muscle of the guinea pig ilium, although inhibition of Rho kinase decreased the carbachol-induced increase in Ca^2+^ sensitivity, it had no effect on MYPT1 phosphorylation (Pfitzer, 2001). In this study, although staurosporin prevented MYPT1 phosphorylation, specific PKC inhibitors had no effect on the phosphorylation of MYPT1, which could suggest in this tissue Zip kinase is involved in a physiologically important signaling pathway for Ca^2+^ sensitization. In bladder smooth muscle, others have demonstrated that there is constitutive phosphorylation of MYPT1 at T696, which was unaffected by inhibition of either Rho kinase or PKC, whereas MYPT1 phosphorylation at T850 was primarily mediated by Rho kinase (Chen et al., 2015). These investigators generated T696A and T850A MYPT1 mutant mice to demonstrate that the MYPT1 phosphorylation at T696, but not T850, is important for increasing RLC phosphorylation and Ca^2+^ sensitization during the sustained phase of force maintenance. These results demonstrate that during activation of smooth muscle, the physiologically important signaling pathways mediating both MYPT1 phosphorylation as well as Ca^2+^ sensitization are agonist as well as tissue specific.

Although the role of RhoA in the regulation of Ca^2+^ sensitization of smooth muscle is established, the role of other Rho GTPases for force regulation in smooth muscle is not well defined. Pak1, a downstream target of Rac1, has been demonstrated to inhibit MLCK and relax permeabilized intestinal smooth muscle (Wirth et al., 2003). However in airway smooth muscle, both the knockout or inhibition of Pak reduces tone (Hoover et al., 2012), and Pak3 has been shown to induce a Ca^2+^-independent contraction of permeabilized smooth muscle (Van Eyk et al., 1998; McFawn et al., 2003). Furthermore, Pak also has been demonstrated to phosphorylate CPI-17 (Takizawa et al., 2002). Recently, the role of Rac1 in force regulation has been further delineated (Rahman et al., 2014). Using a smooth muscle-specific, conditional Rac1 KO, these investigators demonstrated that the decrease in Rac1 reduced force in both bladder and saphenous arterial smooth muscle. Furthermore, the inhibition of Rac1 with EHT1864, which affects nucleotide binding, decreased the Ca^2+^ transient and force produced in response to depolarization, agonist activation, and activation of PKC. However, inhibition of Rac1 with NSC23766, which blocks the interaction of Rac1 with GEFs, decreased force for phenylephrine (*α*-agonist) activation but increased the force produced by stimulation with both prostaglandin F2*α* and thromboxane (U46619). These results suggest that Rac1 signaling is agonist dependent and can result in either an enhancement of the Ca^2+^ transient and force or an inhibition of Ca^2+^ sensitization.

The well documented role of RhoA/Rho kinase signaling for the inhibition of MLC phosphatase and Ca^2+^ sensitization (Somlyo and Somlyo, 2003) could suggest that Ca^2+^ sensitization of vascular smooth muscle cells contributes to the molecular mechanism for the increase in vascular tone and/or systemic vascular resistance that produces hypertension. Consistent with this hypothesis are the results demonstrating that in SHR compared with control rats, both the sensitivity to G-protein-coupled agonists and the magnitude and sensitivity of Ca^2+^ sensitization are increased (Satoh et al., 1994) as well as studies defining the role of G_12_-G_13_-induced activation of Rho kinase-mediated Ca^2+^ sensitization for the development of DOCA salt-sensitive hypertension (Wirth et al., 2008). Furthermore, the infusion of the Rho kinase inhibitor Y-27632 reduced blood pressure in normal Wistar rats, as well as several animal models of hypertension including the SHR and DOCA salt-sensitive rat models of hypertension (Uehata et al., 1997), as well as L-NG-Nitroarginine Methyl Ester (L-NAME)-induced hypertension (Seko et al., 2003). Additionally, the expression and activity of Rho kinase are higher in vascular smooth muscle isolated from the SHR compared with controls (Mukai et al., 2001), and the specific Rho kinase inhibitor fasudil (Uehata et al., 1997) reduced blood pressure in the SHR model of essential hypertension (Mukai et al., 2001).

Rho/Rho kinase signaling has also been implicated as an important contributor for the regulation of vascular tone in other mammals; the Rho kinase inhibitor HA1077 has been shown to dilate canine coronary arteries (Asano et al., 1989). Furthermore, Rho kinase is upregulated in a porcine model of coronary vasospasm, and in this model, inhibition of Rho kinase with Y-27632 decreases coronary vasospasm (Kandabashi et al., 2000). In humans, fasudil has been demonstrated to be effective in treating the cerebral vasospasm associated with subarachnoid hemorrhage (Tanaka et al., 2005; Kim et al., 2006), and fasudil is approved for treating patients with pulmonary hypertension (Archer et al., 2010). In humans, Masumoto et al. (2001) demonstrated that brachial artery infusion of fasudil did not decrease systemic blood pressure, but did produce a dose-dependent increase in forearm blood flow in hypertensive patients, but not controls without hypertension. Sodium nitroprusside, on the other hand, resulted in comparable increases in forearm blood flow in both hypertensive and normal humans. These results suggest that an increase in RhoA/Aho kinase signaling may be involved in the molecular mechanism that produces essential hypertension, and development of drugs that target this pathway could represent an effective, novel class of therapeutic agents.

### C. Phenotypic Switching of Contractile Proteins during Development and Disease: Role of MYPT1 in Ca^2+^ Sensitization/Desensitization

#### 1. Smooth Muscle Myosin Heavy Chain.

Four isoforms of the smooth muscle myosin heavy chain (SM MHC) are produced by alternative splicing of a single gene. Alternative splicing of a 5′-site produces two isoforms that express a unique aa sequence of either 43 (SM1, 204 kDa) or 9 (SM2, 200 kDa) residues at the carboxy terminus of the SM MHC tail (Babij and Periasamy, 1989; Nagai et al., 1989), whereas alternative splicing of a 21-bp insert produces a difference of 7 aa near the ATP binding site of the SM MHC (Kelley et al., 1993). Although SM1 or SM2 homodimers are more common than herterodimers in a single myosin rod, SM1 and SM2 homodimers will copolymerize and assemble into side polar thick filaments (Rovner et al., 2002). Estrogen has been demonstrated to increase the ratio of SM1/SM2 expression, and this shift in the expression of SM1 MHC isoform has been suggested to contribute to changes in the sensitivity to the agonist norepinephrine and KCl depolarization (Paul et al., 2007). However, others have demonstrated in the motility assay, there is no difference in the ability of SM1 and SM2 to translate actin (Rovner et al., 2002). These investigators also demonstrated that although there is no difference in the length of SM1 and SM2 filaments, the differences in SM1 and SM2 at the carboxy terminus of the myosin tail influenced filament packing and stability; SM1 filaments have greater stability (Rovner et al., 2002). Consistent with these results, others have demonstrated that the smooth muscle thick filaments of the SM2 KO are similar in length to that of WT mice, but the SM1 thick filaments isolated from SM2 KO mice are smaller in diameter and there are fewer thick filaments per high-powered field (Chi et al., 2008). However, despite the decrease in the number of smooth muscle myosin thick filaments and a concomitant decrease in the expression of SM1, the force produced by both KCl depolarization and carbachol activation was higher in the SM2 KO (Chi et al., 2008).

The other SM MHC isoform is due to a 7-aa insert at the amino terminus, near the ATP binding site of the SM MHC (Kelley et al., 1993). The 7-aa insert is expressed in both SM1 and SM2 SM MHCs, and the SM MHC with the 7-aa insert has been referred to as SMB, whereas SM MHC lacking the insert is SMA (Kelley et al., 1993). SMB, when compared with SMA, has been demonstrated to have a twofold higher AMATPase activity and a 2.5-fold faster velocity of actin translation in the in vitro motility assay (Kelley et al., 1993). Whether the presence of the insert confers a functional difference to the mechanical properties of smooth muscle was assessed using bladder smooth muscle isolated from a transgenic mouse line; the maximum velocity of muscle shortening of bladder smooth muscle strips from WT (SMB+/+) is higher than that of either of heterozygous (SMB+/−) animals or the SMB KO mice (Babu et al., 2001; Karagiannis et al., 2004). Further analysis of the mechanical responses of Ca^2+^-activated skinned bladder strips to elevated Pi and ADP suggested that the lower shortening velocity of the SMA isoform is due to a slower rate of ADP dissociation or an additional force producing isomerization of the AM-ADP state in the crossbridge cycle (Karagiannis et al., 2003). These results demonstrate that the insert near the SM MHC ATP binding site will alter the mechanical properties of smooth muscle, and the duty cycle of SMA should be higher than SMB, which would be predicted to increase vascular tone and/or vascular resistance and thus blood pressure. Consistent with this prediction are the results demonstrating that compared with WT mice, the isometric force for mesenteric vessels of SMB KO mice was increased (Babu et al., 2004). However, the blood pressure of SMB KO animals compared with WT controls has not been reported, and whether SMA/SMB isoform expression is altered during hypertension has not been investigated.

#### 2. ELC17.

For the 17-kDa essential myosin light chain (ELC17), alternative splicing of exon six, which encodes 44 bp, also produces two isoforms, which differ in the expression of 9 aa at the carboxy terminus of the ELC17 (Nabeshima et al., 1987; Lenz et al., 1989; Hasegawa and Morita, 1992). Exon 6 is excluded in the nonmuscle, more basic isoform (ELC17a), whereas exon 6 is included in the smooth muscle, more acidic isoform (ELC17b) (Hasegawa and Morita, 1992; Kelley et al.,1993). Changes in the ELC17 could affect the stiffness of the SM MHC lever arm, and thus myosin step size and or unitary force. However for ELC17 isoforms, the motility assay does not show any difference in the velocity of actin translocation (Kelley et al., 1993; Quevillon-Cheruel et al., 2000). Nonetheless in fast smooth muscle, the expression of ELC17a and SMA is higher than in slow smooth muscle (Malmqvist and Arner, 1991).

#### 3. MYPT1.

**Fig. 6.**
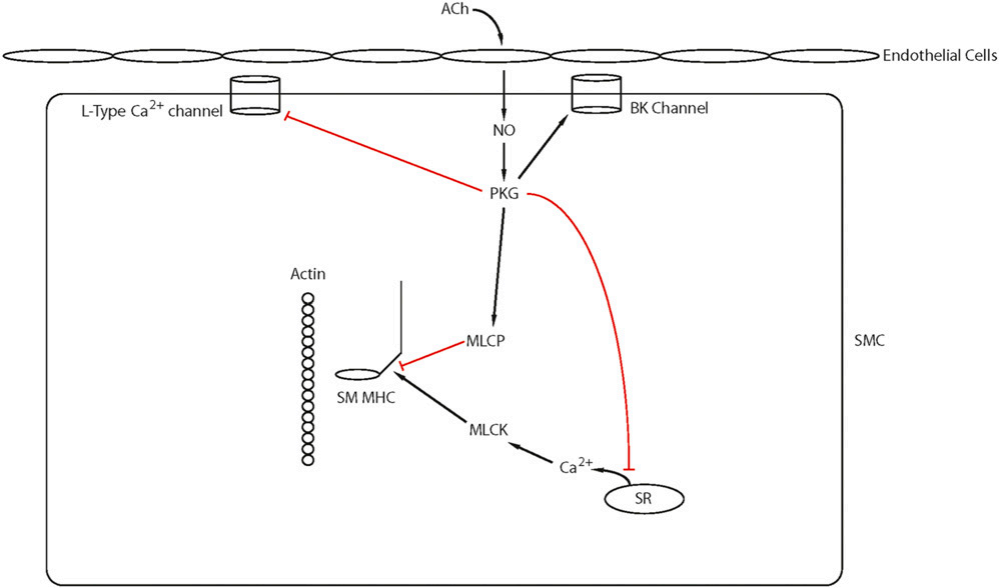
Ca^2+^ desensitization: ACh stimulation of muscarinic receptors on the vascular endothelium leads to the production of NO, and NO diffuses into smooth muscle cells to activate guanylate cyclase. The NO/cGMP signaling pathway relaxes smooth muscle by both decreasing intracellular Ca^2+^ and activating MLC phosphatase, which results in Ca^2+^ desensitization of the contractile filaments (see text for details).

Alternative mRNA splicing produces four splice variant MYPT1 isoforms, formed by the presence or absence of a 43 aa central insert (CI+/−) and carboxy-terminal leucine zipper (LZ+/−). The expression of the CI (residues 512–552) (Shimizu et al., 1994) is both developmentally regulated and tissue specific (Dirksen et al., 2000), and phosphorylation of MYPT1 at Thr696 has been demonstrated to inhibit phosphatase activity (Ichikawa et al., 1996). Although there is evidence that the CI+ MYPT1 isoform may be preferentially phosphorylated during Ca^2+^ sensitization (Richards et al., 2002), a role for the CI for the regulation of smooth muscle contractile properties has not been established. The MYPT1 LZ+ isoform is generated by the exclusion of a 3′- to 31-bp exon, whereas exon inclusion generates a LZ− MYPT1 isoform (Khatri et al., 2001). The aa sequence of the MYPT1 LZ domain is identical from avians to mammals and 75% identical in mammals and worms (Khatri et al., 2001), which could suggest that this domain is important for the regulation of MLC phosphatase activity. Surks et al. (1999) were the first to demonstrate an important functional role for the MYPT1 LZ domain. These investigators demonstrated that the LZ MYPT1 domain was important for the interaction of MYPT1 and PKG (Surks et al., 1999; Surks and Mendelsohn, 2003). Subsequently it has been demonstrated that PKG interacts with the LZ domain (Lee et al., 2007; Sharma et al., 2008) as well as a MYPT1 domain between aa 888 and 928 (Given et al., 2007; Sharma et al., 2008), but the LZ domain is necessary for the PKG-mediated activation of the MLC phosphatase during Ca^2+^ desensitization (Fig. 6,; Ca^2+^ desensitization) and/or nitric oxide (NO)-mediated vasodilatation (Huang et al., 2004). PKG phosphorylates MYPT1 at Ser695 and Ser849, which excludes the Rho kinase-mediated MYPT1 phosphorylation at Thr696 and Thr850, to prevent a Rho kinase-mediated decrease in MLC phosphatase activity (Wooldridge et al., 2004; Nakamura et al., 2007), but MYPT1 phosphorylation at these sites by PKG does not increase MLC phosphatase activity (Nakamura et al., 2007). The mechanism for the increase in MLC phosphatase activity during NO/cGMP-mediated Ca^2+^ desensitization was recently defined; PKG phosphorylates only LZ+ MYPT1 isoforms at Ser668 (Yuen et al., 2011, 2014), and the Ser668 MYPT1 phosphorylation increases the activity of MLC phosphatase (Yuen et al., 2011; Yuen et al., 2014). MYPT1 LZ+/− isoform expression is developmentally regulated, tissue specific (Khatri et al., 2001; Payne et al., 2006), and modulated in animal models of heart failure (Karim et al., 2004; Chen et al., 2006; Ararat and Brozovich, 2009; Han and Brozovich, 2013), pre-eclampsia (Lu et al., 2008), portal hypertension (Payne et al., 2004; Lu et al., 2008), pulmonary hypertension (Konik et al., 2013), nitrate tolerance (Dou et al., 2010), and sepsis (Reho et al., 2015). Furthermore, a decrease in LZ+ expression decreases the sensitivity to NO-mediated vasodilatation (Huang et al., 2004; Yuen et al., 2011, 2014). Thus, the molecular mechanism that underlies the differential response of the vasculature to NO and NO-based vasodilators is in part due to differential expression of LZ+/− MYPT1 isoforms, and furthermore, changes in the relative LZ+/− expression could tune the vasculature between a low-resistance vascular bed (relatively vasodilated), which is NO responsive and Rho kinase/PKC resistant, and a high-resistance vascular bed (relatively vasoconstricted) that is resistant to NO but responsive to Rho kinase/PKC.

The importance of the LZ domain of MYPT1 for the regulation of vascular tone has been established using two different transgenic mice. Michael et al. (2008) produced a transgenic mouse with mutations in the LZ domain of PKG, which disrupts the interaction of PKG with MYPT1. Compared with control littermates, the vascular smooth muscle isolated from these mice were less sensitive to NO-mediated vasodilatation, and thus these mice were hypertensive. In addition and also consistent with an important role for LZ+ MYPT1 isoform expression for the regulation of vascular tone are the results demonstrating that transgenic mice expressing only the LZ+ MYPT1 isoform are more sensitive to NO-mediated relaxation and hypotensive compared with WT littermates (Reho et al., 2015).

Because NO-mediated vasodilation is a fundamental property of the vasculature (Furchgott and Zawadzki, 1980; Furchgott, 1999), preservation of the normal response to NO by maintaining the normal LZ+ MYPT1 isoform expression could improve the outcome for the treatment of diseases of the vasculature. For the treatment of heart failure, both ACE inhibitors (Chen et al., 2006; Chen and Brozovich, 2008) and ARBs (Ararat and Brozovich, 2009) have been demonstrated to preserve both LZ+ MYPT1 expression and the sensitivity to NO-mediated vasodilatation, which could underlie the beneficial effects of inhibition of angiotensin II signaling in the treatment of heart failure compared with other vasodilators (Pfeffer et al., 1992; Yusuf et al., 1992, 2000; Pitt et al., 2000; Granger et al., 2003). Additionally, differential expression of LZ+/− MYPT1 isoforms in patients with heart failure could underlie the mortality benefit of ACE inhibitors in white compared with black patients. ACE inhibitors reduced mortality in white patients, whereas in black patients, although ACE inhibitors did not show a benefit, treatment with the combination of hydralazine and isosorbide dinitrate reduced mortality (Carson et al., 1999).

The regulation for LZ+/− MYPT1 expression is unknown, but LZ+/− MYPT1 expression is both developmental regulated and tissue specific (Khatri et al., 2001; Payne et al., 2006), as well as modulated during disease states (Karim et al., 2004; Payne et al., 2004; Lu et al., 2008; Fisher, 2010). During heart failure (HF), p42/44 MAP kinase is activated and the expression of LZ+ MYPT1 decreases (Ararat and Brozovich, 2009), and further losartan therapy prevents the activation of p42/44 MAP kinase and preserves LZ+ MYPT1 expression (Ararat and Brozovich, 2009). Additionally, the expression of LZ+ MYPT1 isoforms as well as Tra-2*β*, an atypical member of RNA binding proteins, are higher in fast (phasic) compared with slow (tonic) smooth muscle, and in an animal model of portal hypertension, Tra-2*β* is downregulated coincident with the decrease in LZ+ MYPT1 expression (E23 exon exclusion) and the shift to the expression of LZ− MYPT1 isoforms (Shukla and Fisher, 2008). These investigators demonstrated both that Tra-2*β* binds to E23 and transactivates E23 splicing (exon inclusion) to generate a LZ− MYPT1 isoform and deletion or mutation of the Tra2*β* binding site abolished E23 splicing (exon exclusion) to produce a LZ+ MYPT1. These investigators also showed that an siRNA-induced decrease of Tra-2*β* decreased E23 splicing to produce an increase in LZ+ MYPT1 expression, which is consistent with Tra-2*β* expression regulating E23 splicing and LZ+/− MYPT1 expression (Shukla and Fisher, 2008). Further investigation of the signaling pathway for the regulation of LZ+/− MYPT1 isoform expression could reveal novel targets in this pathway. Small molecules could be designed to modulate either total MYPT1 or LZ+ MYPT1 expression, which would both improve both the vascular response to endogenous NO and pharmacological response during the treatment of hypertension as well as a number of other diseases of the vasculature.

### D. Implications for Disease and Treatment

#### 1. Pressurized Resistance Vessels, Implications of the Myogenic Response for Hypertension, and Critical Analysis of Inhibitors.

In most systems, flow and pressure are linearly related; as pressure increases, so does flow. However, small arteries vasoconstrict in response to an increase in pressure and vasodilate in response to a decrease in pressure, and the change in vessel diameter in response to changes in intravascular pressure is referred to as the myogenic response (Bayliss, 1902; Lassen, 1959; Davis and Hill, 1999). The myogenic response is an intrinsic property of the small resistance vessels and does not require flow (Davis and Hill, 1999). Furthermore, because of the intrinsic myogenic response as well as the modulatory actions of vasoactive substances (Bayliss, 1902; Lassen, 1959; Davis and Hill, 1999; Walsh and Cole, 2013), the resistance vessels maintain a constant blood flow over a wide range of perfusion pressures, and this property is termed the autoregulation of blood flow (Bayliss, 1902; Lassen, 1959; Davis and Hill, 1999).

Hypertension is associated with abnormalities of the myogenic response (Immink et al., 2004; Jarajapu and Knot, 2005; Kim et al., 2008b), and thus, the mechanism underlying the myogenic response is important in both health and disease. There are significant regional as well as vessel dimension differences in the magnitude and mechanism governing the autoregulation of blood flow (Jones et al., 1995; Davis and Hill, 1999). Further complicating investigation of the mechanism(s) responsible for the myogenic response are the small size (<200 *μ*m diameter) of the resistance vessels; until recently, investigation of the signaling pathways regulating the myogenic response of the small resistance arteries was not possible. But, recent improvements in the sensitivity of protein phosphorylation levels have allowed for direct investigation of signaling pathways that regulate the myogenic response (Takeya et al., 2008; Johnson et al., 2009; El-Yazbi et al., 2010; Moreno-Dominguez et al., 2013).

Although the signaling pathway for the myogenic response is still being investigated, the first element is the mechanosensor that responds to the changes in intraluminal pressure. Martinez-Lemus et al. demonstrated that blocking integrin function with either anti-integrin antibodies (Martinez-Lemus et al., 2005) or integrin-specific peptides (Martinez-Lemus et al., 2003) results in a significant inhibition of the myogenic response. Furthermore, both the activation of integrins and the myogenic response are associated with tyrosine phosphorylation and the activation of focal adhesion kinase (FAK) and Src family tyrosine kinases (Murphy et al., 2001; 2002). Additionally, agonist stimulation of smooth muscle lead to a tyrosine phosphorylation of the protein paxillin (Pavalko et al., 1995) as well as activation of p42/44 MAPK (Spurrell et al., 2003) and the L-type Ca^2+^ channel (Chan et al., 2010). These data suggest that because of their ability to link the extracellular matrix to the cytoskeleton of the smooth muscle cell, integrins participate in the sensing and transmission of changes in intravascular pressure.

There are a number of studies that have demonstrated an important role for TRP channels Earley and Brayden (2015), specifically TRPC6 and TRPM4 in the myogenic response. Gonzales et al. (2014) demonstrated that selective inhibition of TRPM4 decreased the transient inward cation current induced by membrane stretch. These investigators demonstrated that the generation of IP_3_ by PLC*γ*1 and the subsequent Ca^2+^ release from internal stores is required for both TRPM4 activity and myogenic tone. Furthermore, both TRPC6 inhibitors and antibodies that bind to an extracellular epitope of TRPC6, which block TRPC6 currents, attenuated the stretch-induced activation of TRPM4 current. These investigators also demonstrated that the inhibition of Src nonreceptor tyrosine kinases, which signal through PLC*γ*, decreased myogenic tone. These results suggest that Src tyrosine kinase activity is important in the stretch-induced increases PLC*γ*, which generates IP_3_. Subsequently, IP_3_ binds to the IP_3_ receptor, which stimulates Ca^2+^ release from the SR. Thus, in response to stretch, a local increase in Ca^2+^ generated by both TRPC6 and IP_3_ are important for the activation of TRPM4, which changes membrane potential and increases the conductance of voltage-dependent Ca^2+^ channels, which results in smooth muscle cell contraction to generate the myogenic response.

Additionally, both Ca^2+^ release and Ca^2+^ sensitization of the contractile filaments contribute to the myogenic response. The pressure-induced increase in wall tension leads to depolarization of the smooth muscle cells, which results in opening of voltage-gated Ca^2+^ channels (Knot and Nelson, 1998; Davis and Hill, 1999) and an increase in intracellular Ca^2+^. However, in addition to the pressure-induced increase in Ca^2+^, other mechanisms contribute to the myogenic response (Worley et al., 1991; Osol et al., 2002). Using a highly sensitive biochemical technique, El-Yazbi et al. (2010) demonstrated that only in the presence of myogenic tone, serotonin stimulation produced a Rho kinase-mediated phosphorylation of MYPT1, which induced a Ca^2+^ sensitization of the contractile filaments. Furthermore, this group has also demonstrated that both a Rho kinase-mediated phosphorylation of MYPT1, as well as a Rho kinase- and PKC-mediated increase in actin polymerization are important determinants of the myogenic response (Moreno-Dominguez et al., 2013; El-Yazbi et al., 2015). The activation of the Ca^2+^-dependent tyrosine kinase, Pyk2, has also been demonstrated to occur with KCl depolarization, and although the inhibition of Pyk2 did not change the rapid increase in force and RLC phosphorylation in response to KCl depolarization, Pyk2 inhibition did depress RLC phosphorylation, Thr-696, and Thr-850 MYPT1 phosphorylation and force during the sustained phase of the contraction (Mills et al., 2015). These results suggest that a Ca^2+^-induced activation of Pyk2 leads to an activation of RhoA/Rho kinase and Ca^2+^ sensitization, which is important for force maintenance, or the tonic phase of smooth muscle contraction.

Because hypertension is associated with abnormalities of the myogenic response (Immink et al., 2004; Jarajapu and Knot, 2005; Kim et al., 2008b), antihypertensive agents aimed at the signaling pathway for the myogenic response should be effective for the control of blood pressure. Drugs that decrease Ca^2+^ influx as well as agents that decrease and/or block the activation of Rho kinase signaling such as ACE inhibitors, ARBs, Rho kinase inhibitors should be and are effective antihypertensives. However, novel agents designed to decrease the sensitivity of the mechanosensor such as blocking the activation of integrins or decreasing tyrosine kinase activation could reduce blood pressure. However, tyrosine kinase inhibitors used for the treatment of carcinomas are known to be cardiotoxic (Chu et al., 2007; Force et al., 2007), and hypertension is the most common cardiovascular side effect (Chu et al., 2007). The mechanism by which tyrosine kinase inhibition produces hypertension is not well understood, but hypothesized to be due to fluid retention, endothelial dysfunction, and an inhibition of NO (Cabanillas et al., 2011). These data demonstrate the complex interplay between the myogenic response, circulating vasoactive substances, and the kidney in the regulation of blood pressure.

#### 2. Smooth Muscle Myosin versus Nonmuscle Myosin, Implications for Force Maintenance and Vascular Tone.

There are three classes of nonmuscle (NM) myosin (Golomb et al., 2004), NMIIA, NMIIB, and NMIIC, and both NMIIA and NMIIB are expressed in smooth muscle (Gaylinn et al., 1989; Morano et al., 2000; Lofgren et al., 2003; Eddinger et al., 2007; Yuen et al., 2009; Guvenc et al., 2010; El-Yazbi et al., 2015), whereas NMIIC is only expressed in neuronal tissue (Golomb et al., 2004; Jana et al., 2009). Both NMIIA and NMIIB are able to form bipolar thick filaments (Billington et al., 2013), and similar to SM, myosin RLC phosphorylation promotes filament formation (Ikebe and Hartshorne, 1985; Applegate and Pardee, 1992). In smooth muscle, NM myosin expression represents ∼10–15% of total myosin (Yuen et al., 2009; Konik et al., 2013). Furthermore, NMIIA has been demonstrated to form mixed bipolar filaments with myosin 18A (Billington et al., 2015), and SM1 and SM2 will copolymerize to form filaments (Rovner et al., 2002). However, it is unclear if NM myosin and SM myosin copolymerize to form mixed filaments or whether two distinct pools of SM and NM myosin thick filaments exist within the smooth muscle cells.

The kinetics of the individual steps of the AMATPase of NMIIB (Wang et al., 2003) and NMIIA (Kovacs et al., 2003) are slower than other types of class II myosin. NMIIA and NMIIB have a high ADP affinity, and for NMIIB, the rate of ADP release is similar to that of the steady-state ATPase. Additionally for NMIIB, actin augments ADP binding rather than accelerating ADP release (Wang et al., 2003), which results in NMIIB spending the majority of its kinetic cycle in states that are strongly bound to actin (Rosenfeld et al., 2003; Wang et al., 2003). On the other hand, the rate of ADP release for NMIIA is one order of magnitude faster than the ATPase, which results in NMIIA spending much less of its ATPase cycle in strongly actin bound states (Kovacs et al., 2003). Although the slow kinetics of NMIIA suggest that NMIIA could contribute to force maintenance, the high duty ratio make NMIIB an ideal candidate for the so called “latch crossbridge” (Dillon et al., 1981; Dillon and Murphy, 1982; Hai and Murphy, 1989), and NM myosin may be an important component for the sustained phase of smooth muscle contraction.

Kovacs et al. (2007) recently extended his kinetic studies and demonstrated that ADP release from NMIIB is slow and strain dependent; positive strain increases the rate of ADP release by a factor of fourfold, whereas negative strain decreases ADP release by 12-fold. Load dependence of ADP release prevents NMIIB from slowing either shortening or the rate of force generation by smooth muscle myosin. However for NMIIB, negative strain increases the duty ratio, which would contribute to force maintenance (Kovacs et al., 2007); i.e., for NMIIB, rapid ADP binding and load-dependent ADP release prolongs the attachment of NMIIB to actin at 10–100 *μ*M ADP (at normal MgATP), which would decrease the rate of ATP usage to <0.01 ATP per head per second during force maintenance (Kovacs et al., 2007). Furthermore, during force maintenance both heads of the NMIIB would be attached to actin, which is ideal for a crossbridge that maintains tone.

Similar to NM myosin, the kinetics of SM myosin have been demonstrated to depend on load (Veigel et al., 2003). Using optical tweezers, these investigators demonstrated that the displacement of the SM crossbridge occurs in successive steps of 4 and 2 nm. The duration of the first phase (4-nm displacement) is strain dependent, increasing by twofold with a negative strain and decreasing by twofold with positive strain. These results could suggest that the increase in attachment time of SM myosin due to the negative strain on the SM crossbridge during force maintenance could contribute to the latch state. Furthermore, recent data from optical trap experiments demonstrated that the attachment time of smooth muscle myosin to actin varies with SM RLC phosphorylation (Tanaka et al., 2008); when only one of the two heads of smooth muscle myosin is phosphorylated, the dwell time was fit with a double exponential with rates of 24 second^−1^ and 1 second^−1^, compared with a single rate of 29 second^−1^ when both heads were phosphorylated. These investigators suggested that the long attachment time of the singly phosphorylated myosin could explain the latch state, or force maintenance, in smooth muscle. However, experiments in the optical trap are performed at low ionic strength to both promote actin-myosin interaction and increase interaction times; at physiologic conditions, rates are much faster. Tanaka et al. (2008) also demonstrated that the ATP turnover rate of singly phosphorylated myosin was 30% of that compared with doubly phosphorylated myosin, which contrasts with the results of Rovner et al. (2006) who demonstrated that the ATP turnover rate of myosin with a single phosphorylated head was over 50% of that when both heads were phosphorylated. The reason for the discrepancy between the results for the ATP turnover is unclear. However, it is likely that the single molecule mechanics of NM myosin with the RLC of one or both heads phosphorylated would be similar to smooth muscle myosin, although much slower, making NM myosin a more attractive candidate for a latch crossbridge.

**Fig. 7.**
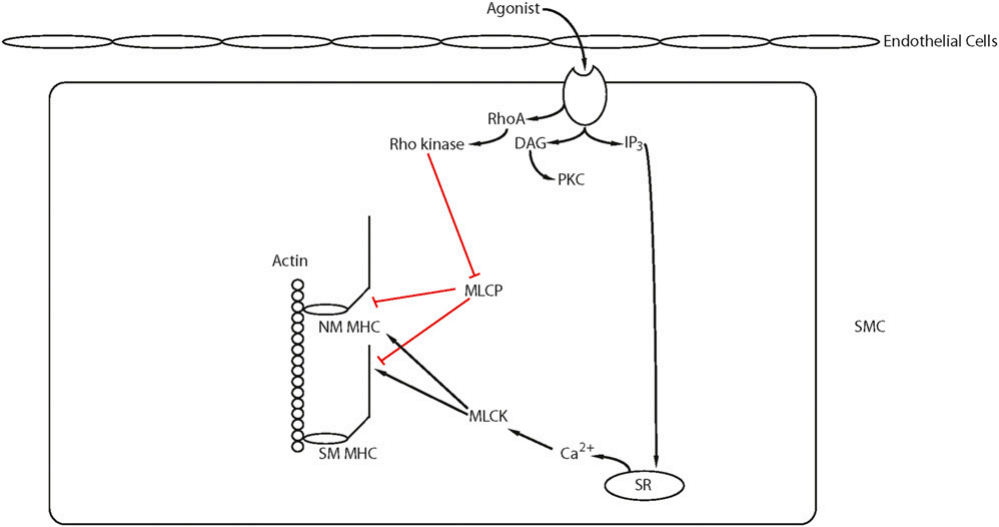
Force maintenance involving either nonmuscle myosin or smooth muscle myosin: There are several proposed mechanisms for force maintenance in smooth muscle including the interaction of actin with either smooth muscle or NM myosin (latch crossbridge) as well as changes in the cytoskeleton (see text for details and see also Fig. 3).

The regulation of NM myosin has been studied in nonmuscle cells (Kolega, 2003), and similar to smooth muscle myosin, NM myosin is regulated by phosphorylation of its regulatory light chain (NM RLC); NM RLC phosphorylation promotes NM myosin filament assembly (Kolega, 2003) and also results in a 10-fold increase in the *V*_max_ of the NM myosin AMATPase (Cremo et al., 2001). In nonmuscle cells, Rho kinase phosphorylates the NM RLC at Ser19 (Kolega, 2003), and in epithelial cells, results are consistent with both MLCK and Rho kinase as important for the phosphorylation of NM RLC (Connell and Helfman, 2006). In epithelial carcinoma cell lines, Rho kinase increases the phosphorylation of the NM RLC at Ser19 phosphorylation of both NMIIA and NMIIB (Sandquist et al., 2006). During KCl depolarization of smooth muscle, both SM RLC and NM RLC phosphorylation increase, and the increase in RLC phosphorylation is not dependent on either Rho kinase or PKC (Yuen et al., 2009). However for angiotensin II (Ang II) activation, inhibition of either Rho kinase or PKC blunted SM RLC phosphorylation, whereas only a Rho kinase-dependent pathway regulated NM RLC phosphorylation (Yuen et al., 2009). These results demonstrate that similar to SM myosin, NM myosin is regulated in smooth muscle, and both MLCK and Rho kinase regulate the activation of NM myosin (Fig. 7).

Recent evidence from mechanical studies also suggests that NM myosin contributes to the force maintenance phase of smooth muscle tissue contraction. Morano et al. (2000) showed that bladder smooth muscle from transgenic mice lacking smooth muscle myosin still contract, albeit with a very slow tonic response, as opposed to the rapid phasic contraction (with transient peaks in force and *V*_max_) characteristic of wild-type bladder smooth muscle (Morano et al., 2000; Lofgren et al., 2003). The force produced by NM myosin in KO tissues is low (Lofgren et al., 2003), which would suggest that NM myosin will not participate in the rapid phase of force activation, but rather the kinetics of NM myosin are tuned for force maintenance (Kovacs et al., 2007). Consistent with this hypothesis are results demonstrating that the inhibition of the NM AMATPase with blebbistatin reduced force maintenance (Rhee et al., 2006); for phasic smooth muscle, blebbistatin did not affect the rapid rise in force, but decreased maintained force, and for tonic smooth muscle, blebbistatin decreased force maintenance. However, although blebbistatin is thought to be specific for the inhibition of the NM myosin AMATPase (Straight et al., 2003), the specificity of blebbistatin for the NM versus SM AMATPase has been questioned (Eddinger et al., 2007). Nonetheless, consistent with a role of NM myosin for force maintenance are the results with heterozygous NMIIB KO mice; when compared with WT control mice, force maintenance is depressed by 25% in smooth muscle of heterozygous NMIIB KO (Yuen et al., 2009). Interestingly, Sward et al. (2000) demonstrated for carbachol activation of guinea pig ileum that inhibition of Rho kinase did not affect peak force but decreased force maintenance, and Rho/Rho kinase signaling regulates NM RLC phosphorylation (Yuen et al., 2009). These results are consistent with NM myosin participating in force maintenance (Morano et al., 2000; Lofgren et al., 2003; Rhee et al., 2006; Yuen et al., 2009; Guvenc et al., 2010), and the inhibition of Rho kinase would lead to a decrease in the activation of NM myosin (Yuen et al., 2009), which results in a reduction in both the force maintenance phase of smooth muscle contraction and vascular tone (SVR). It is also interesting to speculate on the contributions of other pathways, which have been demonstrated to be important contributors for changes in vascular tone for the regulation of NM myosin including tyrosine kinase (Moreno-Dominguez et al., 2013; El-Yazbi et al., 2015; Mills et al., 2015).

#### 3. Force Maintenance/Latch and the Regulation of Vascular Tone: The Tonic versus Phasic Contractile Phenotype and Contributions to Pathogenesis of Hypertension.

Smooth muscle contractile properties have been classified as phasic or tonic (Somlyo and Somlyo, 1968); after activation, for phasic smooth muscle, force rises rapidly to a peak before falling to a lower steady-state level, whereas for tonic smooth muscle, force slowly increases to a sustained steady state. Smooth muscle has also been termed “fast” and “slow” because of the differences in *V*_max_. The molecular mechanism that governs tonic and/or phasic contractile properties has yet to be elucidated, although emerging evidence suggests that there are tonic and phasic contractile phenotypes (see Fisher, 2010). In general, the fast isoforms of the SM MHC (SMB) and ECL17 (ECL17a) are expressed in phasic smooth muscle while slow isoforms (SMA and ECL17b) expression predominates in tonic smooth muscle (Malmqvist and Arner, 1991). Similarly, the expression of NM myosin is significantly higher in tonic smooth muscle (Lofgren et al., 2003; Rhee et al., 2006). In addition, there are differences in the expression of the regulatory proteins in phasic and tonic smooth muscle. The expression of MLCK (Gong et al., 1992), MYPT1 (Woodsome et al., 2001), and the LZ− MYPT1 isoform (Dirksen et al., 2000; Khatri et al., 2001) is higher in phasic compared with tonic smooth muscle. Furthermore, in phasic versus tonic smooth muscle, the expression of CPI-17 is lower (Woodsome et al., 2001), whereas telokin expression is higher (Gallagher et al., 1991; Wu et al., 1998; Herring et al., 2001). These results could suggest that a gene program exists that governs the differential expression of contractile proteins in fast (phasic) versus slow (tonic) smooth muscle (Fisher, 2010), and the contractile phenotype regulates systemic vascular resistance.

Resistance is inversely related to the vessel radius to the forth power (1/r^4^), and thus, SVR is predominantly regulated at the level of the small resistance arteries with a diameter of 50–300 *μ*m. Because of their small size, the molecular contractile phenotype of the resistance vessels have not been fully characterized, but the small resistance vessels express a mixture of fast and slow contractile proteins (Fisher, 2010) and exhibit a mixture of tonic and phasic contractile activity, which is referred to as vasomotion (Peng et al., 2001; Haddock and Hill, 2005). The molecular basis of essential hypertension is unknown, but the molecular contractile phenotype is known to be modulated during disease in both the large conduit vessels (Karim et al., 2004; Chen et al., 2006; Ararat and Brozovich, 2009), as well smaller resistance vessels (Zhang and Fisher, 2007; Han and Brozovich, 2013). In the small resistance vessels, an increase in the relative expression of protein isoforms associated with the tonic contractile phenotype (i.e., an increase in NM myosin expression) or decrease in LZ+ MYPT1 isoform expression would produce an increase in vascular tone and/or SVR, which would produce hypertension.

#### 4. Autoregulation of Vascular Resistance/Flow-Mediated Vasodilatation and Nitric Oxide Signaling with Analysis of Current Inhibitors.

Flow is governed by the simple equation, flow = pressure/resistance, and the ability of NO, or flow, to mediate changes in vascular tone is considered a fundamental property of the vasculature (Furchgott, 1999). The autoregulation of blood flow to a vascular bed maintains a constant flow over a wide range of pressures to ensure that perfusion is maintained despite either hypo- or hypertension. The mechanism governing the autoregulation of blood flow has yet to be fully elucidated but is known to be dependent on the intrinsic myogenic response as well as the modulatory actions of vasoactive substances (Lassen, 1959; Walsh and Cole, 2013), which importantly includes the vascular response to NO. NO is produced by the vascular endothelium in response to shear stress. The NO produced by the endothelium diffuses into the smooth muscle cells where it activates the soluble pool of guanylate cyclase and results in an increase in cGMP. cGMP activates protein kinase G (PKGI), which has a number of targets that produce smooth muscle relaxation (Lincoln, 1989; Schmidt et al., 1993; Alioua et al., 1998; Fukao et al., 1999; Lincoln et al., 2001), including myosin light chain (MLC) phosphatase (Surks et al., 1999). As perfusion pressure increases, the subsequent increase in blood flow will increase shear stress on the endothelial cells, which will stimulate NO production and a subsequent vasodilatation to decrease flow. Conversely, a decrease in perfusion pressure will decrease endothelial shear stress and NO production, and the resulting vasoconstriction will increase flow. Therefore, the NO-induced changes in vascular resistance can be viewed as part of a negative feedback loop that blunts the myogenic response; the myogenic response generates a vasoconstriction with an increase in pressure and vasodilatation with a decrease in pressure (Bayliss, 1902; Lassen, 1959; Davis and Hill, 1999; Walsh and Cole, 2013). Thus, NO production regulates vascular resistance and is essential for the normal regulation of blood flow.

The sensitivity and response of the vasculature to NO and NO-based vasodilators are well known to be heterogenous, and the molecular basis for this variable response to NO is controversial. Nonetheless, during NO signaling, activation of the MLC phosphatase requires the expression of a LZ+ MYPT1 isoform (Surks et al., 1999, 2003; Huang et al., 2004; Yuen et al., 2011, 2014) and in both health (Khatri et al., 2001; Huang et al., 2004; Payne et al., 2006) and disease (Karim et al., 2004; Payne et al., 2004; Zhang and Fisher, 2007; Lu et al., 2008; Dou et al., 2010; Han and Brozovich, 2013; Konik et al., 2013). The sensitivity of the vasculature to NO-mediated vasodilation is regulated by the relative expression of LZ+/LZ− MYPT1 isoforms; i.e., an increase and/or decrease in LZ+ MYPT1 expression will produce an increase and/or decrease in the sensitivity to NO, respectively. Nitrates and nitrate-based vasodilators are a well-known class of antihypertensive agents that will decrease blood pressure in the acute setting. However, tolerance to nitrates is a well-known phenomenon, which may limit the efficacy of nitrates for the treatment of hypertension, and a decrease in LZ+ MYPT1 expression has been demonstrated to contribute to the molecular mechanism of nitrate tolerance (Dou et al., 2010). In the treatment of heart failure, both ACE inhibitors (Chen et al., 2006; Chen and Brozovich, 2008) and ARBs (Ararat and Brozovich, 2009) have been demonstrated to maintain the normal expression of LZ+ MYPT1 expression and sensitivity to NO-mediated vasodilatation. In essential hypertension, both whether changes in LZ+ MYPT1 expression contribute to the molecular mechanism producing this disease and strategies to improve LZ+ MYPT1 expression and the sensitivity to NO-based vasodilators for the treatment of hypertension have not been investigated.

#### 5. Mouse Models (Contractile Protein Knockout) and Implications for Hypertension.

Several strains of genetically modified mice have been produced to evaluate the contribution of abnormalities in the regulation of vascular tone and/or vascular dysfunction to hypertension (Pfeifer et al., 1998; Brenner et al., 2000; Chutkow et al., 2002; Zhu et al., 2002; Tang et al., 2003; Michael et al., 2008; Wirth et al., 2008; Qiao et al., 2014), and experimental results and their implications will be discussed in this section.

Ca^2+^ signaling is well known to be important for the regulation of vascular tone (Arner and Pfitzer, 1999); the activation of voltage-gated Ca^2+^ channels increases cytoplasmic Ca^2+^ and results in vasoconstriction. However, the increase in Ca^2+^ also activates Ca^2+^-activated potassium channels (BK channels), and Ca^2+^ activation of these channels results in a membrane hyperpolarizing current that opposes vasoconstriction (Nelson et al., 1995) and K^+^ channel openers, such as nicorandil, hyperpolarize smooth muscle to produce vasodilatation (Nelson et al., 1990). Brenner et al. (2003) deleted the *β*1 subunit of the BK channel in mice and demonstrated that, compared with controls, the open probability of BK channels was 100-fold lower and the BK current in response to Ca^2+^ sparks was impaired in vascular smooth muscle from the *β*1 KO animals. Furthermore, myography demonstrated that cerebral arteries from the *β*1 KO animals mice were more constricted at all pressures, which resulted in hypertension in these animals. Additionally, BK channels have been demonstrated to increase their conductance in response to NO/cGMP signaling (Alioua et al., 1998).

In smooth muscle, contractile agonists have been demonstrated to activate the G-proteins G_q_ and G_11_ and stimulate phospholipase C, which leads to an increase intracellular Ca^2+^ and activation of MLCK to increase RLC phosphorylation and force (Somlyo and Somlyo, 2003). However, many vasoconstrictors also couple with G_12_ and G_13_ to activate Rho kinase-mediated signaling, which inhibits MLC phosphatase to produce a Ca^2+^-independent increase in force (Somlyo and Somlyo, 2003). Wirth et al. (2008) produced mice with smooth muscle-specific ablation of G_q_-G_11_ or G_12_-G_13_ to investigate the relative contributions of G_q_-G_11_ versus G_12_-G_13_ on vascular tone and the development of hypertension. In aortic smooth muscle isolated from the G_q_-G_11_ KO mice, the contractile response to both phenylephrine and Ang II was completely blocked, whereas the response to serotonin, endothelin, vasopressin, and the thromboxane analog U46619 was inhibited. Ablation of G_12_-G_13_ had no effect on the dose-response relationship for phenylephrine or serotonin, but it reduced the steady-state force produced by Ang II, endothelin, vasopressin, and U46619. Furthermore, compared with WT mice, blood pressure was no different in the mice with ablation of G_12_-G_13_, but significantly lower in the G_q_-G_11_ KO mice, indicating that the normal regulation of blood pressure requires G_q_-G_11_ signaling. Additionally, ablation of either G_q_-G_11_ or G_12_-G_13_ attenuated the increase in blood pressure produced by DOCA-salt treatment, which suggests that although G_12_-G_13_ signaling and the subsequent activation of a Rho kinase pathway leading to Ca^2+^ sensitization is not required for maintenance of normotension, it contributes to the development of DOCA-salt-dependent hypertension. These investigators also demonstrated that the RhoGEF protein LARG is important for the G_12_-G_13_-mediated activation of Rho kinase and DOCA-induced hypertension.

The importance of the NO/cGMP signaling pathways for the maintenance of a normal blood pressure has been established with several different models. In mice, the disruption eNOS has been demonstrated to eliminate ACh-mediated relaxation of aortic smooth muscle rings, and as would be predicted, the relaxation produced by sodium nitroprusside was no different in eNOS KO and WT animals. This defect in vascular reactivity resulted in hypertension in the eNOS KO mice (Huang et al., 1995). Similarly, PKGI KO mice were hypertensive compared with WT littermates (Pfeifer et al., 1998). These investigators demonstrated compared with WT, that although the response to contractile agonist was not different in aortic rings from the PKGI KO, ACh- and 8Br-cGMP-mediated relaxation was significantly attenuated. Further preincubation of aortic cells with 8Br-cGMP attenuated the Ca^2+^ transient in response to NE in WT aortic smooth muscle cells, but had no effect on the Ca^2+^ transient in the PKGI KO aortic smooth muscle cells.

Most contractile agonists activate G_q_-coupled receptors, resulting in the activation of phospholipase C, the generation of IP_3_, Ca^2+^ release, and the activation of MLCK as well as Rho/Rho kinase and a resulting inhibition of the MLC phosphatase (Somlyo and Somlyo, 1994; Davis and Hill, 1999). Mendelsohn’s group (Tang et al., 2003) demonstrated that the dose-dependent increase in IP_3_ production by thrombin was inhibited by both *S*-nitrosocysteine and 8Br-cGMP. Because NO and cGMP inhibit thromboxane signaling via a PKG-mediated phosphorylation of the cytoplasmic tail of the thromboxane receptor (Wang et al., 1998) and RGS-2 terminates G-protein receptor signaling by accelerating the GTP hydrolysis rate by G*α* subunits (Watson et al., 1996), these investigators (Tang et al., 2003) examined the function of RGS-2 in NO/cGMP signaling cascade. They demonstrated that phosphorylation of RGS-2 by PKG resulted in a translocation of RGS-2 from the cytosolic to particulate fractions and decreased G*α*_q_ GTPase activity. These data show that NO/cGMP-mediated activation of PKG results in a phosphorylation of RGS-2, which increases the activation of G_q_-coupled receptors. Consistent with these data are the results that show agonist-induced force production was increased and the vasodilatory response to NO was reduced in aortic smooth muscle from RGS-2 KO mice, and the resulting increase in vascular tone (relative vasoconstriction) was responsible for the increase in blood pressure in RGS-2 KO mice compared with WT littermates (Tang et al., 2003). The importance of the NO/cGMP signaling cascade for the regulation of vascular tone and blood pressure has also been demonstrated in three recent studies (Michael et al., 2008; Qiao et al., 2014; Reho et al., 2015). Michael et al. (2008) produced a knock-in mouse in which the initial four leucine/isoluecine residues of PKGI*α* were replaced by alanine. This PKGI*α* LZ mutant does not interact with MYPT1 (Surks and Mendelsohn, 2003), and as would be predicted, when compared with WT, aortic rings isolated from the PKGI*α* LZ mutant were less sensitive to ACh- and 8Br-cGMP-mediated relaxation, which produced hypertension. Qiao et al. (2014) produced a conditional MYPT1 KO, which were hypertensive compared with WT controls. The lack of MYPT1, which would disrupt the ability of the MLC phosphatase to target to its substrate, the RLC, resulted in an increase in the phosphorylation of the RLC as well as force in response to both KCl depolarization and contractile agonists. However, surprisingly, the sensitivity of relaxation produced by NO/cGMP signaling was similar in the control and MYPT1 KO mice. The lack of a decrease in the sensitivity to NO of secondary branches of the mesenteric arteries from MYPT1 KO animals could be explained if the conditional KO of MYPT1 increased RGS-2 expression or decreased NO/cGMP-mediated PKG phosphorylation of RGS-2 or the functional replacement of MYPT1 by MBS85, which is a member of the MYPT family that is also expressed in smooth muscle (Hartshorne et al., 2004). Reho et al. (2015) generated a conditional deletion of MYPT1 E24 to produce a LZ+ MYPT1 KI mouse. Vascular tissue isolated for the LZ+ MYPT1 mice was more sensitive to NO/cGMP-mediated relaxation, and as would be expected, the mice were hypotensive compared with controls. Of course, it must be kept in mind that NO, cGMP, and PKG also can dilate vascular smooth muscle by more than one mechanism, thus pathways not involving MYPT1 are also possible but we are not aware of appropriate animal models yet available to test those possibilities.

Mendelsohn’s group has also demonstrated that NO signaling is regulated by estrogens; Zhu et al. (2002) compared the vascular responses in WT, iNOS KO, and estrogen receptor *β* KO mice. Estrogen was found to decrease PE-induced contraction of aortic smooth muscle in WT, but not iNOS KO mice. Furthermore, iNOS is upregulated by estrogen, as well as transfection with estrogen receptor *β*. In vascular rings from estrogen receptor *β* KO mice, compared with WT mice, the contractile response to PE was reduced, which could be the result of a decrease in iNOS in the estrogen receptor *β* KO tissues. The sensitivity of smooth muscle relaxation to sodium nitroprusside was similar in estrogen receptor *β* KO and WT animals. Nonetheless, in the estrogen receptor *β* KO animals, the abnormalities in vascular tone produced hypertension.

The data presented in this section demonstrate the importance of the regulation of vascular tone for the regulation of blood pressure and consistently demonstrate that disruption in the NO/cGMP signaling pathway reduces the sensitivity to NO-mediated vasodilatation, and this decrease in the vascular response to NO and/or NO-based vasodilators produces hypertension.

### E. Summary of Contractile Phenotype and Contributions to Pathogenesis of Hypertension with Analysis of Current Therapies for Hypertension

Diuretics reduce blood pressure by decreasing intravascular volume, which mechanistically fits with a Guytonian view for the regulation of blood pressure (Guyton, 1991), and diuretics, as a class, are useful for treating essential hypertension (ALLHAT and Coordinators for the ALLHAT Collaborative Research Group, 2002; Rosendorff et al., 2007). Given the importance of Ca^2+^ signaling for the regulation of vascular tone (Arner and Pfitzer, 1999; Somlyo and Somlyo, 2003), the benefit of CCB for treating humans with essential hypertension is not surprising (ALLHAT Officers and Coordinators for the ALLHAT Collaborative Research Group, 2002). Similarly, ACE inhibitors and ARBs will decrease the activation of the Ang II G-protein-coupled receptor and inhibit Ang II-stimulated increase in intracellular Ca^2+^ and activation of Rho kinase signaling to decrease vascular tone (Somlyo, 1997). Crowley et al. (2005) demonstrated that the KO of the AT1 receptor in only the peripheral vasculature decreases SVR and blood pressure, and thus, these agents would be expected to and have been demonstrated to be effective therapies of essential hypertension (Guyton, 1991; ALLHAT Officers and Coordinators for the ALLHAT Collaborative Research Group, 2002). In fact, drugs that block any G-protein-coupled receptor, i.e., *α*-receptor blockers (Koshy et al., 1977), would be expected to decrease vascular tone and lower blood pressure. *β*-Blockers decrease the activation of the cardiac *β*-adrenergic receptor, and the subsequent decrease in cAMP and PKA signaling produce negative chronotropic and inotropic response, which decreases blood pressure (Rosendorff et al., 2007). However, in the vascular smooth muscle, an increase PKA activity produces a vasodilatation ((Nakamura et al., 2007; Wooldridge et al., 2004), and *β*-blocker therapy could potentially result in an increase in vascular tone, but because of the inhibition of central sympathetic output, SVR decreases with *β*-blocker therapy (Man in't Veld et al., 1988). Drugs designed to increase K^+^ channel conductance produce smooth muscle hyperpolarization and a resulting vasodilatation of vascular smooth muscle (Nelson et al., 1990; Sobey, 2001; Barbato, 2005), and these agents have been demonstrated to both decrease blood pressure (Wang et al., 2005) and be effective treating angina (Group, 2002). Although NO and NO-based vasodilators will result in a decrease in vascular tone (Furchgott, 1999) and are effective for treating angina (Rosendorff et al., 2007), they have not been demonstrated to be useful in treating essential hypertension (Rosendorff et al., 2007), possibly because of the phenomenon of nitrate tolerance (Munzel et al., 2005).

Although there are a number of effective classes of antihypertensives (Rosendorff et al., 2007), when treating patients with essential hypertension, the selection of an antihypertensive agent does not consider the possible changes in the smooth muscle contractile phenotype that may contribute to the molecular mechanism for essential hypertension. This could explain the variable racial and individual response to drug classes (Cushman et al., 2000; Johnson, 2008; Gupta, 2010). Thus, a critical need for a personalized approach for the treatment of essential hypertension exists.

### F. Potential Novel Targets for Treatment of Essential Hypertension

Despite the effectiveness of current antihypertensive (Rosendorff et al., 2007), many patients with essential hypertension either do not respond to one or more therapeutic agents or have a resistant hypertension that requires treatment with multiple classes of antihypertensives for adequate control of blood pressure. Other than the well-known racial and regional differences in response to various classes of antihypertensives (Cushman et al., 2000; Johnson, 2008; Gupta, 2010), there is no known method to determine for any individual drug class whether a patient with essential hypertension will have a therapeutic response or the magnitude of the response to therapy. Because the molecular mechanism that produces essential hypertension is unknown, the diversity in the response to treatment is not unexpected and demonstrates the importance of identifying the mechanism producing hypertension in each individual. As outlined in the preceding sections, there are a number of changes in the contractile phenotype that could contribute to hypertension, and the contribution of the contractile phenotype could be variable among patients.

NO-mediated vasodilatation is a fundamental property of the vasculature (Furchgott, 1999), and studies of transgenic mice have demonstrated the importance of NO/cGMP signaling for maintenance of normal SVR and blood pressure (Huang et al., 1995; Pfeifer et al., 1998; Brenner et al., 2000; Zhu et al., 2002; Michael et al., 2008; Qiao et al., 2014; Reho et al., 2015). However, nitrate-based vasodilators have not been demonstrated to be effective for the treatment of essential hypertension (Rosendorff et al., 2007), possibly because of nitrate tolerance (Munzel et al., 2005). A number of mechanisms contribute to this phenomenon, but recently a decrease in LZ+ MYPT1 isoform has been suggested to play a role in the molecular mechanism for nitrate tolerance (Dou et al., 2010). Because PKG-induced phosphorylation of LZ+ MYPT1 isoforms and subsequent activation of the MLC phosphatase (Yuen et al., 2011; Yuen et al., 2014) is a key component in the NO/cGMP signaling pathway leading to vasodilatation (Lincoln, 1989), increasing MYPT1 LZ+ expression should be effective for both for reversing nitrate tolerance and the treatment of essential hypertension. The regulation for LZ+/LZ− MYPT1 expression is unknown, but investigators have demonstrated that Tra-2*β* appears to be an important regulator for LZ+/LZ− MYPT1 expression (Shukla and Fisher, 2008). Furthermore, both ACE-inhibitors (Chen et al., 2006) and ARBs (Ararat and Brozovich, 2009) were demonstrated to preserve both the normal LZ+ MYPT1 expression and sensitivity to NO-mediated vasodilation in heart failure, which may explain the benefit of these agents in treating heart failure (Pfeffer et al., 1992; Yusuf et al., 1992; Pitt et al., 2000; Yusuf et al., 2000, 2003; Granger et al., 2003) compared with other vasodilators (Awan et al., 1977), as well as the mortality differences for treatment of heart failure with hydralazine and isosorbide dinitrate versus enalapril in white and black patients (Carson et al., 1999). Investigation of the signaling pathway for the regulation of total MYPT1 as well as LZ+/− MYPT1 isoform expression could reveal novel targets in this pathway that would increase LZ+ MYPT1 expression. Small molecules could be designed to modulate either total MYPT1 or LZ+ MYPT1 expression in vascular smooth muscle, and therapies designed to increase LZ+ MYPT1 expression in vascular smooth muscle would improve both the vascular response to endogenous NO and pharmacological response during the treatment of hypertension. Other targets in the NO signaling pathway could also be exploited for the treatment of essential hypertension; i.e., novel activators of RGS-2 would be expected to decrease the activation of G_q_-coupled receptors (Tang et al., 2003; Watson et al., 1996) and ultimately decrease vascular tone and blood pressure.

NM myosin could represent another novel target for drug development for the treatment of hypertension. The inhibition (Rhee et al., 2006), as well as the KO (Yuen et al., 2009) of NM myosin has been demonstrated to decrease force during the tonic phase of smooth muscle contraction, and thus inhibition of NM myosin would be expected to decrease both vascular tone and blood pressure. As outlined above, there are significant differences in the expression of contractile proteins between tonic and phasic smooth muscle (Fisher, 2010), and resistance vessels express a mixture of fast and slow contractile proteins (Fisher, 2010), as well as tonic and phasic contractile properties (Peng et al., 2001; Haddock and Hill, 2005). The contractile phenotype is known to be modulated during disease in both the large conduit vessels (Ararat and Brozovich, 2009) and smaller resistance vessels (Zhang and Fisher, 2007; Han and Brozovich, 2013), and thus essential hypertension could be due to changes in the gene program governing the contractile phenotype and a resulting change in the contractile properties of the resistance vessels from a phasic to more tonic phenotype. This could suggest that targeting the gene program to enhance the expression of phasic contractile proteins would decrease blood pressure and represent a novel therapy for essential hypertension.

## V. Cytoskeletal Regulation

Regulation of smooth muscle function by myosin isoforms, particularly nonmuscle myosin isoforms is discussed above in section IV. Here we will discuss the cytoskeletal proteins that until recently were assumed to serve a primarily structural role in smooth muscle. There are three types of cytoskeletal proteins in this category: intermediate filaments, microtubules, and actin filaments.

### A. Intermediate Filaments, Dystrophin, Utrophin, and Microtubules

Intermediate filaments have been studied relatively little in contractile smooth muscle but of note is literature that suggests that intermediate filaments may be the glue that sticks together different functional domains of the smooth muscle cellular cytoskeleton. This is based on anatomic studies of several types of smooth muscle (Devine and Somlyo, 1971; Ashton et al., 1975; Small et al., 1986; Siegman, 2014) showing that a population of actin filaments that are not directly contacting myosin surround the actomyosin filament bundles and insert into cytoplasmic “dense bodies.” These cytoplasmic dense bodies in turn are connected to “dense bodies” at the surface of the cell (focal adhesions) by intermediate filaments. Studies on airway smooth muscle have also indicated that intermediate filaments form cable-like structures that connect dense bodies and that these structures have a functional plasticity due to mechanisms, yet to be defined, by which the cable length can be adjusted in a regulatory manner (Zhang et al., 2010). Intermediate filaments in contractile vascular smooth muscle have also been suggested to serve functional roles that are not simply structural. For example, these filaments bind CaMKII and are phosphorylated by this kinase (Marganski et al., 2005), but whether the intermediate filaments are serving a signaling scaffolding role or whether CaMKII is somehow changing the function of the intermediate filaments is not known. Similarly, in airway smooth muscle, PAK has been shown to phosphorylate intermediate filaments (Wang et al., 2006, 2007). Interestingly, antisense knockdown of vimentin in smooth muscle inhibits agonist-induced force development (Tang, 2008), but, again, the exact molecular mechanism involved is not clear.

#### 1. Dystrophin/Utrophin.

Of interest is the fact that smooth muscle contains large quantities of dystrophin and utrophin. These proteins have been little studied in smooth muscle, but in lung and vascular smooth muscle they and the dystroglycan complexes they form connect with caveolin and cavin in caveoli as well as the actin cytoskeleton (Palma-Flores et al., 2014; Sharma et al., 2014). Knockout of dystrophin in a mouse model decreases contractility of tracheal rings (Sharma et al., 2014).

With respect to *microtubules*, as expected, the microtubular network is sparse in nonproliferative, nondividing smooth muscle (Somlyo, 1980). They could serve a transport purpose, but we are unaware of such functions yet being demonstrated in contractile smooth muscle.

### B. Actin

A far more extensive literature has developed with respect to actin in smooth muscle. The actin in contractile smooth muscle can be divided into that associated with smooth muscle myosin, generally called the thin filaments, and the nonmuscle actin cytoskeleton. The mechanisms of regulation of actomyosin have been discussed above. Here we will focus on the emerging role of the nonmuscle cytoskeleton in the regulation of vascular function.

Several groups have demonstrated that vasoconstrictors and myogenic contractions (Cipolla et al., 2002; Rembold et al., 2007; Kim et al., 2008a, 2010; Tejani et al., 2011) regulate the structure of the actin cytoskeleton and its connection to focal adhesions (FAs) (Poythress et al., 2013; Saphirstein et al., 2013, 2015) (**Pathway #4**, Fig. 3). These smooth muscle FAs appear to be essentially identical FAs in cultured cells except for their less dynamic nature (Poythress et al., 2013). Increasing evidence (Hill et al., 2001; Gunst and Zhang, 2008; Sun et al., 2008; Saphirstein et al., 2015) indicates that tension modulates the function of signaling pathways in the smooth muscle tissue/cell and that these biomechanical functions are mediated by remodeling of the smooth muscle focal adhesions and its nonmuscle actin cytoskeletal connections. It is important to emphasize that smooth muscles generally lack tendons for the transmission and summation of contractile forces generated by the muscles. In contrast, in smooth muscles the extracellular matrix (ECM) forms a sort of “intramuscular tendon” to which the integrins attach. It is one function of the focal adhesions, then, to channel, somehow, all contractile force generated by the contractile apparatus through the integrins and to the ECM. The exact mechanisms involved are now an active area of investigation in smooth muscle cells.

Moreno-Dominguez et al. (2014) have reported that a decline in G-actin content occurs in response to pressurization or agonist activation of cerebral resistance arteries, resulting in an increase in contractility in the absence of detectable myosin or actin phosphorylation that could be blocked by Rho kinase and PKC inhibitors. These results pointed to cytoskeletal remodeling contributing to the contractile responses observed. Smooth muscle actin exists as four different isoforms. Alpha smooth muscle actin and gamma smooth muscle actin are the dominant actins associated with myosin in the contractile filaments, what has been called “mini sarcomers,” (Fatigati and Murphy, 1984; Kargacin et al., 1989; Herrera et al., 2005) in vascular and gastrointestinal smooth muscle, respectively. Beta nonmuscle and gamma nonmuscle actins are thought to be present in all smooth muscles (Fatigati and Murphy, 1984; Drew et al., 1991; Kim et al., 2008a). These four isoforms are separate gene products and clearly have different functions and different expression patterns, but interestingly they have highly similar protein sequences with no isoform sharing less than 93% identity with any other isoform (Perrin and Ervasti, 2010). Kim et al. (2008a) mapped the individual actin isoforms in vascular smooth muscle during contraction in response to an alpha agonist and found that the overall F/G ratio increases by about twofold in response to the alpha agonist phenylephrine, but only gamma nonmuscle actin displays a statistically significant increase in polymerization. Interesting, in response to a phorbol ester, 12-deoxyphorbol 13-isobutyrate 20-acetate, only beta nonmuscle actin showed significant evidence of remodeling.

The actin isoforms also appear to define distinct subcellular domains within the geography of the cell. It has been found that alpha smooth muscle actin is associated with contractile filaments, beta nonmuscle actin is associated with cytoplasmic dense bodies and focal adhesions and gamma nonmuscle actin describes in interesting submembranous cortical domain in vascular smooth muscle (Parker et al., 1994, 1998; Gallant et al., 2011). Truly specific gamma nonmuscle antibodies have only recently become available, but earlier studies described qualitatively similar organizations in gut smooth muscle (Furst et al., 1986; Small et al., 1986; North et al., 1994a,b).

### C. Focal Adhesion Remodeling

The smooth muscle FA, by connecting with the nonmuscle actin and intermediate filament cytoskeleton, is thought to serve the purpose of transmitting force generated in the contractile filaments through the integrins to the extracellular matrix and the tissue as a whole. FAs in contractile smooth muscle, like those in cultured, migrating cells, are thought to undergo considerable plasticity and remodeling in response to biomechanical forces, agonists, and hormones. When this has been directly investigated in contractile VSMCs, some, but not all, proteins have been shown to dissociate from the bulk of the FA during vasoconstrictor stimulation. Zyxin and vasodilator-stimulated phosphoprotein (VASP), but notably not FAK, undergo Src-dependent endosomal processing and regulate, through processes not yet fully understood, vascular contractility and stiffness (Poythress et al., 2013). In contrast, in airway smooth muscle, FAK has been shown to be quite mobile, shuttling between FAs at the plasmalemma and the cell interior (Opazo Saez et al., 2004). This highlights differences not only in organ function but also in cellular mechanisms between different types of smooth muscle.

Zyxin and vasodilator-stimulated phosphoprotein (VASP) are FA proteins associated with regulation of actin polymerization. This is consistent with a model whereby in VSMCs, only proteins at the edge of the focal adhesion, furthest from the plasmalemma, are capable of breaking off and undergoing remodeling in smooth muscle. This model is consistent with recent nanoscale super-resolution imaging of cultured fibroblast focal adhesion function described by Kanchanawong et al. (2010). In the Kanchanawong model, their data describe three sublayers within individual FAs: 1) the “integrin signaling layer,” containing integrin and its direct connections such as FAK closest to the plasmalemma; 2) the “force transduction layer,” including vinculin and talin, which are more interior; and 3) the “actin regulatory layer,” containing alpha actinin and zyxin, which is deepest into the cytoplasm.

### D. Link to Hypertension

An interesting scenario occurs immediately after birth when there is a drastic demand for a decrease in pulmonary vascular resistance. This triggers a transition from the intrauterine pulmonary vascular requirement for only 8–10% of cardiac output, whereas the placenta performs the function of the primary site of gas exchange to the independent support of the newborn’s lung function. As a result, in the first days to weeks of the newborn’s life, dramatic vascular remodeling is required. If this fails it is associated with the disorder of persistent pulmonary hypertension of the newborn. Interestingly, the remodeling is not just of the cells of the vascular tissue but also a subcellular remodeling of the actin isoforms within the vascular cells with a persistence of excessive gamma actin being a marker of persistent pulmonary hypertension of the newborn (Fediuk and Dakshinamurti, 2015). Cortical gamma actin polymerization is reported to prevent nuclear translocation of the transcription factor YY1, which downregulates SM22, important for smooth muscle specific differentiation (Sotiropoulos et al., 1999).

A similar link between actin polymerization at a subcellular level within the vascular smooth muscle cell and blood pressure has been suggested by the effect of vasoconstrictor agonists to cause both actin polymerization and inward remodeling of resistance vessels associated with many forms of hypertension. Furthermore, inhibition of actin polymerization prevented the agonist-induced inward remodeling of the resistance vessels (Staiculescu et al., 2013). It is well known that increased intraluminal pressure elicits a myogenic vasoconstriction from resistance vessels and a further increase in blood pressure. This myogenic response has been associated with increased Ca^2+^ entry to the cell, activation of myosin light chain kinase, as well as increased activation of ROCK and protein kinase C pathways that regulate actin polymerization (Moreno-Dominguez et al., 2013).

Further investigation of the signaling pathway for the myogenic response has implicated integrins as the link between the extracellular matrix and the cytoskeleton that senses changes in pressure (Martinez-Lemus et al., 2003, 2005). Because the activation of integrins and the myogenic response is associated with tyrosine phosphorylation and the activation of focal adhesion kinase (FAK) and Src family tyrosine kinases (Murphy et al., 2001, 2002), blocking the activation of integrins or decreasing the activation of tyrosine kinase could be novel targets for treating essential hypertension. Although nonreceptor tyrosine kinases can phosphorylate paxillin and induce actin polymerization (Ohanian et al., 2005) and a PKC-mediated pathway will lead to the phosphorylation, or activation, of PYK2 and Src tyrosine kinases (Hodges et al., 2007; Chang et al., 2012), there is also evidence that receptor tyrosine kinases participate in the regulation of vascular tone. ephrin (EPH) kinases are the largest family of receptor tyrosine kinases, and the ligand for EPH tyrosine kinases are ephrins. Ephrins are also cell surface molecules that transduce signals into cells, and multiple EPHs will interact with multiple ephrins (Pasquale, 2008). Recently, the smooth muscle-specific deletion of the tyrosine kinase EPHB4 was demonstrated to result in decreased contractile responses to the agonist phenylephrine and hypotension in male, but not female, mice (Wang et al., 2015). Contrasting with these results are those demonstrating that blood pressure was unaffected by deletion of the tyrosine kinase EPHB6 in male and female mice, but blood pressure was elevated and the contractile response to phenylephrine was enhanced in castrated male EPHB6 KO mice (Luo et al., 2012; Wu et al., 2012). These results demonstrate that there is interplay between the various members of the non-receptor and receptor tyrosine kinase families as well as other factors that ultimately contribute to the regulation of blood pressure that may provide untapped targets for the development of novel antihypertensive therapeutics.

## VI. Identifying Therapeutic Targets in Vascular Smooth Muscle through Biomechanical Studies

### A. Arterial Stiffness as a Predictor of Negative Cardiovascular Events with Aging

Increased arterial stiffness is a prominent concept in studies of cardiovascular disease (CVD), especially in the context of aging, as an independent biomarker and predictor of hypertension and atherosclerosis, which are associated with increased mortality rates and severe damage to organ systems via stroke, renal failure, and heart disease (Ross, 1993, 1999; Dustan et al., 1996; Blacher et al., 1999; Guerin et al., 2001; Laurent et al., 2001; Cruickshank et al., 2002; Mattace-Raso et al., 2006; Laurent and Boutouyrie, 2007). Given the overwhelming predominance and persistence of CVD as the leading cause of human death worldwide, there is great interest and urgency to develop a thorough understanding of arterial stiffness as a possible cause of, and thus a potential therapeutic target for preventing or treating, CVD in humans.

Assessment of arterial stiffness exists on multiple scale levels. Macroscale techniques that characterize the hemodynamics of pulsatile blood flow are available clinically in humans and more easily interpretable, whereas microscale techniques better elucidate the underlying mechanisms causative of changes in the physical properties of blood vessels (Kohn et al., 2015). In studying arterial stiffness, the predominant challenge moving forward is to establish an integrated model of increased arterial stiffness across macro- and microscopic scales that enable the development of treatments to alleviate or prevent later-onset CVD. Recent studies suggest that vascular smooth muscle represents an attractive therapeutic target for such designs.

#### 1. Pulse Wave Velocity: The Clinical Standard

In vivo arterial stiffness can be measured representatively and noninvasively as pulse wave velocity (PWV). This measurement of the pulse wave generated by cardiac systole is the ratio of the distance it travels along the vascular wall to the time delay between its arrivals at different points along the circulatory pathway. Most commonly, PWV is measured via ultrasound between the carotid and femoral arteries as representative of aortic stiffness. As the current “gold standard” of arterial stiffness measurements, increased PWV is strongly correlated to both aging and CVD (Mitchell et al., 2007; Willum-Hansen et al., 2006; Ben-Shlomo et al., 2014).

The validity of PWV as an in vivo approximation of aortic stiffness is described by the Moens-Korteweg equation:
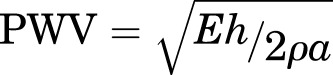
where *E* is the incremental Young’s modulus of the vessel, *h* and *a* are the thickness and radius of the aortic wall, respectively, and *ρ* is blood density. The value of *E* represents a material stiffness of the wall that is more precisely descriptive of its biomechanical properties. The Moens-Korteweg equation is itself dependent upon key assumptions: that the aortic wall is isotropic and homogeneous and that the aorta undergoes small isovolumetric changes in response to pulse wave propagation. Unfortunately, these assumptions do not hold, as demonstrated by previous studies (Clark and Glagov, 1985; Gosling and Budge, 2003); therefore, whereas PWV is an undoubtedly useful prognosticative tool, its accuracy as a biomechanical indicator is unclear.

#### 2. The Importance of Ex Vivo Material Stiffness.

It is appropriate here to distinguish between “functional stiffness”—a relation between pulse pressure applied to a vessel and its resultant deformation, or strain—and “material stiffness,” the normalization of functional stiffness to vessel geometry, most notably vessel diameter and wall thickness (Greenwald, 2007). Material stiffness corresponds most directly to the value of *E* in the Moens-Korteweg relation and is the most independent representation of an intrinsic property to resist deformation in response to an applied force (O'Rourke, 1990; Gamble et al., 1994).

Therefore, PWV can more accurately be described as a standard for functional but not material stiffness. Functional stiffness is derived from material stiffness, but the opposite is not true. In understanding this subtle difference, the diverse terminology used in reference to aortic biomechanical properties may present significant difficulties, compounded by the prevalence of terms reciprocal to stiffness such as “compliance” and “distensibility,” which are sometimes used interchangeably among studies in the context of independence from vessel geometry. The most concise discussion of this complicated canvas is provided by Bank and Kaiser (1998), who cite conflicting results attributable to imprecise terminology and summarize their own study, showing that changes to PWV in brachial arteries, in response to smooth muscle relaxation, are not necessarily predictive of *any* corresponding change in material stiffness.

Analysis of arterial mechanical properties is integral to understanding more broadly the epidemiologic link between arterial stiffness and negative cardiovascular outcomes. Although arterial PWV is an easily demonstrable and a clinically valuable measure of functional stiffness, there is also a strong impetus to evaluate material stiffness of the aortic wall directly to explain how different components of the aortic wall, a highly complex and layered network of interconnected cellular and extracellular elements, contribute to altered or defective mechanisms in disease models. This is most conveniently and commonly done ex vivo by subjecting arterial tissue to mechanical stretch.

### B. Regulation of Arterial Stiffness by Vascular Smooth Muscle

Although numerous studies and quantitative models have highlighted the extracellular matrix as the primary effector of dramatic changes in arterial stiffness due to aging or other cardiovascular defects (Greenwald, 2007; Zulliger and Stergiopulos, 2007; Fleenor et al., 2010, 2012; Holzapfel and Ogden, 2010; Valentin et al., 2011; Wagenseil and Mecham, 2012), vascular smooth muscle cells (VSMCs) are implicated to be major regulatory factors of arterial stiffness overall.

#### 1. Homeostatic Interactions between Cellular and Extracellular Components of the Arterial Wall.

All major biologic components of the arterial wall play integral roles in maintaining normal cardiovascular function. Abnormalities in cellular mechanotransduction, which is regulated by cytoskeletal structures, transmembrane receptors, matrix proteins, and cell-cell and cell-matrix adhesions, have been linked to changes in cell mechanics or sensory machinery, as well as structural alterations in extracellular matrix, which are commonly featured in a wide range of diseases (Ingber, 2003, 2006). Indeed, there is a critical codependence between medial elastin and VSMCs in maintaining structure and function in the arterial wall, with studies showing both that elastin regulates VSMC proliferation (Brooke et al., 2003) and that VSMC knockout phenotypes result in severe degradation of elastin (Fry et al., 2015). VSMCs have been implicated in recruitment of both elastin (Dobrin, 1978) and collagen (Bank and Kaiser, 1998) during their contractile response.

Physiologic factors such as aging and hypertension induce observable physical changes in the wall due to VSMC remodeling (Glagov et al., 1993; Intengan and Schiffrin, 2000). With aging in particular, vascular smooth muscle cells (VSMCs) exhibit increased stiffness (Qiu et al., 2010) and are capable of switching their phenotype from contractile to synthetic, resulting in fundamental changes to cellular function as well as wall structure, because remodeled VSMCs migrate and proliferate in concert with alterations to other wall components, most notably elastin and collagen (Greenwald, 2007; Avolio et al., 2011).

The endothelium has also become an increasingly visible nexus of aging-induced changes, with widespread effects on cellular and extracellular processes throughout the arterial wall and especially those linked to VSM function. Nitric oxide (NO) produced by endothelial cells induces vasodilation and relaxation in VSM (Russell and Watts, 2000). NO bioavailability is reduced with aging, which attenuates this benefit, increasing VSM stiffness as a result. This downstream effect may characterize a powerful positive feedback loop where the increased stiffness leads to further reduction of NO (Cannon, 1998; Avolio et al., 2011).

#### 2. The Focal Adhesion and Actin Cytoskeleton as Regulatory Sites of Arterial Material Stiffness.

**Fig. 8.**
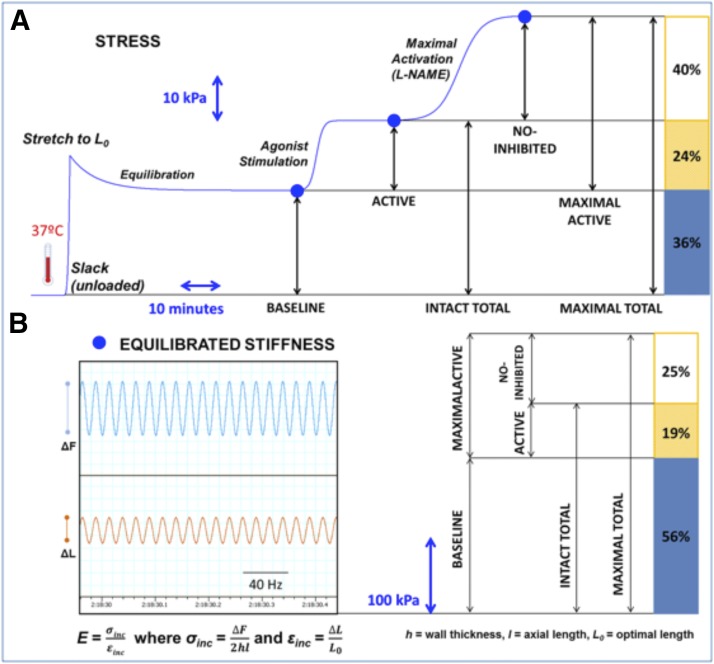
Separability of contractile components to the generation of aortic stiffness. Modified from Gao et al. (2014b).

Recently, attempts to quantify the constituency of total arterial stiffness attributable to VSMC activity have become more prevalent. Ex vivo studies have shown that VSMCs contribute significantly to total stiffness, potentially in conjunction with recruitment of collagen (Barra et al., 1993; Bank et al., 1996). Under optimally physiologic conditions for smooth muscle viability, up to 50% of maximal ex vivo aortic stiffness is observable only after activating VSMCs by alpha agonist and eliminating NO-mediated vasodilation (Gao et al., 2014b), quantifiable as shown in Fig. 8.

**Fig. 9.**
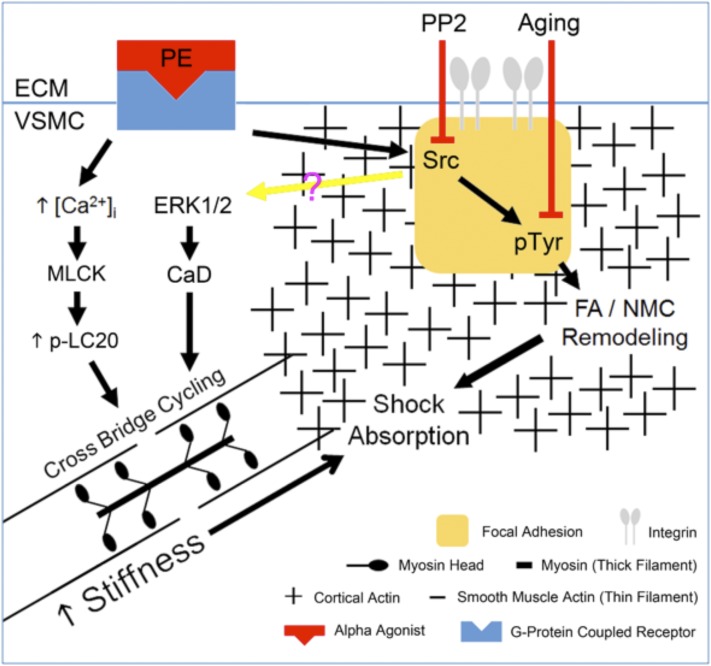
A diagrammatic model of how cytoskeletal remodeling could provide plasticity and an important “shock absorber” for the cardiovascular system (modified from Gao et al., 2014b.)

Therefore, it is of great interest to probe the cellular mechanisms that produce this effect to identify underlying origins of dysfunction and degeneration. A series of studies highlighting stimulated VSMCs have found that focal adhesions connecting the cortical cytoskeleton to the matrix play a critical role in the regulation of signaling cascades triggered by smooth muscle activation, independently of actomyosin crossbridges (Kim et al., 2008a, 2010; Min et al., 2012; Poythress et al., 2013). In particular, disruption of the FAK-Src signaling complex reduces contractility and stiffness, as well as biochemical markers of focal adhesion signaling, induced by smooth muscle activation (Saphirstein et al., 2013; Saphirstein and Morgan, 2014). Interestingly, these mechanisms are defective with aging (Gao et al., 2014b), and may not be limited to the focal adhesions, but are also processes inherent in the cortical actin cytoskeleton (Saphirstein et al., 2015).The normal plasticity of the cytoskeleton may be an important sort of “shock absorber” for the aortic wall that is lost with aging. A model for the focal adhesion and actin cytoskeleton in the VSMC as core machinery regulating arterial stiffness, which is compromised with aging, has emerged from these findings, as shown in Fig. 9.

It has become increasingly clear that VSM may play a major role in the regulation of arterial stiffness.

## VII. Regulation of Vascular Smooth Muscle Cell Function by Epigenetic Mechanisms

Epigenetic changes refer to the heritable alterations in gene expression that occur without changes in genome sequence (Jaenisch and Bird, 2003). The conventional epigenetic mechanisms include DNA methylation and histone modification, which alter the accessibility of transcription factors at DNA regulatory regions, such as promoters or enhancers (Kouzarides, 2007). Over the last decade, RNA-based modifications, which alter the translation of genetic information, have emerged as important regulators of development and disease. Collectively, these mechanisms likely play a role in the remarkable plasticity exhibited by vascular smooth muscle cells (VSMCs). This section will discuss the role of epigenetics in vascular smooth muscle (VSM) biology and describe ways by which these changes may influence the risk of cardiovascular diseases, such as hypertension.

### A. DNA Methylation

DNA methylation involves the covalent addition of a methyl group to the 5′-position of the pyrimidine ring of cytosine residues to form 5-methylcytosine (Lorenzen et al., 2012). This process, catalyzed by DNA methyltransferases, leads to transcriptional repression by blocking the access of transcription factors to response elements in the promoter region of genes. DNA methylation had long been considered irreversible until the recent discovery of the members of the ten-eleven translocation (TET 1-3) family of proteins. TET proteins oxidize 5-methylcytosine to 5-hydroxymethyl cytosine, which ultimately revert to cytosine via the demethylation pathway (Boedtkjer et al., 2011; Lorenzen et al., 2012).

Variable methylation patterns are observed in the genes of VSMCs originating from different tissues (Zhang et al., 2012). Abnormalities in DNA methylation patterns have been observed to associate with vascular diseases and have been studied extensively in atherosclerosis (Dong et al., 2002; Castro et al., 2003; Hiltunen and Yla-Herttuala, 2003). SMCs of atherosclerotic lesions in humans and animal models exhibit reduced levels of 5-methylcytosine. The resulting DNA hypomethylation enhanced switching of VSMCs from a contractile to synthetic phenotype (Laukkanen et al., 1999; Ying et al., 2000; Hiltunen et al., 2002).

### B. Histone Modifications

The modifications at protruding N-terminal tails of histones act as an integral epigenetic tag for chromatin remodeling. The histone tails can be modified by acetylation, methylation, ubiquitylation, and phosphorylation (Cheung et al., 2000; Kouzarides, 2007). The unique pattern of histone modifications in SMCs can alter chromatin packaging, which leads to the differential expression of crucial VSMC genes (Alexander and Owens, 2012). For example, the chromatin structure of the CArG-box [CC(A/T)6GG] motif, essential for tissue-specific expression of SMC marker genes, exists in an easily accessible euchromatin-like form in SMCs but not in non-SMCs (Alexander and Owens, 2012). Various histone modifications, such as dimethylation of H3K4 and H3K79 and acetylation of H4 and H3K9, have been observed in the CArG elements of SM *α*-actin and SM MHC in VSMC gene loci (McDonald et al., 2006). In contrast, these histone modifications are associated with transcriptional silencing in non-SMCs, such as endothelial cells (ECs), embryonic stem cells, and fibroblasts (McDonald et al., 2006). ECs exhibit high levels of H4 acetylation and H3K4 dimethylation at the EC-selective vascular endothelial cadherin gene locus. Increased levels of H4K20 dimethylation of this locus in VSMCs, however, are associated with transcriptional silencing (McDonald et al., 2006). These studies implicate histone modifications as important epigenetic tags in the regulation of SMC determination and differentiation.

#### 1. Histone Acetylases and Histone Deacetylases.

Histone acetylation is characterized by the posttranslational modification of histones, which lead to chromatin remodeling by loosening histone-DNA contacts (Lorenzen et al., 2012). These modifications affect many SMC processes, such as differentiation, phenotypic switching, proliferation/apoptosis, and migration. Acetylation and deacetylation of histones are catalyzed by enzymes known as histone acetylases (HATs) and histone deacetylases (HDACs), respectively. HATs are classified into three families, including GNAT, MYST, and CBP/p300 [cAMP response element-binding protein (CREB)]. HDACs are categorized into four classes in mammals: class I (HDAC1-3, HDAC8), class II (HDAC4-7, HDAC9-10), class III sirtuins (SIRT1-7), and class IV (HDAC11) (Lorenzen et al., 2012). The class I, II, and IV HDACs are Zn^2+^-dependent deacetylases, whereas the class III HDACs, sirtuins, possess nicotinamide adenine dinucleotide (NAD)-dependent deacetylase activity (Lorenzen et al., 2012).

HATs and HDACs work in a dynamic manner to regulate cellular gene expression. Histone acetylation by p300 HAT allows binding of serum response factor to previously inaccessible CArG-containing regions of SMC-specific genes enabling the recruitment of coactivators, such as myocardin, to stimulate expression of *α*-actin (Gabbiani et al., 1981), *γ*-actin, calponin (Duband et al., 1993), SM22*α* (Duband et al., 1993), h-caldesmon (Frid et al., 1992), MLC, and SM MHCs (SM1 and SM2) (Nagai et al., 1988). Retinoic acid treatment of A404 cells, an SMC embryonic cell line, enhances acetylation of histones H3 and H4 at SM *α*-actin and SM MHC CArG-containing promoter regions (Manabe and Owens, 2001). Unlike myocardin, KLF4 (Kruppel-like factor 4) is a transcription factor associated with myogenic repression that regulates SMC phenotypic switching both in vitro and in vivo after vascular injury. KLF4 acts through epigenetic mechanisms to decrease histone H4 deacetylase by recruiting HDAC2 and HDAC5, which block the binding of serum response factor to methylated histones and CArG box chromatin during repression of SMC gene expression (McDonald and Owens, 2007; Yoshida et al., 2008).

Although the studies identifying the role of HATs and HDACs in the regulation of SMC functions warrant further in vivo studies, they highlight the relevance of inhibitors as epigenetic therapeutics in the treatment of vascular diseases. One such study demonstrated that inhibition of p300 HAT activity by Lys-CoA-TAT decreases the effects of retinoic acid on the differentiation of A404 cells toward an SMC lineage (Spin et al., 2010). In a separate study in A404 cells, overexpression of HDACs increased CREB-CArG-dependent SM22 promoter activity, whereas the HDAC inhibitor trichostatin A had the opposite effect (Qiu and Li, 2002). In contrast, HDAC inhibition after tributyrin treatment inhibited migration of cultured VSMCs after hyperacetylation of histone H3 and reduced expression of HDAC7 (Qiu and Li, 2002). Pharmacological inhibition of HDAC activity or knockdown of HDAC expression with small interfering RNAs (siRNA) prevents platelet-derived growth factor-induced VSMC proliferation (Findeisen et al., 2011).

Recently, HATs and HDACs have been found to act on the lysine (K) residues of a wide range of nonhistone proteins and are therefore also denoted as KATs and KDACs, respectively (Europe-Finner et al., 2015). Nonhistone targets for KATs and KDACs include large cellular macromolecular complexes involved in the actin nucleation complex (Choudhary et al., 2009) and other proteins participating in microfilament formation and dynamics (Posern et al., 2004). A majority of proteins involved in smooth and striated muscle contraction have been shown as substrates undergoing acetylation (Karolczak-Bayatti et al., 2011; Lundby et al., 2012). KDAC8 regulates the differentiation and contraction of SMCs by interacting with *α*-actin (Waltregny et al., 2005; Chen et al., 2013), tropomyosin, and cortactin proteins (Chen et al., 2013; Li et al., 2014a). KDAC6 is involved in the modulation of the cytoskeleton, cell migration, and cell-cell interactions by interacting with proteins such as SIRT2 (Valenzuela-Fernandez et al., 2008). KDAC inhibition by trichostatin A and hydroxamic acid was associated with increased protein acetylation and reduction of agonist-stimulated contractions in VSM tissues (Krennhrubec et al., 2007; Karolczak-Bayatti et al., 2011; Chen et al., 2013). In a separate study, during contraction, the cytoskeletal proteins were dynamically regulated through HDAC inhibition and activation, resulting in the regulation of cytoskeletal structure (Kim et al., 2006).

##### a. Histone deacetylases and link to hypertension.

Epigenetic mechanisms influence the changes in vascular structure that occur during hypertension (Lacolley et al., 2012). Angiotensin II (Ang II) at least partially influences vascular remodeling via HDACs in the pathogenesis of hypertension (Xu et al., 2007). Specifically, HDAC5 and HDAC4, after phosphorylation by Ang II, export from the nucleus where they activate myocyte enhancer factor-2, leading to SMC hypertrophy (Xu et al., 2007; Li et al., 2010). Treatment of spontaneously hypertensive rats with HDAC inhibitors, such as trichostatin A or valproic acid, reduced SMC hypertrophy, blood pressure, and vascular inflammation (Cardinale et al., 2010; Bogaard et al., 2011; Usui et al., 2012). All these studies highlight an important therapeutic potential for HDAC inhibitors in the treatment of vascular diseases.

#### 2. Sirtuins.

Sirtuins are a class of proteins that have multiple functions including NAD^+^-dependent deacetylation. Currently, there are seven known mammalian sirtuins (SIRT1-7), located in the nucleus (SIRT1, 6, and 7) (Michan and Sinclair, 2007), cytoplasm sirtuin (SIRT2), or mitochondria sirtuins (SIRT3, 4, and 5) (Michishita et al., 2008). Because SIRTs are multifunctional, they have a plethora of protein targets, including p53, nuclear factor *κ*B, Ku70, FOXO, tubulin, eNOS, BAD, CytoC, Ndufa9, GDH, ACS2, and ISDH2 (Haigis and Sinclair, 2010; Corbi et al., 2013). Extensive reviews are available on the role of sirtuins in aging, calorie restriction, mitochondrial biogenesis, and neurodegenerative diseases (Gagnon et al., 2008; Pfister et al., 2008; Haigis and Sinclair, 2010; Guarente, 2011),

Sirtuins have largely been shown to have protective effects on the vasculature. For instance, SIRT1 modulates vascular biology during hypoxia-induced redox stress by deacetylating the transcription factor, HIF (Hypoxia-inducible factor)-2*α*, which modulates vascular tone and enhances cell survival through induction of antioxidant enzymes to promote angiogenesis (Dioum et al., 2009). The deacetylase activity of SIRT1 has also been shown to regulate the proliferation and migration of VSMCs via the suppression of cellular senescence mediator, p21 protein, and enhancement of senescence-resistant cell replication (Stein and Matter, 2011). Overexpression of SIRT1 increases the activity of metalloproteinase-3 inhibitor, which inhibits cell migration. In addition, SIRT1 also deacetylates and activates eNOS, which enhances NO-induced vasodilation (Mattagajasingh et al., 2007).

Interestingly, recent studies implicated SIRT1 in blood pressure regulation through targeting VSMCs. Overexpression of SIRT1 in VSMCs has been found to modulate blood pressure by downregulation of the Ang II type 1a receptor gene (Miyazaki et al., 2008). Moreover, resveratrol treatment activated SIRT1, resulting in the repression of Ang II type 1a receptor gene transcription to counteract Ang II-induced hypertension in mice (Miyazaki et al., 2008). SIRT1 expression in human VSMCs was demonstrated to correlate inversely with donor age (Thompson et al., 2014). This was associated with functional defects, such as reduced migratory capacity and increased senescence (Thompson et al., 2014). Interestingly, Ang II infusion significantly decreased the expression of SIRT1 in mouse aorta, which was associated with increased blood pressure and elevated vascular remodeling. Importantly, a VSMC-specific SIRT1 transgene attenuated both Ang II-induced increases in blood pressure and vascular remodeling (Gao et al., 2014a). These recent studies point to the potential of therapeutically improving SIRT1 function in VSMCs to treat hypertension.

### C. Noncoding RNA

Noncoding RNAs comprise many functional RNA transcripts that are not transcribed into proteins, but regulate the transcription, stability, or translation of protein-encoding genes (Ling et al., 2013). Noncoding RNAs were once assumed only to regulate generic functions of cells, such as transcription, translation, and splicing. It is now recognized, however, that a wide variety of noncoding RNA transcripts are transcribed and have diverse biologic activity. The complex nature of this RNA-based regulatory network may partially explain the vast diversity of the characteristics of mammals, despite possessing relatively similar proteomes (Mattick and Makunin, 2006). In addition, epigenetic mechanisms, such as DNA methylation, can regulate the expression of noncoding RNAs, adding a further level of complexity (Liang et al., 2009). This section discusses microRNAs and long noncoding RNAs, which function in the broadly termed RNA silencing machinery.

#### 1. MicroRNAs.

MicroRNAs (miRs) are a family of short (21–25 nucleotide) RNAs, which typically negatively regulate protein translation by direct binding to the 3′ untranslated (3′ UTR) region of mRNA targets. The first miR, lin-4, was discovered to have a role in *Caenorhabditis elegans* larval development more than 20 years ago (Lee et al., 1993). To date, over 30,000 miRs have been discovered in 206 species, including 2578 in humans (van Rooij and Kauppinen, 2014). It is now certain that miRs play an important role in many cellular and developmental processes. Crucially, miRs are dysregulated in many pathophysiological conditions, including cardiovascular disease.

**Fig. 10.**
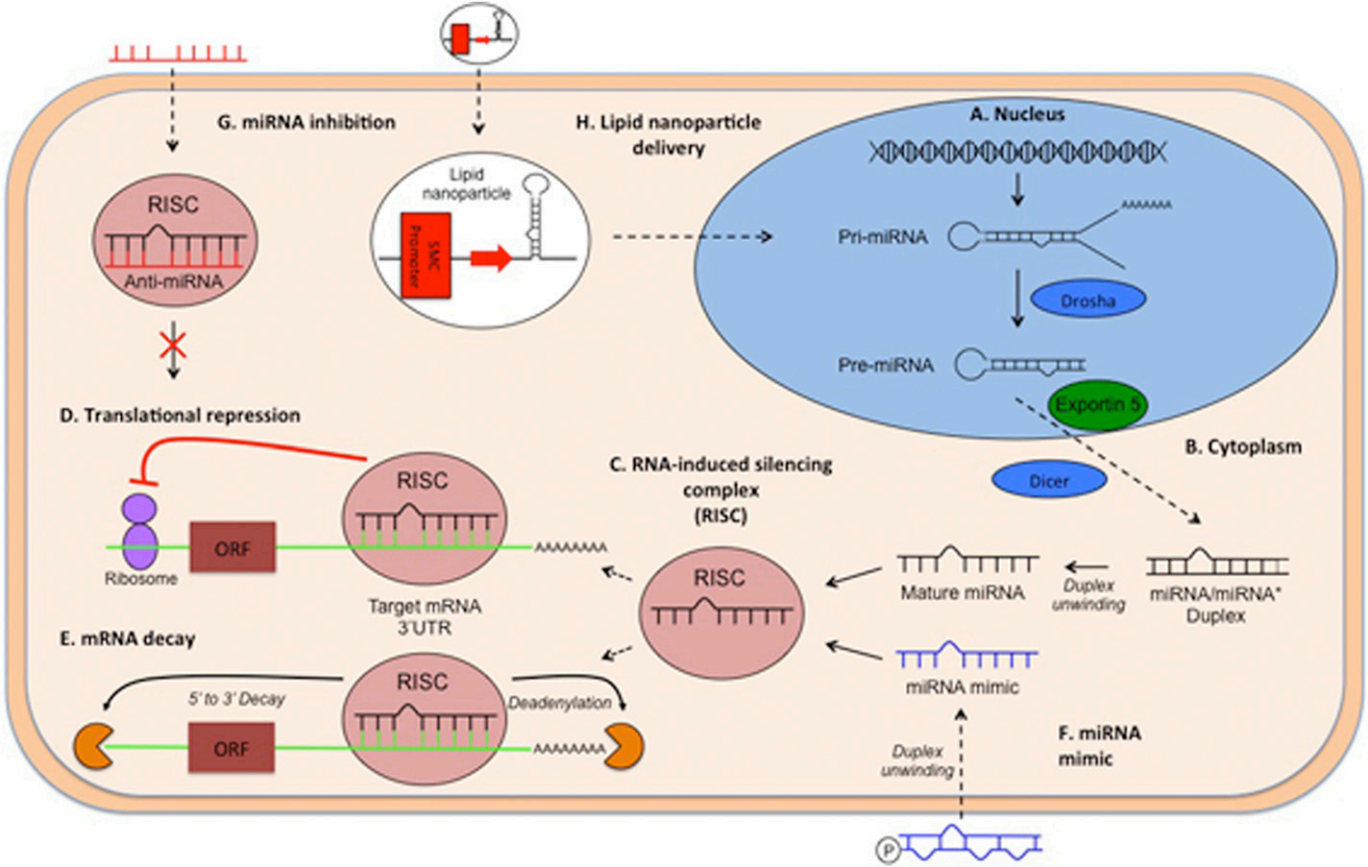
Summary of the main mechanisms of miR biogenesis and function and avenues for pharmacological intervention. (A) MiRs are first transcribed in the nucleus, primarily from introns located in both coding and noncoding DNA, into long primary transcripts termed pri-miRNAs, then are processed in the nucleus by the RNAse III enzyme Drosha into a shorter (∼70 nucleotide) hairpin duplex termed pre-miRs. (B) After export to the cytoplasm by the dsRNA-binding protein Exportin 5, pre-microRNAs are processed by an RNAse III enzyme, Dicer, into a short (21–25 nucleotide) duplex. (C) The miR duplex is unwound, primarily leading to preferential incorporation of a single strand onto Argonaute protein to form the RNA-induced silencing complex (RISC). A short sequence (6–8 nucleotides) at the 5′ end of the miR, known as the "seed" sequence, targets the RISC to complimentary sequences in the 3′ UTR of target mRNAs. (D) Translational repression, through the blocking of translation initiation or recruitment of translational blocking proteins, and/or (E) mRNA decay, via 5′ to 3′ decay and deadenylation of the poly (A) tail. (F) miR mimics are designed as RNA duplexes composed of a passenger strand, chemically modified (e.g., phosphate addition) to permit entry to the cell and subsequent unwinding after entry (i.e., by containing several nucleotide mismatches), and a guide strand, which consists of an identical sequence to the endogenous miR. (G) An anti-miR is designed to be complimentary to the endogenous miR of interest, thus inhibiting target mRNA binding, which therefore increases translation of the target mRNA. (H) A lipid nanoparticle delivery system can induce expression of a miR in specific cell types [e.g., in smooth muscle cells by expressing the miR under the influence of a smooth muscle cell (SMC) promoter].

MicroRNAs are transcribed in the nucleus by RNA polymerase II to long primary transcripts (Lee et al., 2002) (Fig. 10). They are subsequently processed by the RNAse III enzyme Drosha (Lee et al., 2003) in the nucleus to a 70 nucleotide hairpin structure termed preliminary miRNAs (pre-miRNA). After export into the cytoplasm, pre-miRNAs are further processed by a separate RNAse III enzyme, Dicer (Hutvagner et al., 2001), into a 21–25 nucleotide duplex. The mature miR strand is then incorporated onto Argonaute protein to form the RNA-induced silencing complex (RISC) (Hammond et al., 2001; Hutvagner et al., 2001), leading to unraveling and degradation of the complimentary strand (Khvorova et al., 2003; Schwarz et al., 2003). The miR then guides the RISC to complimentary sequences in the 3′ UTR of target mRNA (Lee et al., 1993). In plants, the near-perfect complementarity between the miR and target sequence promotes mRNA cleavage of the target, akin to the mechanism for siRNA-induced silencing (Hake, 2003). In mammals, however, this level of complementarity is rare. In contrast, association of a 6–8 nucleotide ("seed") sequence with the 3′ UTR of target mRNA (Lewis et al., 2005) leads to repression of mRNA translation through inhibition of translation initiation and/or promotion of mRNA decay (reviewed by (Ameres and Zamore, 2013)).

##### a. Dicer knockout mice.

There are several excellent reviews that discuss the role of miRs in vascular structure, function, and disease (Yu et al., 2014; Gupta and Li, 2015; Maegdefessel et al., 2015a; Marques et al., 2015). The current review will mainly focus on miRs that have a role in regulating vascular smooth muscle cell (VSMC) contractility, thus influencing the risk of hypertension. The importance of miRs in vascular smooth muscle was demonstrated in experiments using smooth muscle-specific Dicer KO mice. Knockout of Dicer in vascular smooth muscle (VSM) induced embryonic lethality, which was associated with widespread hemorrhaging, loss of contractile function, and reduced VSMC proliferation (Albinsson et al., 2010). A further study using a tamoxifen-inducible SMC-specific knockout of Dicer, suggested miRs are necessary for blood pressure regulation and contractile function (Albinsson et al., 2011). Blood pressure was reduced after Dicer knockdown, with no change in cardiac dimensions. This was associated with impairment of both receptor- and calcium-mediated contraction of small mesenteric arteries and reduction of expression of contractile proteins (Albinsson et al., 2011). Furthermore, the stretch-dependent increase in contractile gene expression in the portal veins of mice was dependent on Dicer expression (Turczynska et al., 2013). Loss of the miR-143/-145 cluster is thought to be at least partially responsible for this reduced vascular contractility (Boettger et al., 2009; Norata et al., 2012; Dahan et al., 2014). Pathways that promote the vascular smooth muscle contractile phenotype, such as Notch and bone morphogenic protein signaling, function partially through promoting the expression of miR-143/-145 (Boettger et al., 2009; Albinsson et al., 2010; Boucher et al., 2011; Davis-Dusenbery et al., 2011; Norata et al., 2012; Turczynska et al., 2013; Dahan et al., 2014). In addition to miR-143/-145, the expression of miR-21 (Kang et al., 2012) and miR-24 (Chan et al., 2010) is similarly induced by bone morphologic protein (BMP) signaling, promoting the contractile phenotype by targeting Programmed Cell Death 4 and Tribbles-like protein-3, respectively.

The Dicer KO model has also demonstrated an important role for miRs in the regulation of small arterial myogenic tone, therefore modulating peripheral arterial resistance, which is raised in hypertension. Myogenic tone of mesenteric arteries was abolished from Dicer KO mice, which was associated with a loss of calcium influx through the L-type calcium channel (Bhattachariya et al., 2014). It is therefore possible that the aberrant expression of miRs may increase the development of myogenic constriction of small arteries, thereby increasing peripheral resistance and systemic blood pressure.

##### b. Regulation of vascular smooth muscle cell contractility.

Several miRs have been found to posttranscriptionally modulate the expression of VSMC contractile proteins. Overexpression of miR-143/-145 in VSMCs increases the expression of contractile proteins, such as SM-MHC, calponin, and SM22*α*, through the targeted downregulation of KLF4 and 5 (Albinsson et al., 2010, 2011; Boucher et al., 2011; Davis-Dusenbery et al., 2011; Norata et al., 2012; Turczynska et al., 2013; Chettimada et al., 2014; Dahan et al., 2014; Riches et al., 2014). VSMCs from Type 2 diabetic rats (Chettimada et al., 2014) and humans (Riches et al., 2014) display an increased expression of miR-145, which contributes to the enhanced expression of vascular contractile proteins in Type 2 diabetes. Myocardin increases the expression of miR-1 in human aortic SMCs, which blocks the expression of the contractile proteins *α*-SMA and SM22, possibly acting as a negative feedback mechanism to counter myocardin-induced increase in contractility (Jiang et al., 2010). Additionally, although miR-21 may promote the contractile phenotype, it may also negatively regulate VSMC contraction through the targeted downregulation of myosin phosphatase, Rho-interacting protein, and cofilin-2 (Kotlo et al., 2011).

##### c. Regulation of vascular smooth muscle cell ion channels.

MicroRNAs have also been demonstrated to posttranscriptionally regulate the expression of VSMC ion channels, thereby influencing vascular contractility and thus blood pressure regulation. For example, miR-145 indirectly increases VSM contractility by targeted downregulation of CamKII*δ*, which results in increased expression of the *α*_1C_ subunit of the L-type calcium channel (Turczynska et al., 2012). Similarly, miR-328 has been demonstrated to directly target the *α*_1C_ subunit of the L-type calcium channel (Guo et al., 2012). Overexpression of the miR-424/322 resulted in decrease in cyclin D1 and calcium-regulating proteins calumenin and stromal-interacting molecule 1, which reduces store-operated calcium entry in human and rat VSMCs (Merlet et al., 2013). Further studies have elucidated a role for miR-9a-3p (Li et al., 2015) and miR-190 (Li et al., 2014b) in the downregulation of the VSMC K_ATP_ and potassium channel Kv7.5, respectively, causing an increase in contractility.

##### d. Regulation of the extracellular regulated kinase pathway.

MicroRNAs have also been implicated in the downregulation of proteins involved in the ERK 1/2 signaling pathway in proliferating, but not contractile, VSMCs. The miRs-21 (Stein et al., 2014), -31 (Liu et al., 2011), -132 (Choe et al., 2013), and -155 (Yang et al., 2015) all affect ERK activation. It remains to be determined if those miRs similarly affect ERK activation in fully differentiated VSMCs.

#### 2. Long Noncoding RNAs.

Long noncoding RNAs (LncRNAs), arbitrarily classified as longer than 200 nucleotides, have a role in regulating the expression of neighboring genes. LncRNAs have a much more widespread mode of action than miRs, and their function cannot currently be inferred from sequence or structure (Mercer et al., 2009; Rinn and Chang, 2012). LncRNAs can recruit chromatin-remodeling complexes to epigenetically regulate specific genomic loci. They can also influence transcriptional regulation of genes by recruiting RNA binding proteins to gene promoters, acting as cofactors to modulate transcription factor and RNA polymerase II activity. Finally, LncRNAs can also regulate posttranscriptional processing events, by recognizing complimentary sequences of RNA. Like miRs, they have the ability to bind to mRNA targets, affecting their translation and degradation, but they can also influence other posttranscriptional processes such as splicing, editing, and transport. For a deeper description of LncRNA function the reader may refer to the following references (Mercer et al., 2009; Rinn and Chang, 2012).

Although the field is newer than that for miRs, LncRNAs are emerging as potentially important players in cardiovascular disease (recently reviewed by Uchida and Dimmeler, 2015 and Miano and Long, 2015). Through RNA sequencing approaches in human coronary arteries, VSMCs have been demonstrated to express several LncRNAs, including smooth muscle and endothelial cell-enriched migration/differentiation-associated long noncoding RNA (Bell et al., 2014). Depletion of endothelial cell-enriched migration/differentiation-associated long noncoding RNA in VSMCs was associated with decreased expression of myocardin and other contractile genes and increased expression of the promigratory proteins MDK and PTN, suggesting a role in maintenance of the contractile phenotype (Bell et al., 2014). The Lnc-Ang362 was increased in Ang II-treated rats, which was associated with an increased VSMC proliferation. Interestingly, Lnc-Ang362 was cotranscribed with miR-221 and -222 and was required for their expression (Leung et al., 2013). This study provided evidence for the regulation of LncRNAs by Ang II, which may have important implications in the pathogenesis of Ang II-associated cardiovascular diseases, such as hypertension (Leung et al., 2013).

Recent evidence suggested the expression of LncRNAs may affect VSMC proliferation and apoptosis, therefore influencing the risk of aortic aneurysm (He et al., 2015) and atherosclerosis (Congrains et al., 2012a; Bayoglu et al., 2014; Li et al., 2014c; Vigetti et al., 2014; Wu et al., 2014). For example, LncRNA-p21 was demonstrated to inhibit cell proliferation and neointimal hyperplasia by releasing the mouse double minute 2 repression of p53 (Wu et al., 2014). Importantly, this LncRNA was downregulated in atherosclerotic plaques from ApoE^−/−^ mice (Wu et al., 2014). Furthermore, the expression of the LncRNA ANRIL was influenced by several atherosclerosis-associated single nucleotide polymorphisms (SNPs) in the 9p21 locus (Holdt et al., 2011; Congrains et al., 2012a,b; Bayoglu et al., 2014). These data alert one to the possibility that previous genome-wide association studies (GWAS) may have misinterpreted SNPs in regions that encode nonprotein coding RNA as without effect on disease risk.

#### 3. Strategies to Regulate microRNAs in Vascular Disease.

MicroRNAs continue to provide great therapeutic potential, and the strategies to alter miR function will be discussed below. Because the functions of LncRNAs in VSM biology are still being determined, approaches to modify their function have not yet been extensively examined in this setting.

One of the benefits of miRs is that they are strongly conserved in species (albeit potentially targeting differing mRNA targets), thus aiding the design of preclinical studies to determine their efficacy and safety. In addition, miRs have the potential to target many members of the same molecular pathway and as a result having a greater combined effect than siRNAs, which typically target only a single gene. For example, the miR-200 family has been implicated in regulating many targets that control actin filament organization and dynamics (Bracken et al., 2014). Furthermore, miR therapy will not completely knock down their target protein’s expression. Rather, they will result in a "fine-tuning" effect on target protein expression, making them an attractive proposition for therapeutic intervention.

There are two main techniques to modulate miR activity in vivo (reviewed in Ling et al., 2013; van Rooij and Kauppinen, 2014) (Fig. 10): 1) restoring the expression of a downregulated miR by use of a miR-mimic and 2) inhibiting the activity of an abnormally expressed miR by use of an anti-miR. A miR-mimic is a synthetically designed RNA duplex, containing a passenger strand, chemically modified to enhance uptake and disengage once inside the cell, and a guide strand, which is identical to a miR of interest. Conversely, an anti-miR is designed to be complementary to the endogenous miR and therefore binds and inhibits it from acting on its mRNA target. Several modifications, such as locked nucleic acid and phosphodiester additions, have improved the in vivo stability of anti-miRs.

The great strength of miRs, their ability to target multiple mRNAs, is also their greatest weakness because they could potentially result in many off-target effects. In addition, miR mimics often increase the miR concentration to supraphysiologic levels, again increasing the risk of off-target actions. To lower such risk, viral constructs have been developed to re-express the miRs to endogenous levels. Recently, lipid nanoparticle delivery has proved promising to deliver miR regulators to target cells and reduced delivery to nontarget cells (Fig. 10). For instance, anti-miR-145 therapy in rats with Sugen-5416/hypoxia-induced pulmonary arterial hypertension (PAH) using the Star:Star-mPEG delivery system, reduced the severity of pulmonary hypertension without significant off-target effects (McLendon et al., 2015).

Extracellular vesicles, such as exosomes, microvesicles, and apoptotic bodies, allow long-distance delivery of cellular information, including noncoding RNA. They recently emerged as important regulators of cardiovascular diseases, such as atherosclerosis (recently reviewed by Das and Halushka, 2015) and may serve as important diagnostic and prognostic biomarkers for these conditions.

##### a. Pulmonary hypertension.

Several miRs have been found to contribute to clinical and experimental pulmonary hypertension, which is characterized by enhanced proliferation and constriction of pulmonary artery smooth muscle cells (PASMCs). For example, miR-130a promotes PASMC proliferation through the negative regulation of its targets CDKN1A and growth arrest-specific homeobox (Wu et al., 2011; Brock et al., 2015). Interestingly, the miR-130/-301 family also regulates the expression of peroxisome proliferation receptor gamma, which influences the expression of many vasoactive factors, such as endothelin-1 (Bertero et al., 2015). The expression of miR-190 was augmented in PASMCs of the hypoxic rat, which acts as a model for pulmonary hypertension. Vasoconstriction to both potassium chloride and phenylephrine was enhanced after transfection of pulmonary arterial rings with a miR-190 mimic. The same study demonstrated that miR-190 downregulates the protein expression of the potassium channel Kv7.5, which results in raised intracellular calcium in PASMCs (Li et al., 2014b). The miR-328 has been demonstrated to directly target the *α*_1C_ subunit of the L-type calcium channel and was reduced in the pulmonary artery from patients with pulmonary hypertension, thus enabling an increase in vasoconstriction (Guo et al., 2012).

##### b. Systemic hypertension.

There have been fewer animal studies focusing on the role of miRs in the development of essential hypertension. Clinical studies, however, have highlighted the possibility of therapeutic intervention to prevent or treat systemic hypertension. A study from Greece found that the expression of miR-145, -143, and -133 was upregulated, and miR-21 and -1 were downregulated in the peripheral blood of patients presenting with essential hypertension (Kontaraki et al., 2014). These miRs are all important regulators of VSMC phenotype plasticity. A further study in a Chinese population identified the SNP rs12731181 (A to G) in the prostaglandin F_2_*α* receptor that was more prevalent in hypertensive individuals (Xiao et al., 2015). This polymorphism reduces the likelihood of miR-590-3p binding, thus increasing prostaglandin F_2_*α* receptor expression and enhancing prostaglandin-mediated contractility of VSMCs (Xiao et al., 2015).

##### c. Other vascular diseases.

Several studies have demonstrated the feasibility of miR therapies to slow the development of abdominal aortic aneurysms. In particular, the miR-29 family has been heavily implicated in aneurysm development in mouse models. Murine in vivo delivery of miR-29 was found to decrease elastin mRNA and increase MMP activity and aneurysm development (Boon et al., 2011; Jones et al., 2011; Maegdefessel et al., 2012; Merk et al., 2012). Importantly, blockade of miR-29 reduced aneurysm development in Ang II-infused mice (Boon et al., 2011), a mouse model for Marfan syndrome (Merk et al., 2012), and ApoE^−/−^ mice (Maegdefessel et al., 2012), signifying its therapeutic potential. In addition, miR-21 (Maegdefessel et al., 2012), -712/-205, and -195 (Zampetaki et al., 2014) have all been demonstrated to promote aneurysm development, whereas miR-24 (Maegdefessel et al., 2015b) slows aneurysm progression in mouse models.

Interestingly, miR-29 was found to increase with aging in the aorta from mice, which correlated with aneurysm development (Boon et al., 2011). The targets affected by miR-29 are strongly implicated in aortic stiffness, which is also known to increase with age. Few studies have focused on the role of miRs in vascular stiffness. A clinical study found that two SNPs, rs978906 (A allele) and rs9808232 (C allele), were associated with high pulse wave velocity in 856 individuals in a Chinese population (Liao et al., 2015). These polymorphisms rendered the expression of the aortic stiffness-associated ROCK2 (Noma et al., 2007) less responsive to changes in miR-1183 expression, thus enhancing ROCK2 expression (Liao et al., 2015). In addition, miR-145 was strongly reduced in ApoE^−/−^ mice, which was associated with an increase in collagen and stiffer arteries (Kothapalli et al., 2012).

These studies demonstrate the capacity of miR therapeutics to improve treatments for vascular complications such as hypertension, abdominal aortic aneurysm, and arterial stiffness. Indeed, anti-miR-33 (atherosclerosis, Regulus Therapeutics, (Carlsbad, CA)), anti-miR-92 (peripheral artery disease, miRagen Therapeutics Boulder, CO), and anti-miR-145 (vascular occlusion, miRagen Therapeutics) have already reached preclinical trials. This has clearly been a rapidly expanding field and we expect to see many further developments, including approaches to target LncRNAs, in the coming years.

## VIII. Vascular Smooth Muscle Diseases and Their Treatments

### A. Review of Current Therapies and Their Targets

Excess vasoconstriction and the resulting increase in vascular tone and SVR are an important contributing factor to the pathogenesis of essential hypertension. CCBs, ACE inhibitors, ARBs, and direct vasodilators (i.e., hydralazine) all target the smooth muscle cell to decrease vascular tone and blood pressure. CCBs decrease the activation of SM myosin by decreasing intracellular Ca^2+^, whereas ACE inhibitors and ARBs will decrease the activation of the RhoA/Rho kinase signaling pathway to reduce Ca^2+^ sensitization. K^+^ channel openers produce vasodilatation; nicorandil acts on BK channels and hyperpolarizes smooth muscle cells (Nelson et al., 1990), whereas minoxidil opens ATP-sensitive K^+^ channels and hyperpolarizes the smooth muscle cells (Wickenden et al., 1991). The mechanism of action for hydralazine, on the other hand, is unclear, but evidence suggests that hydralazine both activates K^+^ channels and also stimulates NO production by the vascular endothelium (Knowles et al., 2004).

In addition to essential hypertension, there are a number of other diseases of the vasculature and diseases with associated abnormal vascular reactivity. These defects in vascular reactivity contribute to morbidity and mortality. In this section, we will outline these diseases, their mechanism, current therapies, and suggest novel targets for both the treatment of essential hypertension as well as these other diseases of the vasculature (Table 1).

**TABLE 1:** Disease-associated vascular abnormalities In addition to hypertension, there are a number of diseases with abnormalities of the vasculature that contribute to patient morbidity and mortality. The vascular abnormality, possible mechanisms as well as therapies and potential new, novel targets for therapy are listed and discussed in detail in the text.

Disease	Abnormality	Possible Mechanisms	Treatments
Heart Failure	Resting Vasoconstriction,	Defect in NO/cGMP mediated vasodilatation; ?Decrease in LZ+ MYPT1	Vasodilators (ACE inhibitors, ARBs, hydralazine, nitrates)
	Decrease in sensitivity to NO		
Idiopathic Pulmonary Hypertension	Pulmonary Vascular Vasoconstriction &	Proliferation of pulmonary SMCs, Defect in NO/cGMP mediated vasodilatation. ?Decrease in LZ+ MYPT1 and increase in NM myosin	Prostaglandins (Epoprostenol), Phosphodiesterase inhibitors (Sildenafil), Guanylate cyclase stimulators (Riocigaut), Endothelin antagonists (Bosentan), NO inhalation & Rho kinase inhibitors (Fasudil)
	Decrease in sensitivity to NO		
Portal Hypertension	Sensitivity to vasodilators increased & to vasoconstrictors decreased	?Changes in isoform expression of contractile proteins which influence both smooth muscle activation and relaxation	Management of fluid status
Raynaud’s Phenomenon	Transient vasospasm of digital vessels	Altered reactivity of vascular smooth muscle	Keeping digits warm, CCBs
Pre-eclampsia/Pregnancy Induced Hypertension	Increase in vascular tone	Altered reactivity to RhoA, PKC & Ca^2+^, decrease NO	Antihypertensives (non-fetotoxic)

### B. Other Major Vascular Diseases Including Analysis of Current Therapies and Novel Targets

#### 1. Heart failure.

Considerable evidence from both human subjects and animal models have shown that HF is associated with an impaired vasodilatory response to acetylcholine, via an endothelium-dependent mechanism, as well as to nitroglycerin, via an endothelial-independent mechanism (Kaiser et al., 1989; Kubo et al., 1991; Katz et al., 1993). Both MYPT1 LZ+ expression and the vasodilatory response to NO/cGMP has been demonstrated to decrease in HF (Karim et al., 2004; Chen et al., 2006; Ararat and Brozovich, 2009; Han and Brozovich, 2013), which demonstrates that the fall in LZ+ MYPT1 expression contributes to the decrease in sensitivity to NO-mediated vasodilatation associated with HF. Additionally, treatment of HF with either ACE inhibitors (Chen et al., 2006) or ARBs (Ararat and Brozovich, 2009) has been demonstrated to preserve the normal expression of the LZ+ MYPT1 isoform and sensitivity to NO/cGMP-mediated vasodilatation. These results are similar to those of Abassi et al., 1997; this group demonstrated that both endothelium-dependent and -independent responses in renal blood flow were impaired in HF rats compared with control, and Ang II receptor blockade normalized these vasodilatory responses.

The mechanism for the regulation of LZ+/LZ− MYPT1 expression by Ang II-induced activation of the AT1 receptor is unknown, but evidence suggests that both the activation of p42/44 MAPK (Ararat and Brozovich, 2009) and Tra2*β* (Shukla and Fisher, 2008) are important. Ang II produces vasoconstriction through its effect on both the endothelium and vascular smooth muscle (Nickenig and Harrison, 2002). Additionally, Ang II activates nuclear factor *κ*B to produce a proinflammatory state by inducing expression of interleukin (IL)-6 and tumor necrosis factor (TNF)-*α* (Dzau, 2001; Nickenig and Harrison, 2002) and also activates membrane NADH/NADPH oxidases, generating reactive superoxide anions, which increase NO catabolism and therefore decrease NO bioavailability (Griendling et al., 1994). In heart failure, inhibition of Ang II signaling counter these deleterious effects and could contribute to the reduction in cardiovascular morbidity and mortality observed with ACE inhibitors and ARBs (Pfeffer et al., 1992; Yusuf et al., 1992). Furthermore, similar beneficial effects on survival have not been observed with other vasodilators such as prazosin, although prazosin has also been shown to improve cardiac index and fractional shortening for heart failure patients (Awan et al., 1977; Miller et al., 1977), which suggests that ACE inhibitor have other beneficial effects beyond reducing afterload and improving left ventricular remodeling.

In the setting of advanced heart failure, marked activation of the renin-angiotensin system and worsening functional status have been associated with a progressive elevation of the proinflammatory marker TNF-*α* (Levine et al., 1990; Torre-Amione et al., 1996), which has been demonstrated to contribute to endothelial dysfunction and also an increase in vascular tone that is produced by a RhoA/Rho kinase-mediated inhibition of MLC phosphatase (Parris et al., 1999; Mann, 2002). Additionally, the inflammatory marker IL-1*β*, which is also elevated in heart failure, increases Ser850 MYPT1 phosphorylation to inhibit MLC phosphatase (Kandabashi et al., 2000; Mann, 2002). It has been demonstrated that impaired endothelial and vascular smooth muscle dysfunction are associated with a poor prognosis in heart failure (Mann, 2002). Potentially IL-1*β* and TNF-*α* could be involved in the regulation of MYPT1 isoform expression, and defining the role of inflammatory cytokines for the regulation of MYPT1 isoform expression could reveal a novel therapy to reverse the vascular dysfunction and improve prognosis in heart failure.

In addition to inhibiting the RhoA/Rho kinase pathway (Arner and Pfitzer, 1999; Somlyo and Somlyo, 2003), ACE inhibitors and ARBs also preserve the LZ+ MYPT1 isoform expression (Chen et al., 2006; Ararat and Brozovich, 2009). The variability in LZ+ MYTP1 expression among different vascular smooth muscle cells and the changes that occur during heart failure could provide an explanation as to why racial differences play an important role in predicting responses to ACE inhibitor therapy compared with the combination of vasodilators and nitrates (Carson et al., 1999). There are racial variations in plasma norepinephrine and renin (Carson et al., 1999), and this could influence both MYPT1 isoform expression as well as MLC phosphatase activity. These factors could explain the more significant blood pressure and mortality reduction seen in white compared with black patients (Carson et al., 1999). MYPT1 polymorphisms may also exist, which could contribute to the diversity of symptoms in patients with similar reductions in LVEF. Hence, the ability of ACE inhibitors and ARBs to alter the vascular smooth muscle cell phenotype could contribute to the improvement in survival in patients with heart failure (Pfeffer et al., 1992; Yusuf et al., 1992; Granger et al., 2003), and thus enhancing LZ+ MYPT1 expression and normalizing the vascular response to NO may represent another novel target for the treatment of heart failure.

#### 2. Pulmonary hypertension.

Pulmonary arterial hypertension (PAH) is a rare, incurable disease with a poor prognosis (Geraci et al., 2001a,b; Archer et al., 2010). In patients with PAH, the pulmonary vasculature is characterized by a resting vasoconstriction and an abnormal response to NO, and in the majority of patients, NO-mediated vasodilatation is severely blunted (Sitbon et al., 1998; McGoon et al., 2004). Clinically, the initial assessment of patients with PAH includes the evaluation of the pulmonary response to NO (Badesch et al., 2004; Barst et al., 2004; McGoon et al., 2004) prognosis is improved if NO decreases pulmonary arterial pressure and PVR by 20% (Sitbon et al., 1998; Barst et al., 2004). However, less than 10% of patients with PAH demonstrate a significant vasodilatory response to NO (Barst et al., 2004), and although there are a number of proposed mechanisms for the progressive increase in PVR (Stenmark et al., 2006; Archer et al., 2010; Farkas et al., 2011), the molecular mechanism that results in the lack of vasodilatation to NO in PAH is unknown.

There are two components that produce PAH: the extent of the structural changes (smooth muscle cell proliferation, smooth muscle cell hypertrophy, and the deposition of matrix proteins within the media of pulmonary arterial vessels (Stenmark et al., 2006; Archer et al., 2010; Farkas et al., 2011) and excess vasoconstriction (Oka et al., 2007; Archer et al., 2010). Structural changes have been documented in animal models of PAH, which have demonstrated that chronic hypoxia leads to pulmonary vascular remodeling that increases PVR and decreases the vasodilatory response to NO (reviewed in Stenmark et al., 2006; Archer et al., 2010; Farkas et al., 2011). However, despite the fact that for PAH the target and aim of all current therapeutic agents [prostaglandins (epoprostenol), phosphodiesterase inhibitors (sildenafil), guanylate cyclase stimulators (riocigaut), endothelin antagonists (bosentan), NO inhalation and Rho kinase inhibitors (fasudil)] is the smooth muscle cell to reduce pulmonary vascular resistance, the fundamental changes in the pulmonary smooth muscle contractile phenotype that contribute to the excess vasoconstriction, or the increase in PVR, as well as the decrease in vasodilatory response to NO, are poorly characterized. Evidence supports a contribution of changes in the pulmonary smooth muscle contractile phenotype in PAH; an increase in the expression and activity of Rho kinase has been demonstrated to contribute to the pathogenesis of PAH (Connolly and Aaronson, 2011), and Rho kinase inhibition reduces pulmonary pressure in some animal models of PAH (Oka et al., 2007; Archer et al., 2010). The importance of the NO/cGMP signaling pathway for the pathogenesis of PAH is highlighted by data demonstrating that PKG KO mice develop pulmonary hypertension (Zhao et al., 2012), and mice with mutations in the PKG LZ domain, which disrupts PKG interaction with MYPT1 (Surks et al., 1999, 2003; Huang et al., 2004; Yuen et al., 2011; Yuen et al., 2014), develop progressive increases in pulmonary pressures and right ventricular hypertrophy (Ramchandran et al., 2014). Furthermore, activation of guanylate cyclase has been demonstrated to improve exercise capacity and reduce pulmonary pressures in patients with PAH (Zhao et al., 2012; Ghofrani et al., 2013a,b; Ramchandran et al., 2014). All these studies are consistent with a defect in NO/cGMP signaling contributing to the pathogenesis of pulmonary hypertension, but do not isolate the step in the NO signaling pathway that produces PAH.

In smooth muscle, investigators have demonstrated that NM myosin (Fig. 7) contributes to the sustained force response (Morano et al., 2000; Lofgren et al., 2003) and a decrease in NM myosin expression will result in a decrease in vascular tone (Yuen et al., 2009). Additionally, changes in the sensitivity to NO-mediated vasodilatation are due to changes in the expression of LZ+/LZ− MYPT1 (Huang et al., 2004). Thus, an increase in NM myosin and decrease in LZ+ MYPT1 expression will produce abnormalities of pulmonary vascular reactivity and may participate in the molecular mechanism that produces PAH (Konik et al., 2013). Konik et al. (2013) demonstrated that with the development of severe PAH, there is a 3.5-fold increase in NM myosin expression, which could contribute to the prolongation in the rates of both force activation and relaxation, as well as the increase in force maintenance. Similarly, an increase in NM myosin expression has also been reported for hypoxia induced PAH (Packer et al., 1998), and therefore drugs that target NM myosin expression or activation could represent a new class of therapeutic agents for the treatment of PAH.

Konik’s data also demonstrated that a decrease in LZ+ MYPT1 expression is associated with severe PAH (Konik et al., 2013). The sensitivity of smooth muscle to NO is regulated, in part, by relative LZ+ MYPT1 expression (Huang et al., 2004). In severe PAH, these data would suggest that the decrease in LZ+ MYPT1 expression would result in a decrease in the sensitivity of the pulmonary vasculature to NO. Similarly, a decrease in LZ+ MYPT1 expression has been reported for cultured pulmonary SMC exposed to hypoxia (Singh et al., 2011). Additionally, the transition from a phasic to tonic SM contractile phenotype has been suggested to be associated with a decrease in the ratio of LZ+ MYPT1/MYPT1 expression (Fisher, 2010). Tonic contractile properties would produce an increase in vascular tone, similar to that observed in patients with PAH. These results are consistent with a decrease in LZ+ MYPT1 expression contributing to the pathogenesis of PAH; a decrease in LZ+ MYPT1 expression would reduce the sensitivity to cGMP, and riociguat treatment (Ghofrani et al., 2013a,b) would increase cGMP to counteract and overcome the decrease in sensitivity to NO to reduce PVR and pulmonary pressures. If a decrease in LZ+ MYPT1 expression contributes to the molecular mechanism for PAH, these data could provide a unifying mechanism to explain the variable response to drugs that act on the NO/cGMP signaling pathway (NO, PDE5 inhibitors, and riociguat), i.e., patients with a mild decrease in LZ+ MYPT1 expression would represent “responders,” whereas patients in which LZ+ MYPT1 expression is severely depressed would not respond to this class of therapeutics. Developing therapeutics that increase LZ+ MYPT1 expression should normalize the pulmonary vascular response to NO and decrease PVR, which would represent a novel target for treating patients with PAH.

#### 3. Portal hypertension.

Vascular reactivity is known to be altered in portal hypertension, with increased sensitivity to dilatation and decrease sensitivity to constriction (Benoit et al., 1984; Sikuler et al., 1985; Bomzon and Blendis, 1994; Wu and Benoit, 1994; Payne et al., 2004). The mechanism that contributes to this altered reactivity is unclear, but evidence suggests that splice variant expression of the contractile proteins is modulated by flow and/or pressure (Zhang and Fisher, 2007). Payne et al. (2004) examined the expression of SM MHC, ELC17, and MYPT1 in the portal vein stenosis model of portal hypertension (Benoit et al., 1984). The ligature of the portal vein produces dynamic changes in flow and pressure; there is an initial increase in both splanchnic pressure and flow (Benoit et al., 1984), which fall with the development of porto-systemic shunting. Similar to these dynamic changes in flow, these investigators (Payne et al., 2004) demonstrated that in both the portal veins and mesenteric arteries within 1 day of the increase in portal pressure, although total MYPT1 expression decreased, the expression of the LZ+ MYPT1 isoform increased. These changes in MYPT1 expression were maintained for ∼7 days before returning to preportal hypertension levels by 14 days after the ligature of the portal vein. Similar to MYPT1 isoform expression, ELC17 and SM MHC expression were modulated with an increase in the expression of the slow isoforms (ELC17b and SMA) 3 days after ligature, but SMA expression returned to baseline at day 14.

Others have altered flow in the mesenteric circulation by ligating alternative pairs of second order mesenteric arteries to produce either low or high flow in the upstream first order mesenteric arteries (Zhang and Fisher, 2007). These investigators demonstrated that there was an initial increase in LZ+ MYPT1 expression in both low and high flow vessels, but by 1 month LZ+ MYPT1 expression returned toward baseline in the low flow vessels, whereas in the high flow vessels, LZ+ MYPT1 expression continued to increase. As would be predicted, the sensitivity to NO/cGMP-mediated relaxation paralleled LZ+ MYPT1 expression. Furthermore, total MYPT1 expression rapidly fell in both the low and high flow mesenteric vessels, and pretreatment with an inhibitor of the proteosome attenuated the decrease in MYPT1. These data suggest that the rapid decrease in total MYPT1 is due to degradation by the proteosome (Zhang and Fisher, 2007), which is similar to the results in the mesenteric arterioles in heart failure (Han and Brozovich, 2013). These data demonstrate that flow and/or pressure is also important in regulating the smooth muscle contractile phenotype, and thus vascular reactivity.

#### 4. Raynaud's phenomenon.

Raynaud’s phenomenon is described as a transient cessation of blood flow in the fingers and toes and can be triggered by either cold or emotional stress. The phenomenon is the result of a transient vasospasm of the digital arteries in the hands and/or feet that leads to a cessation of blood flow, which produces pallor, followed by cyanosis and pain due ischemia of the sensory nerves. As the vasospastic attack subsides, blood flow to the digits is restored, and the resulting hyperemia produces a red phase. The estimates of the prevalence of this disease vary, but it is more common in women, and the onset and severity of the disease peaks between menarche and menopause. Treatment of Raynaud’s involves keeping the digits warm and pharmacologic therapy with CCBs can be helpful; however the usefulness of CCBs is often limited by hypotension (Cooke and Marshall, 2005).

There are a number of mechanisms proposed for the transient vasoconstriction of the digital vessels; these have been reviewed (Cooke and Marshall, 2005) and there is evidence supporting roles for hyperactivity of adrenergic nervous system, *α*_2_ adrenoceptors, central stress responses, 5-HT, endothelin, oxidative stress, cyclo-oxygenase, and NO. However, it is interesting that the vasodilation of the digital vessels produced by both sodium nitroprusside and ACh are reduced in patients with Raynaud’s (Morris and Shore, 1996; Cooke and Marshall, 2005), which suggest that the defect lies within the cGMP/PKG signaling cascade. Consistent with this hypothesis are the results that show l-arginine does not restore the normal vasodilatory response to ACh (Khan et al., 1997), which indicates that the vasodilatory response to NO is depressed in Raynaud’s disease, and the mechanism lies beyond the bioavailability of NO within the cGMP/PKG signaling cascade. These data could suggest that a decrease in LZ+ MYPT1 expression in the digital vessels could contribute to the abnormal vascular reactivity.

#### 5. Pre-eclampsia/pregnancy-induced hypertension.

During a normal pregnancy, vascular resistance falls, which is thought to be the results of increased synthesis and activity of NO and prostacyclin (Bird et al., 2003; James and Nelson-Piercy, 2004). However, approximately 5–15% of pregnancies are complicated by hypertension, which increases perinatal morbidity and mortality of both the mother and fetus (Lindheimer, 1993; James and Nelson-Piercy, 2004). Antihypertensive therapy is limited due to the effects on the fetus, i.e., ACE inhibitors and ARBs are fetotoxic (James and Nelson-Piercy, 2004). A number of mechanisms have been proposed for the increase in PVR and blood pressure, including both a decrease in NO produced by endothelial dysfunction and an increase in circulating vasoconstrictors including Ang II, thromboxane, and endothelin (Khalil and Granger, 2002; Bird et al., 2003; Granger et al., 2003). NOS inhibition is commonly used to produce a model of pregnancy-induced hypertension, and studies have suggested that the increase in blood pressure is due to altered vascular reactivity produced by RhoA, PKC, and Ca^2+^ signaling (Khalil and Granger, 2002; Carter and Kanagy, 2003).

Lu et al. (2008) recently examined MYPT1 isoform expression in pregnancy-induced hypertension. These investigators demonstrated that during a normal pregnancy total MYPT1 expression increased, but there was no change in MYPT1 isoform expression. However, during pregnancy-induced hypertension, although there were no changes in MYPT1 expression in mesenteric arteries or the aorta, in uterine arteries, pregnancy-induced hypertension was associated with an increase in LZ+ MYPT1 expression but an ∼50% decrease in total MYPT1 expression. As would be predicted, the increase in LZ+ MYPT1 expression was associated with an increase in the sensitivity of uterine artery relaxation to both sodium nitroprusside and cGMP. The increase in LZ+ MYPT1 expression did not occur when the blood pressure of the pregnant hypertensive rats was normalized with hydralazine. It is interesting to speculate that during pregnancy-induced hypertension the changes in MYPT1 expression are compensatory, and the resulting reduction in uterine vascular resistance maintains blood flow despite the inward remodeling of the arteries (Lu et al., 2008).

### C. Personalized Medicine

A number of studies demonstrate that blood pressure is a genetically determined trait with heritability estimates of 31–68% (Padmanabhan et al., 2012). There are several, rare monogenic syndromes, which are due to abnormalities in renal fluid balance that result in hypertension (Lifton et al., 2001; Ji et al., 2008). However, because the regulation of blood pressure is due to complex interactions of fluid balance, cardiac contractility, and vascular tone, the genetic basis of essential hypertension has yet to be elucidated (Oparil et al., 2003; Padmanabhan et al., 2012). Recently, a number of genome wide-association studies (GWAS) have identified SNPs in a number of genes that could contribute to the development of essential hypertension (Adeyemo et al., 2009; Johnson et al., 2011; Kato et al., 2011; Wain et al., 2011; Havulinna et al., 2013; Tragante et al., 2014). These studies have focused on populations of European decent, but GWAS have examined East Asia (Kato et al., 2011) as well as African American cohorts (Adeyemo et al., 2009). A complete discussion of GWAS methods as well as the results of these studies are beyond the scope of the present review, and we would refer readers to recent reviews on this subject (Ehret, 2010; Padmanabhan et al., 2012). In addition to SNPs, gene expression is also regulated by miR and DNA methylation. The importance of miRs in the regulation of blood pressure is highlighted by a recent study demonstrating that mice lacking miR-142 and miR-145 have reduced vascular tone and blood pressure (Xin et al., 2009). Additionally, hcmv-miR-UL122 is highly expressed in hypertensive patients (Cheng et al., 2009), a number of miRs are increased in hypertensive nephrosclerosis (Wang et al., 2010), and furthermore, renin expression appears to be regulated by mi-RNA-181a and miR-663 renin (Marques et al., 2011). These factors illustrate that SNPs, miRs, and posttranslational modifications all play a role in the development of hypertension.

In addition to revealing candidate genes important in the pathogenesis of essential hypertension, pharmacogenomic GWAS have the potential to reveal the likelihood of a patient to respond to therapy or even to develop a rare adverse drug reaction (Crowley et al., 2009; Johnson et al., 2008). There are several reports of an association between polymorphisms in the *β*_1_-adrenergic receptor gene and the lowering of blood pressure (reviewed in Shin and Johnson, 2007). An association of Ser49Gly and Arg389Gly polymorphisms has been demonstrated to be associated with a significant reduction in blood pressure with *β*-blocker therapy (Johnson et al., 2003), and furthermore, one or both of these SNPs were carried by 54% of Chinese and 44% of whites (Johnson et al., 2003), perhaps suggesting an etiology of the ethnic differences in response to *β*-blocker therapy. The PEAR study (Gong et al., 2012) evaluated the association of 39 SNPs known to be associated with hypertension from GWAS studies with a response to monotherapy with atenolol or HCTZ in 768 hypertensive patients (60% white and 40% black). The response to atenolol therapy was greater in the white patients; six SNPs were associated with a response to atenolol therapy, with greater responses with in those with all six BP lowering alleles. HCTZ lowered blood pressure less in white compared with black patients. In the white patients, three alleles were associated with BP lowering for HCTZ monotherapy, and similarly, the association was strongest for those with all three alleles, but none of these were associated with BP lowering in blacks. Interestingly for HCTZ therapy, one SNP was associated with a BP reduction in white hypertensive patients, whereas it was associated with an increase in BP in the black patients. A G825C polymorphism in the gene encoding the G-protein *β*_3_-subunit has been associated with a response to HCTZ therapy, and this genotype was a stronger predictor of response to diuretics than ethnicity (Turner et al., 2001). There have been two large trials examining polymorphisms in the genes encoding ACE (Arnett et al., 2005) and the AT1 receptor (Brunner et al., 2007) and the response to ACE inhibition. No association was found for the insertion/deletion polymorphism in the ACE gene and the response to lisinopril therapy (Arnett et al., 2005), and similarly for all ethnic groups examined, no genetic association was present for the 1166A-C genotype of the AT1 receptor and response to trandolapril. These data suggest that pharmacogenomic GWAS have the potential to identify genotypes that not only contribute to the development of hypertension but will also predict a positive response to antihypertensive therapy. An individual’s genotype could be then used for a personalized approach for selecting an effective antihypertensive with minimal chance of the patient developing an adverse reaction.

### D. Summary of Novel Targets and Potential for Improved Therapies

There is no question that essential hypertension is due, in part, to changes at the level of vascular smooth muscle that lead to excess vasoconstriction and a concomitant increase in SVR and blood pressure. In this review, we suggested have several novel targets for the treatment of essential hypertension. Details and the strategy for the each target can be found in the individual sections. These targets include those that affect Ca^2+^, including 1) downregulation of AKAP150, 2) increasing the expression of the *β*1 subunit of the BK channel, 3) novel BK channel openers, 4) inhibiting or decreasing the expression of STIM/Orai (Ca^2+^ release-activated Ca^2+^ channels), and 5) increasing cleavage of the C terminal of LTCC or increasing CCt expression. One could also consider inhibition of MLCK as a novel target; the soybean isoform of calmodulin (SCaM-4) has been demonstrated to bind Ca^2+^ but not activate MLCK and thus inhibit the activation of smooth muscle (Lee et al., 2000a; Van Lierop et al., 2002). Thus, targeted expression of SCaM-4 or a CaM fragment in vascular smooth muscle should reduce SVR and blood pressure. There are also a number of scaffolds including those for ERK (calponin, SmAV, and paxillin) and MLC phosphatase (M-RIP and Par-4) that could be examined as potential therapeutic targets, which could result in a reduction of vascular tone. Inhibition of the signaling pathways for Ca^2+^ sensitization including Rho kinase and GEF signaling could represent another area for rational drug design, and similarly, activation of pathways leading to Ca^2+^ desensitization including guanylate cyclase and increasing the expression of LZ+ MYPT1 would enhance NO/flow mediated vasodilatation, which should decrease blood pressure. Other attractive targets would be pathways leading to remodeling of the actin cytoskeleton including integrins, focal adhesion proteins, as well as the activation of tyrosine kinases. Targeted delivery of miRs to the vasculature also may prove to be an effective strategy for the treatment of hypertension; miRs could be used as a strategy to change vascular smooth muscle gene program from high force (tonic) and NO unresponsive to a lower force (phasic) and NO responsive and hence produce a reduction in vascular tone and blood pressure.

These novel targets could form the basis for rationale drug design, and could be exploited for development of therapeutic agents that are effective for the treatment of hypertension. For some targets, small molecule inhibitors/activators would appear to be a reasonable choice, whereas for other targets, i.e., to reverse the decreases in MYPT1 expression, adenoviral delivery of MYPT1 to the vasculature could also be used as a strategy to increase the sensitivity to NO or flow-mediated vasodilation, which would decrease in vascular tone and blood pressure.

The contribution of alteration in the vascular phenotype from Ca^2+^ signaling, regulation of the cytoskeleton and contractility, and biomechanics to the pathogenesis of essential hypertension may vary among ethnic groups and/or individuals. Currently, antihypertensive therapy is generally approached by a method that could be best summarized as trial and error: an agent is selected, usually without consideration of the patient’s ethnic background, and if control is not adequate, either the dose is increased or another agent is added.

In this review, we discussed the importance of the vascular phenotype in the mechanism that leads to essential hypertension. Therefore, mechanisms to identify the etiology of the increase in blood pressure in each patient are important for ultimately selecting an individualized and effective therapeutic agent. These future studies will hopefully lead to a more personalized approach to antihypertensive therapy.

## Abbreviations

ACE: angiotensin converting enzyme
AM: rigor state
Ang II: angiotensin II
ARB: angiotensin receptor blocker
AT1: angiotensin type 1
BK: Ca^2+^-dependent K^+^ channels
BP: blood pressure
CaCC: Ca^2+^ gated Cl^−^ channel
CaD: caldesmon
Ca_e_: extracellular Ca^2+^
CaP: calponin
CCB: calcium channel blocker
CCt: C-terminal end of the LTCC
CI: central insert
CICR: Ca^2+^-induced Ca^2+^ release
CO: cardiac output
CRAC: calcium release activated calcium channel
CREB: cAMP response element-binding protein
CVD: cardiovascular disease
EC: endothelial cells
ECM: extracellular matrix
ELC17: 17-kDa essential myosin light chain
eNOS: endothelial nitric oxide synthase
ERK: extracellular regulated kinase
FA: focal adhesion
FAK: focal adhesion kinase
GAP: GTPase activating protein
GEF: guanine nucleotide exchange factor
GWAS: genome wide-association studies
HAT: histone acetylase
HDAC: histone deacetylase
HF: heart failure
IL: interleukin
KLF: Kruppel-like factor
KO: knockout
LTCC: L-type Ca^2+^ channels
LncRNA: long noncoding RNA
LZ: leucine zipper
MHC: muscle myosin heavy chain
miR: microRNAs
MLCK: myosin light chain kinase
MP: myosin phosphatase
NAD: nicotinamide adenine dinucleotide
NM: nonmuscle
NO: nitric oxide
PAH: pulmonary arterial hypertension
PASMC: pulmonary artery smooth muscle cells
PKGI: protein kinase G
pre-miRNA: preliminary miRNA
PWV: pulse wave velocity
RISC: RNA-induced silencing complex
RLC: regulatory myosin light chain
RyR: ryanodine receptors
SHR: spontaneously hypertensive rat
SIRT: sirtuin
SM: smooth muscle
SMA: slow isoform of smooth muscle myosin heavy chain
SMB: fast isoform of smooth muscle myosin heavy chain
SNP: single nucleotide polymorphism
SOCE: store-operated calcium entry
SR: sarcoplasmic reticulum
STIM: stromal interaction molecule
SVR: systemic vascular resistance
TNF: tumor necrosis factor
3′ UTR: 3′ untranslated
VSMC: vascular smooth muscle cells
WT: wild type
